# Investigation of the spatial and temporal long-term hydro-climatic trends in Upper Omo Gibe Basin, Ethiopia

**DOI:** 10.1016/j.heliyon.2025.e42265

**Published:** 2025-01-27

**Authors:** Eyasu Tafese Mekuria, Tamene Adugna Demissie, Fekadu Fufa Feyessa

**Affiliations:** a Faculty of Civil and Environmental Engineering, Jimma Institute of Technology, Jimma University, P.O. Box 378, Jimma, Ethiopia; b Department of Hydraulic and Water Resources Engineering, College of Engineering and Technology, Wolkite University, P.O. Box 07, Wolkite, Ethiopia

**Keywords:** Coefficient of variation, Innovative trend analysis, Modified Mann-Kendall, Upper Omo Gibe Basin, Standard anomaly index

## Abstract

Human activities have changed hydroclimatic components worldwide, affecting agricultural productivity and water resources management. This research examined the spatial and temporal variability of hydroclimatic variables in the Upper Omo Gibe Basin. Modified Mann-Kendall, Senʼs slope, and Innovative Trend Analysis methods were used to study the trends. The maximum statistically significant increasing trend of rainfall (P < 0.05) was observed at Limugenet station (Z = 3.641, S = 16.484), and a decreasing trend of rainfall was observed in Dedo station (Z = −2.732, S = −31.099). A generally decreasing rainfall trend was observed in the Upper Omo Gibe Basin. Temperature showed an upward trend in seasonal and annual observation. In spring, summer, autumn, and winter seasons the maximum significantly increasing trend (P < 0.05) of maximum temperature was 0.25 ^o^C/year, 0.15 ^o^C/year, 0.043 ^o^C/year, and 0.082 ^o^C/year respectively whereas the annual maximum significantly increasing trend was 0.083 ^o^C/year. A significant increase in streamflow (P < 0.05) was observed at the Gibe gauging station (Z = 2.02) and Gilgel Gibe at Assendabo station (Z = 2.02) in the summer season. A significant decrease trend of streamflow has been observed in the Bulbul gauging station in the spring season (Z = −2.20), Megecha gauging station in the summer season (Z = −2.19), and annual (Z = −2.34). A statistically significant increasing and decreasing trend observed was 22 (27.5 %), 43 (53.75 %), 39 (48.75 %), and 5 (10 %) for rainfall, maximum temperature, minimum temperature, and streamflow respectively with the MMK test. Using the ITA method, 30 (37.5 %), 47 (58.75 %), 44 (55 %), and 34 (68 %) for rainfall, maximum temperature, minimum temperature, and streamflow were obtained respectively. This indicates that the ITA method displays stronger significant trends than the MMK method. The results benefit water resource management, drought mitigation, and sustainable agricultural planning in the basin.

## Highlights

• Spatio-temporal trend variability of rainfall, maximum temperature, minimum temperature, and streamflow were investigated.
• A decreasing trend of rainfall has been observed in the Upper Omo Gibe Basin.
• Temperature showed an upward trend in seasonal and annual observation.
• The ITA method displays stronger significant trends than the MMK method.
• In general, the Upper Omo Gibe Basin experienced hydroclimatic variability.

## 1 Introduction

The impacts of climate change on hydroclimatic systems have resulted in varying magnitudes and frequencies of hydrological extremes, affecting water resources management, agricultural practices, and economic development [1,2]. Climate change and human-driven activities have led to changes in the hydrological attributes of rivers globally [3]. Nearly every region in the world is experiencing shifts in both the long-term variability and short-term fluctuations of water resources as a result of climate change [4]. In Sub-Saharan Africa, climate change and variability are considered the primary threats to agricultural production and food security, especially in areas that depend on rain-fed agriculture [[5], [6], [7]]. According to the Intergovernmental Panel on Climate Change (IPCC) Assessment Report Six (AR6) [8], Sub-Saharan African countries continue to experience a warming trend. Between 1991 and 2021, the mean rate of change was around +0.3 ^o^C/decade, higher than the rates observed in previous decades: +0.2 °C decade from 1961 to 1990, 0.04 °C/decade between 1931 and 1960 and + 0.08 °C/per decade from 1901 to 1930. Whereas, the rainfall in this region exhibits significant variability across both spatial and temporal scales due to complex topographical features and circulation patterns [9,10].

Ethiopia is one of the most populated countries in the region. Agriculture is the cornerstone of the Ethiopian economy. It generates about 50 % of the overall Gross Domestic Product (GDP) and generates about 80 % of the country's foreign exchange earnings [11]. The productivity of agriculture in Ethiopia is extremely sensitive to the temporal and spatial variations in hydroclimatic factors. This vulnerability results from limited technological advances, insufficient infrastructure and poor agricultural practices, which have resulted in draught and famine in different periods [12].

Ethiopia experiences spatial and temporal variations in its climate due to the movement of the Inter-Tropical Convergence Zone (ITCZ) [[13], [14], [15]]. Changes and fluctuations in hydroclimatic variables have significant impacts on Ethiopia's water resources and food security. Due to variability in the aquatic ecosystem, it is important to evaluate streamflow, temperature, and rainfall to understand how they alter hydrology within watersheds. Changes in rainfall, temperature, and other climatic factors have significant influence on the fluctuations in streamflow levels and the timing of hydrological events [16]. Therefore, it is very important to identify the historical seasonal and interannual variations and conduct trend analysis of hydrological and climatological variables such as rainfall, temperature, and streamflow at local watershed and regional levels [17]. This process helps to know the response of watersheds to hydroclimatic shifts and helps develop more effective strategies to address the risks induced by climate change. Such insights enable improved allocation and management of water resources, which is essential for future sustainable water resource management [18].

Mann-Kendall (MK) test, Spearman's Rho (SR), linear regression analysis, Innovative Trend Analysis (ITA), wavelet analysis and Theil-Sen Approach (TSA) are found in the literature for evaluating the trends, but the Sen Slope estimator and MK trend test are most commonly used for hydroclimatic trend analysis [19]. These non-parametric techniques offer advantages such as handling missing data, requiring few assumptions, and independence from data distribution [20]. However, a major limitation is the influence of autocorrelation on test significance [21]. Various modifications to the MK test aim to mitigate autocorrelation by pre-whitening data, yet recent studies indicate these may not adequately eliminate the influence of long-term dependency on trend significance [19]. Additionally, localized changes in climate and streamflow due to factors like landuse landcover changes and earth atmospheric phenomena may not exhibit prolonged trends, challenging the assumption of unidirectional trends in MK test results [22,23].

Numerous studies globally have examined the variability, trends and shifting points of hydroclimatic variables. Trends in hydroclimatic variables, including precipitation, temperature, evapotranspiration, and streamflow were analyzed using the MK, Sen's slope estimator, and Arc Map's Inverse Distance Weighted Interpolation for temporal, magnitude, and spatial trends respectively in the Kilombero river catchment, watershed [7]. The result showed increasing trends in precipitation, temperature, and river discharges, while potential evapotranspiration exhibited a declining trend. Spatio-temporal variability in hydroclimate over the Upper Yangtze River Basin, China, was analyzed using MK [24]. No significant change in precipitation were observed from 1951 to 2022, while temperature increased significantly across the basin annually and seasonally, except in some eastern stations. Streamflow showed a sharp increase in winter and spring (dry season) and decrease in summer and autumn (rainy season). The changing patterns of hydro-climatic variables in the Aghanashini River watershed, India, were analyzed using the MK and graphical Innovative-Sen (IS) test [25]. Result showed a clear impact changes, with an upward shift in maximum temperature and a downward shift in rainfall and streamflow time series after 2001.

In Ethiopia, many scholars have assessed the variation of hydroclimatic parameters over a wide range of geographical areas and time scales. Trends in climatic and hydrological parameters in the Ajora Woybo watershed, Omo Gibe River Basin, Ethiopia, were analyzed using the MK and Sen's slope tests [26]. Significant decreases in monthly rainfall was observed in February and March, while rainfall and runoff showed a slight increase during the Kiremt season. Minimum, maximum, and mean annual temperatures displayed significant increasing trends annually. Hydroclimatic variability in the Ghba River Subbasin, Ethiopia was analyzed using regression, MK and SR tests, Sen's slope, and correlation analysis [6]. About 73 % of rainfall stations exhibited normal to moderate annual variability. Most rainfall trend showed no change, except for one station with a decreasing trend. Temperature trend were largely stable, with increases observed in three stations. Streamflow trends and change point timings were consistent across stations, all showing a decreasing trend. Trend and climate change in the Gelgel Belese Watershed, Upper Blue Nile Basin, Ethiopia, were analyzed using MK trend test and a time series linear model [27]. The average annual temperature showed an increasing trend with the rate of 0.0067^0^ c/year and 0.005^0^ c/year at Dangila and Chagni stations, respectively. While Pawi station exhibited a non-significant increasing trend. Evapotranspiration showed a significant increasing trend at Dangila (0.68 mm/5 years) and a non-significant increasing trend at Chagni and Pawi stations. Hydroclimatic variability and trends in the Zarima Sub-Basin, Northwestern Ethiopia, were analyzed using the coefficient of variation, standard anomaly index, MK test, and Sen's slope estimator [16]. Rainfall and temperature showed spatial and temporal variability. Annual rainfall exhibited a statistically significant increasing trend in some areas, with an insignificant increase in most part of the sub-basin, reaching up to 300 mm per decade. According to Ref. [16], the Zarima Sub-Basin experienced hydroclimatic variability, significantly affecting livelihoods, agricultural production, and the local economy.

The ITA method, introduced by Sen [[28], [29], [30]], is widely used in studying trends in hydrological and meteorological variables in numerous regions. This approach is simple, intuitive, applicable regardless of distributional assumptions, and has the ability to identify trends within various subcategories. According to Ref. [29], the ITA method has independence from sample size, data distribution, and serial correlation. In contrast, classical tests cannot distinguish the influence of low and high values on the identified trend. The works cited below illustrate the advantage of the ITA method over conventional approaches [[31], [32], [33]]. Furthermore, the ITA approach enables more comprehensive interpretations of trend detection, which provides advantages in detecting hidden variation trends of hydroclimatic data and graphically displaying the trend variation of extreme events, such as “high” and “low” values of precipitation, temperature and streamflow [[34], [35], [36], [37]].

In recent years, this method has found extensive application in various studies around the world. The comparison of MK and ITA methods for the monthly total precipitation in the Middle Black Sea Region, Turkey, revealed similar trends for peak and low values. MK identified increasing trends in Sinop, Ordu, and Tokat, while ITA showed increases in Sinop, Amasya, and Tokat. ITA provides a graphical advantage in trend analysis [38]. A study in Shaanxi province, china, analyzed annual and seasonal rainfall trends since 1950s using ITA, MK, and linear regression methods. Trends were assessed across 14 rainfall stations, with ITA also examining rainfall intensities and seasonal extreme values. Results showed non-uniform trends regionally and seasonally, with significant decreases in annual rainfall in the Wei River Basin and Northern Loess Plateau. The trend strengthened with increasing rainfall intensity. ITA method demonstrated advantages, including graphical results and sub-trend analysis [39]. Trend analysis of precipitation, temperature (1980–2014), and streamflow (1990–2008) in the Jemma sub-basin of the Upper Blue Nile Basin, Ethiopia. The MK, MMK, Sen's slope estimator and ITA methods were used to identify trends. Mean annual temperature showed a significant upward trend (p < 0.005) of 0.029^0^C per year. Annual precipitation and streamflow increased by 1.781mm/year and 0.085 m^3^/s respectively. Based on Sen's slope estimator, but these trends were not statistically significant [34]. Trends in extreme precipitation indices were analyzed in Lay Gayint, Tach Gaynit, and Simada districts on northwestern Ethiopia using gridded data (1981–2018). ITA and MK methods were used to detect trends. ITA results indicated significant increasing trends (p < 0.01) in 90 % of indices in Lay Gaynit and 70 % in Tach Gaynit, while 60 % showed significant downward trends in Simada. The MK trend test identified fewer trends, with 30 % increasing in Lay Gaynit (p < 0.01), 30 % in Tach Gaynit (p < 0.05), and 20 % in Simada (p < 0.05). ITA proved more effective in identifying significant trends [40]. Trend analysis of climatic and hydrological variables in the Awash River Basin, revealed significant variability in precipitation, temperature, and streamflow. Using ITA, MK, and Sen's slope methods, annual precipitation showed significant increases in Fitche (Z = 0.82) and Gewane (Z = 0.8), while slightly decreased in Bui (Z = 0.69) and sharply decreased in Sekoru (Z = 0.45). Temperature trends significantly increased in Fitche (Z = 3.77), Bui (Z = 4.84), and Gewane (Z = 5.59), but decreased significantly in Sekoru (Z = 1.37). streamflow showed a decreasing trend over the study period [41]. A long term (1902–2021) trend analysis of seasonal and annual rainfall in the Rajsamad district of Rajasthan was conducted using the ITA technique. ITA, known for its graphical results, identified trend as ‘low’, ‘medium’, or ‘high’, useful for future flood and draught studies. No significant trend was observed for annual rainfall, while a positive trend was observed in the winter season. Negative trend was observed in pre-monsoon and post-monsoon seasons [42]. Long-term precipitation trends in the Modjo watershed, Central Ethiopia, were analyzed to assess spatiotemporal variability. The study used MK, ITA, and Sen's slope estimator. Rainfall variability ranged from 16 to 59 % annually, 18–63 % in summer, and 50–90 % in spring. Significant trends (p < 0.05) in annual rainfall were observed in 28.6 % of stations using the MK trend test and 42.9 % using ITA, indicating that ITA identifies more significant trends than the MK trend test [43].

Recent advancement in ITA have resulted in the development of the Innovative Triangular Trend Analysis (ITTA) [44]. ITTA is an advanced statistical method used to detect and analyze trends in time series data, particularly in complex environmental datasets such as hydrology and climate data [45]. ITTA offers a sophisticated and flexible approach to trend analysis, especially for complex time series data [46]. Furthermore, Innovative Polygon Trend Analysis (IPTA) is an advanced statistical technique developed as an extension of the ITA method. IPTA builds on ITA by incorporating polygonal segmentation to analyze the time series data, providing a more comprehensive understanding of trends in datasets with complex, non-linear pattern. This method mostly applicable for the hydrological and climate data often exhibit irregularities, seasonality, and abrupt changes [44,[42], [43], [47], [48]]. The ITA method was selected and used for trend analysis due to the absence sophisticated complexities in the hydrological and climatic data of Upper Omo Gibe basin.

Previous studies in the Upper Omo Gibe Basin [49,50] has been mainly used MK method to analyze trends. The study revealed a significant increase in seasonal and annual mean temperatures over the past two decades. While the rainfall during summer and autumn seasons in the northern and central parts of the basin exhibited a statically significant positive and negative trend, the basin experienced notable spatio-temporal variability in seasonal and monthly rainfall. However, relying exclusively on the MK trend method may not fully capture the complexity of the trend, particularly in the areas with diverse climatic and hydrological conditions. While the MK test is useful, it has limitation, especially when the data exhibit serial correlation or when the trends are not immediately apparent. The MMK method addressees these limitations by counting such issues, resulting in more reliable and accurate trend detection [51]. Additionally, combining the ITA method with MMK and Sen's slope method in R-Studio provides a more comprehensive approach to trend analysis. This integration enhances trend detection by incorporating both non-parametric and robust statistical techniques, improving the accuracy reliability of the result. This integrated approach has not been applied in the Upper Omo Gibe Basin, making this study a novel contribution to the filed.

Even though [50] have studied the hydrological aspect at a broader scale of the Omo River Basin without considering the specific conditions [49]. studied hydro-meteorological trends in the Upper Omo gauging stations, but this study has many limitations. For example, there are more than 10 streamflow gauging stations along the major sub-watershed of the Upper Omo Gibe Basin, but he used only six streamflow gauge stations. There are more than 15 rain gauge stations where recorded rainfall data is available up to 2018, but he only used up to 2008. This indicates almost ten years of variations were not considered in this research. The other limitation of this study is that the spatial distribution of rainfall, streamflow, and temperature was not investigated for Annual, Kiremt, Belg, and Bega. Thus, there is a need to understand the temporal and spatial variation of rainfall, temperature, and streamflow in a watershed using a recent data scale since the Upper Omo Gibe Basin is a major tributary of the Gibe-III Dam hydropower project. No previous study combining the ITA, MMK, and Sen's slope methods in R-studio has been published in the Upper Omo-Gibe Basin. The main advantage of using the ITA method is that it does not depend on assumptions such as sample number, non-normality, and serial correlation and trends of low, medium, and high data can be easily observed by using this method [51]. Hence, it's vital to assess the historical annual and seasonal variability and conduct trend analyses of hydroclimatic variables such as rainfall, temperature, and streamflow at both basin and sub-basin scales. This helps depict how sub-watersheds respond to hydroclimatic changes and aids in developing more effective strategies to manage risks induced by climate shifts. This approach enhances the management and distribution of water resources for sustainable practices in the future.

## 2 Materials and methods

### 2.1 Study area description

**Fig. 1:**
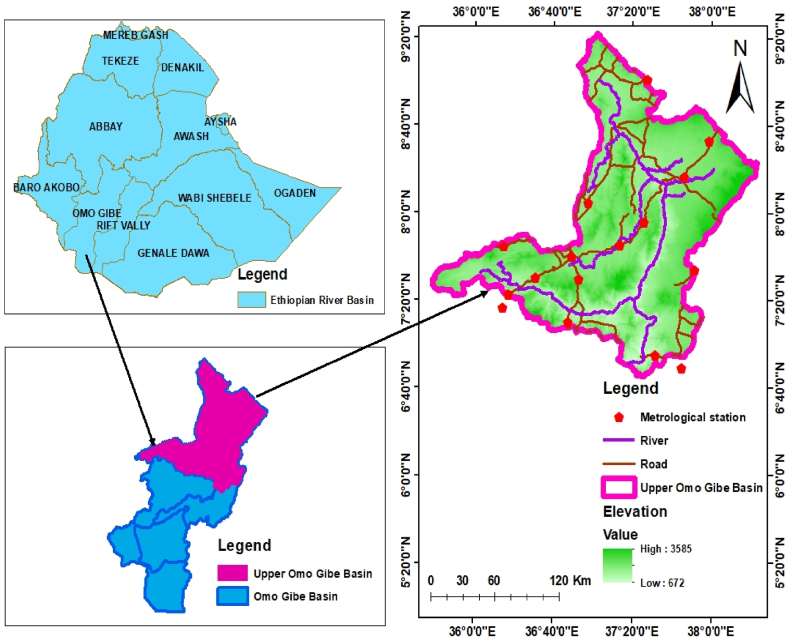
Location map of Upper Omo Gibe Basin.

The Upper Omo Gibe Basin is located in the south-western highlands of Ethiopia. Geographically located at latitude 6^o^ 44′ 58” -9^o^ 22′ 21″ N and longitude 35^o^ 37′ 40″-38^o^ 23′ 15″E with elevation ranges from 672 to 3585 m a.m.s.l as shown in Fig. 1. It is located 400 km from the capital city of Ethiopia-Addis Ababa. The basin covers about 32,866 km^2^ area. The seasonal classification of the Upper Omo Gibe Basin includes Spring (Tseday), Summer (Kiremnt), Autumn (Belg), and Winter (Bega). The Spring (Tseday) season includes the months of March, April, and May. The summer (Kiremnt) season covers the months of June, July, and Augst. Autumn (Belg) includes the months of September, October, and November. The winter (Bega) contains December, January, and February months. The maximum recorded annual rainfall at Limugenet station was 2056.23 mm and the minimum annual rainfall was 1037.04 mm at Gedo station. The basin generally receives an average of 1492.24 mm of annual rainfall. In the autumn season, the basin receives 339.97 mm, in spring 404.33 mm, in summer 642.39 mm, in winter 105.55 mm of rainfall. June, July, August, and September months receive maximum rainfall. The maximum average annual temperature of the basin was 25.95 °C. The basin reaches a maximum average temperature of 25.45 °C in autumn, a maximum average spring temperature of 27.16 °C, a maximum average summer temperature of 23.83 °C, and a maximum average winter temperature of 27.40 °C. The minimum average annual temperature of the basin was 12.98 °C. The minimum average temperature of the basin in autumn was 12.61^o^C, the minimum average temperature in spring was 13.68 °C, the minimum average temperature in summer was 13.22 °C, and the minimum average temperature in winter was 12.4 °C.

### 2.2 Data collection and analysis

Sixteen stations were selected as target stations for rainfall, maximum temperature, and minimum temperature depending on the length of record and proximity to the basin. Daily rainfall, maximum temperature, and minimum temperature were obtained from the National Meteorological Institute of Ethiopia (NMIE) from 1981 to 2022. Streamflow data were collected from the Ministry of Water and Energy of Ethiopia (MWEE). Streamflow data for the Upper Omo Gibe Basin were collected from ten different gauging stations from 1985 to 2018 that contributed to flow in the basin. The basin has limited hydrological and meteorological data, with numerous gaps in the available information. Due to this, it is important to conduct thorough screening and quality checks on all data before using it in any hydro-meteorological analysis. The hydrological and meteorological data were subjected to various quality control procedures, such as checking for missing records, ensuring homogeneity, and identifying inconsistency. The percentage of missing data was computed as the ratio of the total number of days missed and recorded data. The missing data analysis showed 0.85–7.7 % and 4.78–8.99 % for rainfall and streamflow. Multiple Imputation by Chained Equations (MICE) packages in R software were used for filling missed data of rainfall and streamflow. Four homogeneity tests, namely Pettit's, SNHT, Buishad range test, and Von Neumann test (VNT) were conducted to evaluate the homogeneity of data series at various stations. Employing multiple homogeneity tests was crucial to mitigate potential reliability concerns in the data series. The consistency of the data was examined using a double mass curve, which compared data from the station with data from multiple additional stations in the basin.

### 2.3 Methods

Investigating the presence of trends in hydro-meteorological variables is very critical for the present and future water resources development in the Upper Omo Gibe Basin. This study used different methods for examining the variability and trend of rainfall and temperature in sixteen stations and streamflow in ten stations. Variability analysis involves using the Coefficient of Variation (CV) and Standard Anomaly Index (SAI), while trend analysis uses MMK, Sens Slope estimator, and ITA. Data trend analysis was undertaken using R statistical software by “modifiedmk” and “trendchange” packages.

#### 2.3.1 Variability analysis

The variability of rainfall, temperature, and streamflow was determined using the CV and SAI.

##### 2.3.1.1 Coefficient of Variation (CV)

CV is a statistical measure used to represent the relative variability of a data set compared to its mean. Computed by dividing the standard deviations of the data by the mean of the data and then multiplying by 100 to express it as a percentage [[52], [53], [54], [55]] as shown in Equation (1).

(1) $$CV=\left(\frac{\sigma }{\bar{x}}\right)*100$$

Where CV is the coefficient of Variation, σ, and $\bar{X}$ denote standard deviation and long-term mean of rainfall, temperature, and streamflow respectively. A low coefficient of variation shows that the data points are closely clustered around the mean. While a high coefficient of variation suggests that the data points are more spread out relative to the mean. In this study, the degree of variability of hydroclimatic variables was computed by CV, as less (CV < 20 %), moderate (20 < CV < 30 %), high (CV > 30 %), very high (CV > 40 %), and CV > 70 % indicate extremely high inter-annual variability of the hydroclimate data.

##### 2.3.1.2 Standard Anomaly Index (SAI)

SAI is a statistical measure used to measure how much a particular data point deviates from the average value within a dataset [54,56]. It helps to identify the dry and wet years with long-term mean rainfall, temperature, and streamflow and examines the nature of trends [16]. It is computed by subtracting the mean from the individual data point and then dividing by the standard deviation of the dataset [57,58] as shown in Equation (2). This measure helps in identifying the degree of deviation of individual data observation from the average, indicating the relative significance of each observation within the dataset. Positive SAI indicates above the average data, indicating the period is wet. When the value of SAI is negative the value is below the average, indicating the period is dry. SAI values and their category are illustrated in Table 1.

(2) $$SAI=\left(\frac{X-\bar{x}}{\sigma }\right)$$

Where SAI is the standardized anomaly index, x is the seasonal and annual mean of rainfall, temperature, or streamflow of a study year; $\bar{x}$ is the mean annual and seasonal rainfall, temperature, or streamflow throughout observation and σ is the standard deviation of seasonal and annual rainfall, temperature or streamflow throughout observation.

**Table 1:** Standardized anomaly index value interpretation [59].

SAI value	Category
Above 2	Extremely wet
1.5 to 1.99	Very Wet
1 to 1.49	Moderately we
−0.99 to 0.99	Near normal
−1.49 to − 1	Moderately dry
−1.99 to− 1.5	Severely dry
−2 and less	Extremely dry

#### 2.3.2 Trend test

A trend test is a statistical method used to determine if there is a systematic trend or pattern present in a dataset over time or space. The basic idea behind a trend test is to assess whether there is evidence of a consistent increase, decrease, or no trend in the data throughout observation. There are different methods that can be used for trend analysis depending on the nature of the data and specific research objectives. Various tests exist for identifying and estimating trends in hydro-climatic factors. Parametric and non-parametric techniques are both viable for conducting trend detection. One instance of a parametric method is the linear regression test. Non-parametric (also called rank-based) methods include; i) Mann-Kendall test [60,61], ii) Spearman's rho test [62,63], iii) Onyutha test [64], etc. Parametric trend tests like the *t*-test are employed in examining changes in climate variables across time. Yet, their utility with hydro-climate variables is limited due to their reliance on the time series conforming to a normal distribution [65]. Within this research, the non-parametric MMK, ITA, and Sen's slope method were applied for trend analysis.

##### 2.3.2.1 Mann-Kendall

Mann-Kendall trend test is a non-parametric statistical test method used to identify trends in the time series data [66,67]. It is one of the most frequently used non-parametric tests in identifying trends within hydro-meteorological data, which do not require the data points to be normally distributed. It is used for monotonic trend detection. The MK test, being distribution-free, offers reduced sensitivity to outliers [6,68]. Non-parametric methods show greater success in skewed distributions, giving them an edge over parametric analyses for conducting trend tests on hydro-climate variables [17,69]. The equations for computing Mann-Kendall statistics (S) and standardized test statistics Z [61,70] are as in Equation (3).

(3) $$S=\sum_{i=1}^{N-1}\sum_{j=i+1}^{N}sgn\left(X_{j}-X_{i}\right)$$

with n the duration length of the data, X_i_ and X_j_ are the data values in the times series at the time step i and j(j > i), respectively, and sgn(X_j_ − X_i_) is computed by Equation (4) [71].

(4) $$sgn\left(\theta \right)=\left\{\begin{array}{c} 1\;if\;\left(X_{j}-X_{i}\right)>0\\ 0\;if\;\left(X_{j}-X_{i}\right)=0\\ -1\;if\;\left(X_{j}-X_{i}\right)\langle 0 \end{array}\right\}$$

where: assuming (X_j_ - X_i_) = θ and the value sgn(θ) was calculated as the signum functioning, which is the number of data. If the dataset is identically and independently distributed, then the mean of S is zero, and then the variance S is calculated by Equation (5) [72].

(5) $$V\left(S\right)=\frac{1}{18}\left[n\left(n-1\right)\left(2n+5\right)-\sum_{i=1}^{g}t_{i}\left(t_{i}-1\right)\left(2t_{i}+5\right)\right]$$

For the case where the size of the sample data is bigger than 10 (n > 10). Where n represents the length of the data set, g represents number of tied groups (sample data with identical values) in the time series

(6) $$Z_{s}=\left\{\begin{array}{c} \frac{S-1}{\surd var\left(S\right)},S>0\\ 0,S=0\\ \frac{S+1}{\surd var\left(S\right)},S<0 \end{array}\right\}$$

The Zs is the standard statistics test and it is computed by Equation (6) [73,74] The statistical significance level of the trend variation was evaluated using the Zs value. A positive MK statistic (Z > 1.96) indicates a significant increasing trend, whereas a negative (Z < −1.96) indicates a significantly decreasing trend, at the α = 0.05 level of significance.

The linear regression model is used to measure the pattern or trend of variables over a long period. It is calculated by Equation (7) [75].

(7) $$Y=a+bx_{t}$$

where: Y indicates the trend value, a is the intercept, b is the slope of the trend, and X_t_ is the time point. Pearson correlation coefficient (r) was used to evaluate the goodness of fit and linear association of rainfall, temperature, and stream flow data.

##### 2.3.2.2 Sen's slope estimator

Sen's slope estimator serves to estimate the trend's magnitude, functioning as a non-parametric method capable of determining the change per unit time [76]. However, it operates under the assumption of a linear trend within the time series. The slope (T_i_) of all pairs of data x can be estimated as Equation (8) [77].

(8) $$T_{i}=\frac{x_{k}-x_{j}}{k-j},j\neq k$$

where: x_j_ and x_k_ are the data values of time j and k, respectively. If there are n values (xj) in the time series, there will be N = n(n-1)/2. Thus, Sen's slope estimator is defined as the median slope of the N values of Ti. The overall slope estimator Qi is calculated either by Equation (9) or 10 [78].

If N is an odd observation

(9) $$Q_{i}=\frac{T_{N+1}}{2}$$

Or

(10) $$\text{If}\;N\;\text{is}\;\text{an}\;\text{even}\;\;\text{observation}:Q_{i}=\frac{1}{2}\left(\frac{T_{N}}{2}+\frac{T_{N+2}}{2}\right)$$

##### 2.3.2.3 Modified Mann–Kendall (MMK) test

Mann-Kendall and Sen's slope estimator estimates the trends under the assumption that the time series data is independent, serially non-correlated, and randomly arranged [66,79]. However, in the data series, there could be a significant autocorrelation, due to this a significant challenge that leads to uncertainties and errors in trend detection. The Modified Mann-Kendall (MMK) test was applied for serially correlated data with a significant lag-1 autocorrelation coefficient, employing the variance correction method [80].

The MMK removes the significant autocorrelation from the time series data by using pre-whitening techniques [[81], [82], [83], [84]]. This involves the analysis of lag-1 autocorrelation using Equation (11) [84]. If the value of $r_{i}$ lies between the boundary limit of Equation (12) [84] at a 10 % significance level, then the time series data is serially independent so in this case, it is possible to apply Mann- Kendall methods for trend analysis. If the values of $r_{i}$ falls outside of the upper and lower margin, the trend is shifted from the main time series data using Equation (13) [84]. Lag-1 autocorrelation coefficient is computed by Equation (11) [84]. The lag-1 autoregressive component is removed from the time data series by using Equation (14) [84]. Finally, the correct series is developed in Equation (15) [84].

(11) $$r_{i}=\frac{\frac{1}{n-k}\sum_{i=1}^{n-k}\left(y_{i}-\bar{y}\right)\left({y_{i}}_{+k}-\bar{y}\right)}{{\sum_{i=1}^{n}\left(y_{i}-\bar{y}\right)}^{2}}$$

(12) $$\frac{-1-1.645\sqrt{n-2}}{n-2}\leq r_{i}\leq \frac{-1+1.645\sqrt{n-2}}{n-2}$$

(13) $$y^{\prime }=y_{i}-\left(\beta *i\right)$$

(14) $$y^{\prime \prime }=y^{\prime }i-ri*y_{i-1}^{\prime }$$

(15) $$y^{\prime \prime \prime }=y^{\prime \prime }+\left(\beta *i\right)$$

Where $y^{\prime }$ is the time series data at a time (i), $\bar{y}$ average values of the data, β is the Sen's slope values of the data, $y^{\prime }$ is the detrended time series data, $y^{\prime \prime }$ is the time series after the removal of auto-correlation from the detrended time series data, and $y^{\prime \prime \prime }$ is the new blended time series. Depending on the auto-correlation analysis. For those stations that showed significant auto-correlations, MMK was used for the analysis of trends.

##### 2.3.2.4 Innovative Trend Analysis methods (ITA)

The ITA method has been used in numerous studies to identify hydro-meteorological observations and its accuracy has been assessed by comparing it with the outcomes of the MK method [21,41]. Within the ITA method, hydro-meteorological observations were categorized into two classes, and subsequently, the data points were organized separately in ascending order. Following that, the two classified were positioned on a coordinate system with (Xi: i = 1, 2, 3, 4 … n/2) along the X-axis and (Xj: j = n/2 + 1, n/2 + 2, n/2 + 3 ….n) along the Y-axis. When the time series data on a scatter plot align along the 1:1 (45^o^) straight line, it signifies the absence of a trend. Conversely, an increasing trend is maintained when data points cluster above the 1:1 straight line, while a decreasing trend is observed when data points is below the 1:1 straight line.

## 3 Results and discussion

### 3.1 Spatio-temporal distribution of the annual and seasonal rainfall

**Fig. 2:**
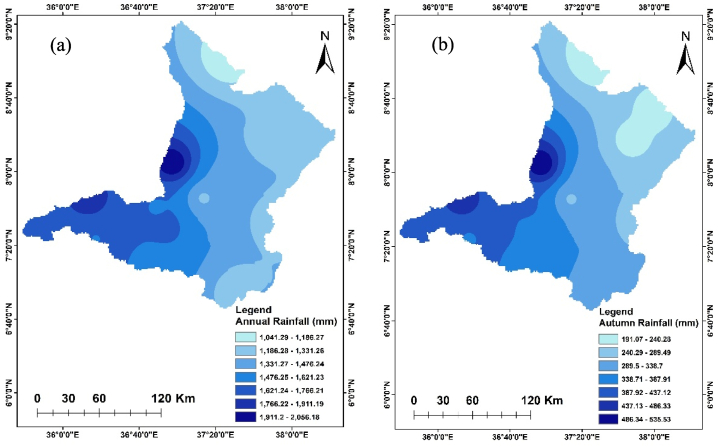

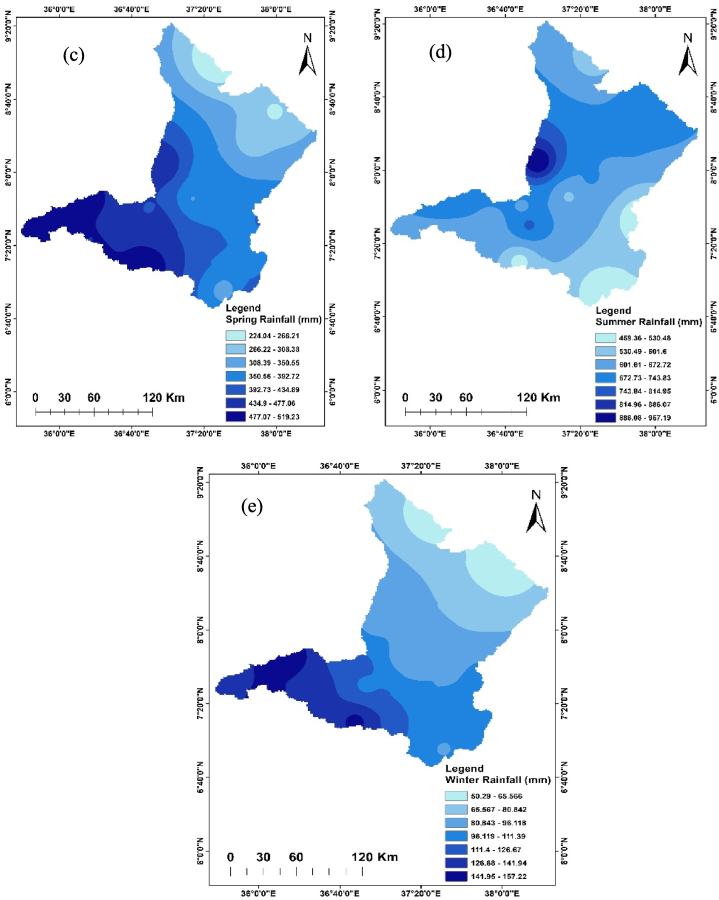
Annual and seasonal distribution of rainfall (a) Annual (b) Autumn (c) Spring (d) Summer (e)Winter.

The annual rainfall of the Upper Omo Gibe Basin from 1981 to 2022 was spatially distributed ranging between 1037.04 mm and 2056.23 mm. The western, central, and southern parts of the basin received the highest amount of rainfall, while the northern and eastern parts of the basin received the least amount of rainfall. During the autumn season, the highest rainfall ranges from 191.07 mm to 240.28 mm observed in the eastern part of the basin and the lowest rainfall ranges from 486.32 mm to 535.53 mm observed in the western part of the basin. During the spring season, the rainfall ranges from 222.66 mm to 519.23 mm. The lowest rainfall in this season ranges from 224.03 mm to 266.20 mm in the northern part of the basin and the highest rainfall ranges from 477.06 mm to 519.23 mm in south western part of the basin. During the summer season, the highest rainfall amount ranging from 886.06 mm to 957.18 mm was recorded in the western part of the basin while the lowest rainfall ranging from 459.36 mm to 530.48 mm was recorded in the south-eastern part of the basin. The driest season of the basin is winter. In this season the maximum rainfall ranges from 141.94 mm to 157.22 mm in the south-western part of the basin and the lowest rainfall ranges from 50.29 mm to 65.56 mm in the northern part of the basin. Significant spatial and temporal distribution of rainfall have existed in the Upper Omo Gibe Basin. This finding is consistent with studies conducted in various regions of Ethiopia. The country's rainfall patterns show significant variation in both space and time, largely due to the diverse topography of the Geshy watershed in south-western Ethiopia [85]; Alwero watershed, western Ethiopia [86]; Tekeze Atbara River Basin, north-west Ethiopia [87]; Gilgel Gibe watershed, south-west Ethiopia [88]; Sidama Regional State, Ethiopia [89]. Fig. 2 shows the seasonal and annual distribution of rainfall in the Upper Omo Gibe Basin. The amount and spatial distribution of rainfall tend to play the most crucial role in the year-to-year variations in crop yields in Ethiopia. These aspects have a profound impact on the country's economy and food supply.

### 3.2 Spatio-temporal variability and trend analysis of rainfall

#### 3.2.1 Spatio-temporal variability of the rainfall

**Fig. 3:**
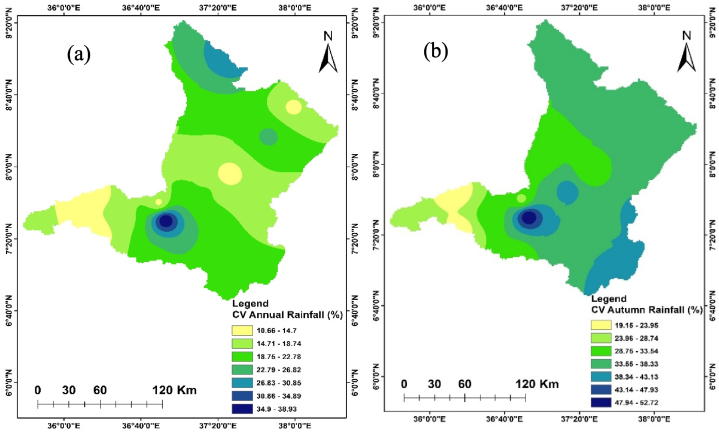

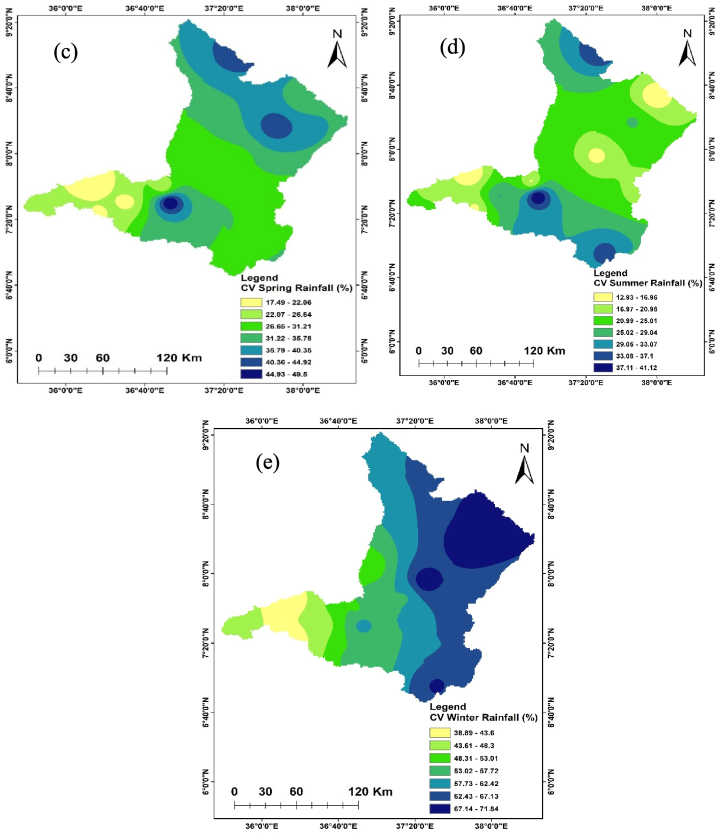
Coefficient of variation annual and seasonal rainfall (a) Annual (b) Autumn (c) Spring (d) Summer (e) Winter.

**Fig. 4:**
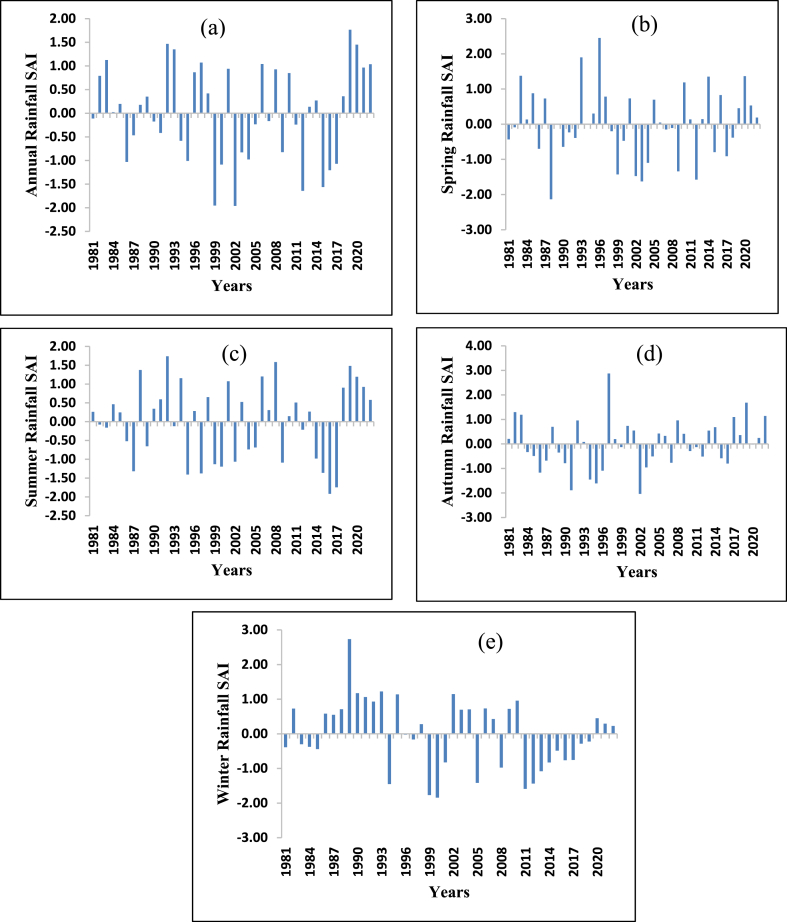
Standard anomaly index of annual and seasonal rainfall (a) Annual (b) Spring (c) Summer (d) Autumn (e) Winter.

The seasonal and annual variability of the rainfall in the Upper Omo Gibe Basin was calculated by CV and SAI. Fig. 3, Fig. 4 represent the CV and SAI values, respectively. The CV of mean annual rainfall was 10.66–38.92 % ranging from less to moderate. The CV of the Autumn season was 19.15–52.72 % ranging from low to very high. The CV of the spring season was 17.49–49.49 % ranging from low to very high. The CV of the summer season was 12.92–41.12 % ranging from low to very high. The CV of the winter season was 38.89–71.83 % ranging from high to extremely high variability. The annual rainfall was less variable than the seasonal rainfall. Less variability of rainfall in spatial and temporal was observed in the summer season and annual time scale. Conversely, significant variability in rainfall was observed during the winter and autumn seasons. Similar results were obtained by Ref. [90] in north-west Ethiopia [91], in the northern highlands of Ethiopia, and [92] in the west Harerge zone, Ethiopia received that seasonal rainfall is less in the summer season and annual time scale.

By considering the annual rainfall pattern of the study period, the percentage of positive and negative anomalies was 47.62 % and 52.38 % respectively. The SAI result showed that 9.52 % of the study period was classified as severely dry, covering the years 1999, 2002, 2012, and 2015. 11.9 % of the study period was categorized as moderately dry, which contains the years 1986, 1995, 2000, 2016, and 2017. The year 2009 falls into the very wet category. The years 1983, 1992, 1993, 1995, 1997, 2006, and 2019 which account for 16.667 % of the study period, were categorized under moderately wet conditions. 54.75 % of the study period was categorized under normal conditions. These coincide with Ethiopia's historical dry years such as 1982, 1984/85, 1990–1992, 1994, 1997, 1999, 2003/2004, 2005, 2008/2009, 2011 and 2016/2017 [93,94].

Moreover, Fig. 4 shows the seasonal and annual SAI of rainfall in the study area. Like the annual rainfall patterns, the seasonal variability was observed in spring, summer, autumn, and winter seasons with negative anomalies of 50 %, 45.23 %, 50 %, and 50 % respectively. Additionally, Table 2 illustrates the seasonal and annual intensity as well as the frequency of rainfall in the study area. The highest positive anomaly (extreme wet) was observed in 1996 (2.38 %), 1997 (2.38 %), and 1989 (2.38 %) during spring, autumn, and winter respectively. Sever wet was observed in 1992 (2.3 %) during spring, 1993 and 2008 (4.76 %) during summer, and 2019 (2.38 %) during autumn. An extreme dry period was observed in 1988 (2.38 %) during spring and in 2002 (2.38 %) during autumn. A severe dry period was observed in 2003 and 2012 (4.76 %) during spring, in 2016 and 2017 (4.76 %), in 1991 and 1995 (4.76 %) during autumn, and in 1999, 2000 and 2011 (7.14 %) during winter season. The finding of this study was supported by Refs. [93,95].

**Table 2:** Annual and seasonal rainfall standardized anomaly index category frequency and percentage.

Standardized anomaly index	Annual		Spring		Summer		Autumn		Winter	
	Frequency	Percentage	Frequency	Percentage	Frequency	Percentage	Frequency	Percentage	Frequency	Percentage
Extreme wet	–	–	1	2.38	–	–	1	2.38	1	2.38
Severe wet	1	2.38	1	2.38	2	4.76	1	2.38	–	–
Moderate wet	7	16.67	4	9.52	6	14.28	4	9.52	5	11.90
Normal	25	59.52	29	69.04	24	57.14	30	71.42	29	69.04
Moderately dry	5	11.90	4	9.52	8	19.04	3	7.14	4	9.52
Severe dry	4	9.52	2	4.76	2	4.76	2	4.76	3	7.14
Extreme dry	–	–	1	2.38	–	–	1	2.38	–	–
Total	42	100	42	100	42	100	42	100	42	100

#### 3.2.2 Trend analysis of the annual and seasonal rainfall

##### 3.2.2.1 Modified Mann-Kendall method

**Fig. 5:**
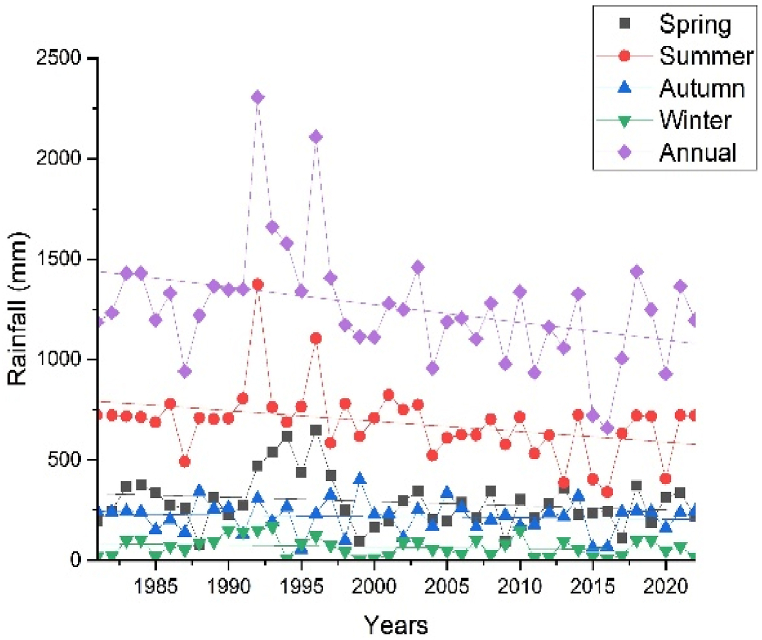
Figure annual and seasonal rainfall trend of Wolkite station.

Trend analysis of the Upper Omo Gibe Basin using the MMK method is shown in Table 3. The time series plot for annual and seasonal rainfall stations is shown in Fig. 5 (sample for Wolkite station). The direction and magnitude of the trend in both annual and seasonal varied across the Upper Omo Gibe Basin. The annual and seasonal rainfall of the basin exhibits both a statistically significant (P ≤ 0.05) and a statistically insignificant trend. According to this method in annual rainfall, a statistically significant increasing trend was observed in Assendabo (Z = 3.641, S = 11.046), Limugenet (Z = 3.641, S = 16.484) and Jimma (Z = 2.428, S = 7.539) stations whereas statistically significant decreasing trend was observed in Dedo (Z = −2.732, S = −31.099) and Deri Goma (Z = −3.121, S = −6.040) stations. In the spring season, a statistically significant increasing trend was observed in Assendabo (Z = 2.492, S = 3.471) and Limugenet (Z = 2.062, S = 4.358) stations whereas a statistically significant decreasing trend was observed in Dedo (Z = −5.464, S = −11.033) station. In the summer season, a statistically significant increasing trend was observed in Jimma (Z = 2.298, S = 3.412) and Limugenet (Z = 2.260, S = 6.171) stations whereas a statistically significant decreasing trend was observed in Deri Goma (Z = −2.924, S = −4.168) and Wolkite (Z = −2.189, S = −2.867) stations. In the Autumn season, Bonga (Z = 2.146, S = 2.859), Gedo (Z = 2.016, S = 2.070), and Limugenet (Z = 3.316, S = 6.421) stations showed a statistically significant increasing trend, and Dedo (Z = −3.099, S = −7.500) station were show statistically significant decreasing trend. In the winter season statistically significant decreasing trend was observed in Deri Goma (Z = −2.774, S = −1.347) and Shebe (Z = −4.492, S = −1.676) stations and there are no observations of a statistically significant increasing trend in this season. Insignificant trends were dominant both in annual and seasonal rainfall data. This output agrees with the previous study on the Omo Gibe Basin [71]. During the spring season insignificant increase trend was observed in Bonga, Chida, Chira, Gedo, Jimma, Sekoru, Shebe, and Wolayita stations whereas an insignificant decrease trend was observed in Bele, Deri Goma, Hosaina, Woliso, and Wolkite stations. During the summer season Assenadabo, Bonga, Chida, Gedo, Hosaina, and Shebe stations showed an insignificant increase trend, and an insignificant decreasing trend was observed in Bele, Chira, Dedo, Sekoru, Wolayita, and Woliso stations. In the Autumn season, insignificant increasing trends were observed in Assendabo, Bele, Chida, Chira, Hosaina, Jimma, Sekoru, Shebe, Wolayita, and Woliso stations whereas insignificant decreasing trends were observed in Deri Goma and Wolkite stations. During winter insignificant increases were observed in Assendabo and Jimma stations whereas an insignificant decreasing trend was observed in Bele, Bonga, Chida, Chira, Dedo, Gedo, Hosaina, Limugenet, Sekoru, Wolayita, Woliso, and Wolkite stations. In the Annual station, insignificant increasing trends were observed in Bele, Bonga, Chida, Gedo, Jimma, and Wolayita stations whereas insignificant decreasing trend was observed in Chira, Hosaina, Sekoru, Shebe, Woliso, and Wolkite stations. Based on the Senʼs slope estimator the annual rainfall significantly increasing trend magnitudes up to 338.94 mm/decade and decreasing trend ranges up to 525.54 mm/decade. A maximum decreasing trend was observed in the winter season and a maximum increasing trend was observed in the autumn season. Generally annual (S = −0.21031), winter (S = −0.80569), and summer (S = −0.113) showed a decreasing trend whereas spring (S = 0.08825) and autumn (S = 1.02075) showed an increasing trend. The same result was obtained in north-central Ethiopia's Woleka sub-basin where summer and annual rainfall have increased trend [54]. The rainfall of the Upper Omo Gibe Basin showed an average reduction rate of 5.8686 mm/decade throughout the study period. The same result was obtained in the Upper Awash Basin by Ref. [96] and in the Gilgel Gibe watershed by Ref. [88]. According to different reports, the amount of rainfall in sub-Saharan countries decreased and this affects the agricultural activity of developing countries like Ethiopia where agriculture is primarily dependent on rainfall [[97], [98], [99]]. All station's annual and seasonal rainfall trend graphs were included in the supplementary material.

**Table 3:** Modified Mann-Kendall (Z) and Senʼs slope (S) values of annual and seasonal rainfall.

Station	Spring		Summer		Autumn		Winter		Annual	
	Z(MMK)	Sen	Z(MMK)	Sen	Z(MMK)	Sen	Z(MMK)	Sen	Z(MMK)	Sen
Assendabo	2.492	3.471	1.757	3.550	2.644	4.148	0.650	0.368	3.641	11.046
Bele	−0.325	−0.367	−0.400	−0.300	1.170	1.114	−1.413	−0.968	0.460	0.936
Bonga	1.279	2.131	1.409	2.778	2.146	2.859	−0.748	−0.511	1.929	6.636
Chida	0.639	1.258	0.108	0.313	0.823	1.000	−1.636	−1.761	0.000	0.056
Chira	0.499	0.495	−0.845	−1.275	0.542	0.738	−1.455	−0.984	−0.108	−0.226
Dedo	−5.464	−11.033	−1.568	−11.924	−3.099	−7.500	−1.461	−0.961	−2.732	−31.099
Deri Goma	−0.607	−0.635	−2.924	−4.168	−0.223	−0.188	−2.774	−1.347	−3.121	−6.040
Gedo	0.000	0.005	0.745	2.500	2.016	2.070	−0.538	−0.124	0.555	4.198
Hosaina	−0.568	−0.961	1.235	1.718	0.339	0.432	−1.864	−1.467	−1.539	−2.823
Jimma	1.691	2.073	2.298	3.412	1.799	2.343	0.461	0.344	2.428	7.539
Limugenet	2.062	4.358	2.260	6.171	3.316	6.421	−0.759	−0.426	3.641	16.484
Sekoru	0.954	1.348	−0.845	−1.200	0.520	0.784	−1.680	−0.945	−0.087	−0.270
Shebe	0.477	0.713	0.058	0.178	0.393	0.464	−4.492	−1.676	−0.720	−1.563
Wolayita	0.368	0.662	−0.065	−0.158	0.607	1.188	−1.552	−1.061	0.022	0.188
Woliso	−0.509	−0.716	−0.360	−0.536	0.932	0.947	−2.010	−0.727	−0.531	−1.247
Wolkite	−0.900	−1.390	−2.189	−2.867	−2.253	−0.488	−1.214	−0.645	−2.449	−7.180

##### 3.2.2.2 Using ITA method

**Fig. 6:**
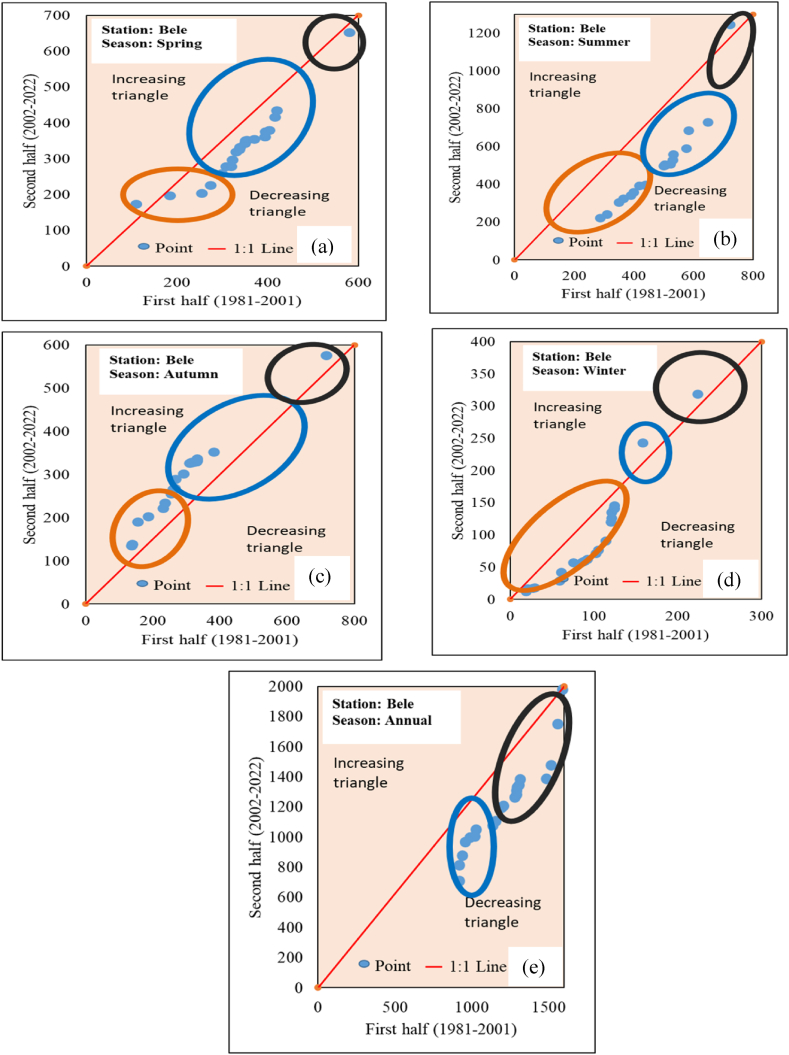
Annual and seasonal rainfall ITA graphical result of Bele station (a) Spring (b) Summer (c) Autumn (d) Winter (e) Annual.

The results of trend analysis using the ITA method for annual and seasonal rainfall data are illustrated in Table 4 and Fig. 6 (sample for Bele station). To detect trends in seasonal and annual, the ITA method was applied to the 16 stations for rainfall and temperature. Both statistical and graphical innovative trend analyses were observed for all 16 stations of rainfall and temperature. The trend observation was achieved through the R “trendchange” package. During the spring season decreasing trends were observed in 9 stations Bele, Dedo, Deri Goma, Gedo, Hosaina, Sekoru, Shebe, Woliso, and Wolkite. Assendabo, Bonga, Chida, Chira, Jimma, Limugenet, and Wolayita stations showed an increasing trend. During the summer season Wolkite, Woliso, Sekoru, Deri Goma, Dedo, and Chira showed decreasing trend. Whereas Assendabo, Bele, Bonga, Chida, Gedo, Hosaina, Jimma, Limugenet, Shebe, and Wolayita stations showed an increasing trend. In the Autumn season Dedo, Hosaina, Sekoru, Shebe, and Wolkite stations showed a decreasing trend whereas Assendabo, Bele, Bonga, Chida, Chira, Deri Goma, Gedo, Jimma, Limugenet, Wolayita and Woliso stations were showed an increasing trend. During the winter season, only Assendabo and Jimma stations showed an increasing trend whereas the rest 14 stations showed a decreasing trend. In Annual rainfall observation Chira, Dedo, Deri Goma, Hosaina, Sekoru, Shebe, Woliso, and Wolkite stations showed a decreasing trend whereas Assendabo, Bele, Bonga, Chida, Gedo, Jimma, Limugenet, and Wolayita stations were showed an increasing trend. Similar seasonal and annual rainfall trends were observed in different watersheds of Ethiopia [33,34,100,101,102].

**Table 4:** Innovative trend analysis trend indicator of seasonal and annual rainfall.

Station	Spring	Summer	Autumn	Winter	Annual
Assendabo	1.681	0.853	1.251	1.123	1.172
Bele	−0.270	0.306	0.150	−0.040	0.079
Bonga	0.815	1.719	2.023	−0.810	1.275
Chida	0.249	0.251	1.324	−2.817	0.145
Chira	0.089	−0.076	0.266	−1.308	−0.059
Dedo	−4.476	−3.284	−4.111	−1.395	−3.699
Deri Goma	−1.071	−1.615	0.237	−1.645	−1.057
Gedo	−0.217	1.923	1.826	−0.138	1.297
Hosaina	−0.667	0.801	−0.704	−2.107	−0.280
Jimma	0.491	0.792	0.349	0.575	0.585
Limu Genet	1.406	2.094	2.237	−0.328	1.852
Sekoru	−0.148	−0.355	−0.173	−1.575	−0.344
Shebe	−0.465	0.184	−0.059	−2.029	−0.248
Wolayita	0.419	0.241	0.605	−1.100	0.254
Woliso	−0.796	−0.069	0.023	−1.873	−0.283
Wolkite	−2.068	−1.971	−0.924	−1.999	−1.823

ITA plots are categorized into three clusters “low”, “medium” and “high” to analyze the trend variations across the study period. The red circle represents “low”, blue represents “medium” and black represent the “high” values. Dataset points of spring season rainfall decreasing trend in “low” categories were observed in Bele, Dedo, Deri Goma, Sekoru, Wolayita, and Woliso stations whereas increasing trends were observed in Chida, Gedo, Hosaina, and Wolkite stations. The dataset points of the spring season rainfall decreasing trend in “medium” categories was observed in Bele, Jimma, Limugenet, and Woliso stations whereas increasing trends were observed in Assendabo, Bonga, Dedo, Gedo, Deri Goma, and Hosaina stations. “High” categories dataset point decreasing trend was observed in Bele, Deri Goma, Jimma, and Limugenet stations. In the summer season, “low” categories decreasing trend were exhibited in the Bele, Gedo, and Shebe stations whereas an increasing trend was exhibited in Chida and Wolayita stations. “Medium” phase decreasing trend was exhibited in Assendabo, Bele, Bonga, Chira, Jimma, Limugenet, Shebe, and Woliso stations whereas an increasing trend was exhibited in Hosiana and Wolayita stations. “high” categories decreasing trend was exhibited in Assendabo, Bonga, Chida, Dedo and Deri Goma stations whereas increasing trend exhibited in Bele, Gedo, Hosaina, Shebe and Wolkite stations. In the autumn season, a “low” categories decreasing trend was exhibited in Dedo, Wolkite, and Shebe stations whereas an increasing trend was exhibited in Assendabo, Bele, Chida, Gedo, and Hosaina stations. “Medium” categories decreasing trend was exhibited in Bonga, Chida, Deri Goma, Limugenet, Shebe, Wolayita, Woliso, and Wolkite stations whereas an increasing trend was exhibited in Assendabo, Bele, Gedo, Dedo, Hosaina, Jimma and Sekoru. “high” categories decreasing trend were observed in Bonga, Chida, Deri Goma, Woliso and Wolkite stations whereas increasing trend were exhibited in Dedo, Hosaina and Jimma stations. In the winter season, the “low” categories dataset of Assendabo, Bele, Chida, Dedo, Hosiana, and Shebe stations exhibited a decreasing trend whereas Chira, Jimma, and Sekoru stations showed an increasing trend. From the “medium” categories dataset decreasing trend was observed in Bonga, Deri Goma, Gedo, Hosaina, Limugenet, Shebe, and Wolkite stations whereas an increasing trend was exhibited in Assendabo, Bele, Chida, Chira, Jimma, Sekoru and Woliso stations. “High” categories decreasing trend was observed in Chira, Dedo, Deri Goma, Jimma, Sekoru, Shebe, and Wolkite stations whereas an increasing trend was observed in Bele, Bonga, Hosaina, and Woliso stations. In annual rainfall observation under “low” categories Chida and Wolayita stations showed an increasing trend whereas Dedo and Gedo stations showed a decreasing trend. In the “medium” categories decreasing rainfall trends were observed in Bele, Chida, Dedo, Gedo, Jimma, Sekoru, and Shebe stations, and increasing trends were exhibited in Hosaina, Wolkite, and Assendabo stations. In “high” categories decreasing trend of rainfall was observed in the Assendabo, Bele, Chida, Dedo, and Shebe stations. All station's annual and seasonal rainfall ITA trend graphs were included in the supplementary material.

### 3.3 Spatio-temporal distribution of annual and seasonal temperature

**Fig. 7:**
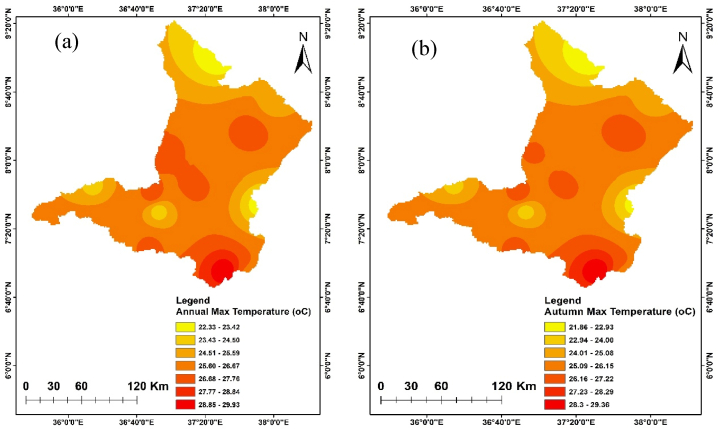

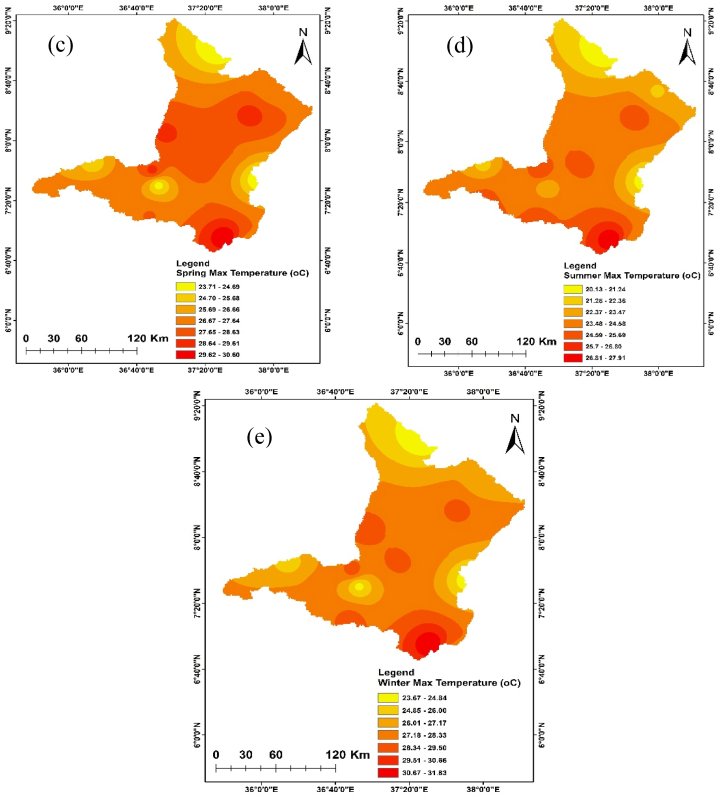
Annual and seasonal distribution of maximum temperature (a) Annual (b) Autumn (c) Spring (d) Summer (e) Winter.

**Fig. 8:**
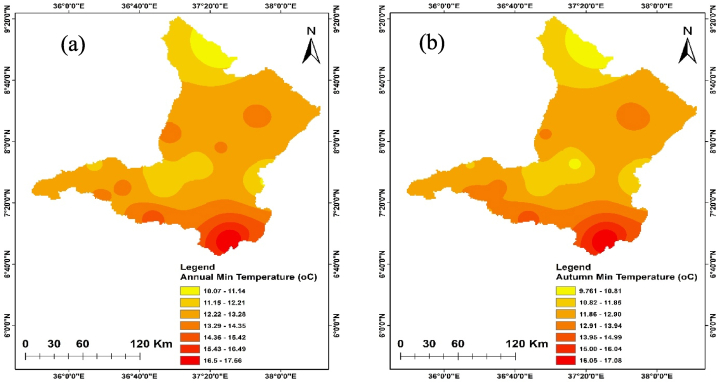

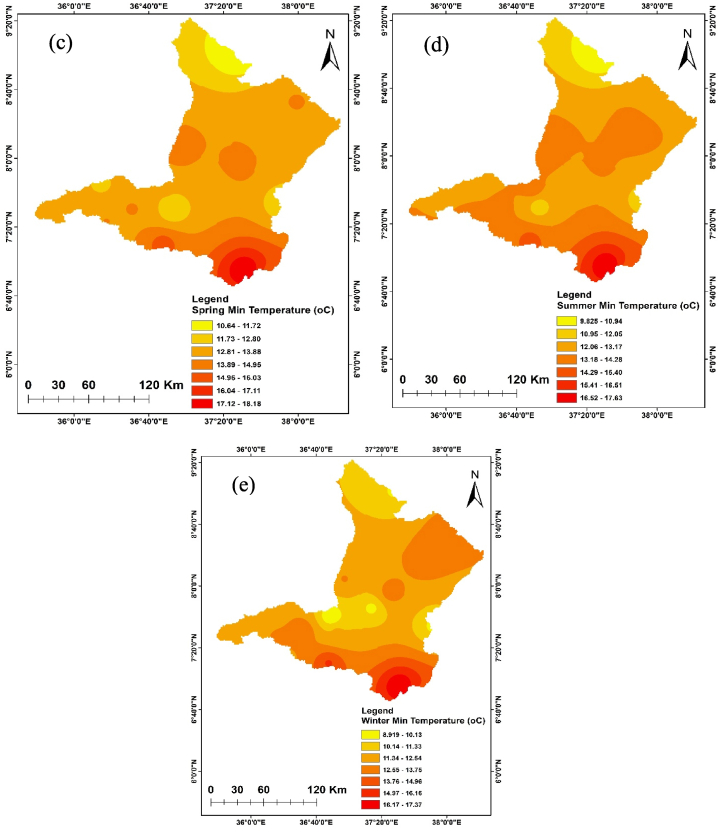
Annual and seasonal distribution of minimum temperature (a) Annual (b) Autumn (c) Spring (d) Summer (e) Winter.

The annual and seasonal maximum and minimum temperatures of the Upper Omo Gibe Basin are given in Fig. 7, Fig. 8. The average maximum temperature of the Upper Omo Gibe Basin ranges from 20.1 °C to 31.83 °C whereas the average minimum temperature ranges from 8.92 °C to 18.19 °C. Annual maximum and minimum temperature ranges between 22.3 °C to 29.93 °C and 17.56 °C to 10.04 °C respectively. The highest maximum temperature 27.75 ^0^C-29.92 °C is shown in the south-west and central-western parts of the basin. The lowest annual maximum temperature 23.41 ^0^C-22.33 °C is shown in the northern and some eastern parts of the basin. Seasonally, the autumn season maximum temperature ranges from 21.86 ^0^C-29.36 °C and the minimum temperature ranges from 9.76 ^0^C-17.08 °C. In this season the maximum temperature is shown in the southern and the lowest temperature shown northern part of the basin. The spring season maximum temperature ranges from 23.70 ^0^C-30.59 °C. The maximum temperature is shown in the southern and central parts of the basin. The minimum temperature ranges from 10.64 ^0^C-18.18 °C and the lowest temperature is shown in the northern and south-western parts of the basin. In the summer season, the maximum temperature ranges from 20.13 ^0^C-27.91 °C, and the minimum temperature ranges from 9.82 ^0^C-17.62 °C. In this season the maximum temperature is shown in the southern and central parts of the basin whereas the minimum temperature is shown in the northern part of the basin. The winter season maximum temperature ranges from 23.67 ^0^C-31.82 °C and the minimum temperature ranges from 8.91 ^0^C-17.37 °C. The maximum temperature is shown in the central and southern parts of the basin and the minimum temperature is shown in the western and northern parts of the basin. Seasonally the maximum temperature of 31.82 °C was recorded in the winter season whereas the minimum temperature of 9.82 °C was recorded in the summer season. The winter season was hotter than other seasons this is because this season contain the driest months of Ethiopia (December, January, and February). Generally, the annual and seasonal maximum and minimum temperatures showed that the southern and central parts of the basin were the hottest, whereas the northern, eastern, and some western-central parts of the basin were the coldest. The coldest part of the basin was the highland parts, whereas the hottest part of the basin was the lowland parts. Similarly [103], observed the highest temperature in winter seasons in the Upper Genale River Basin, Ethiopia [86]; in the Alwero watershed, western Ethiopia [104]; Ziway Lake Basin, Ethiopia [85]; in the Geshy watershed, south-west Ethiopia [105]; in Suha watershed, Upper Blue Nile Basin, north-west Ethiopia.

### 3.4 Spatio-temporal variability and trend analysis of annual and seasonal temperature

#### 3.4.1 Spatio-temporal variability of the annual and seasonal temperature

**Fig. 9:**
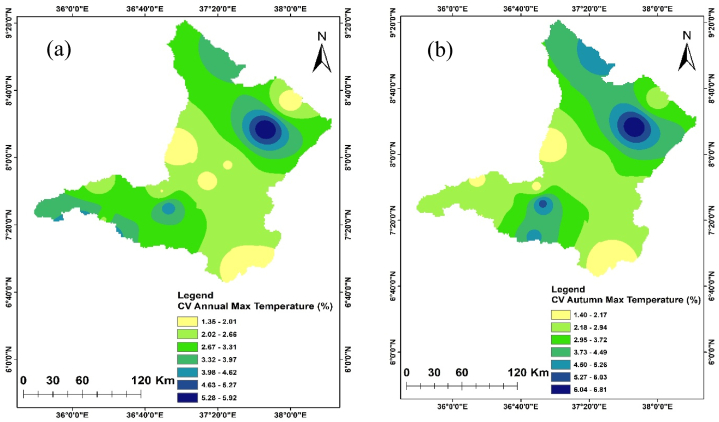

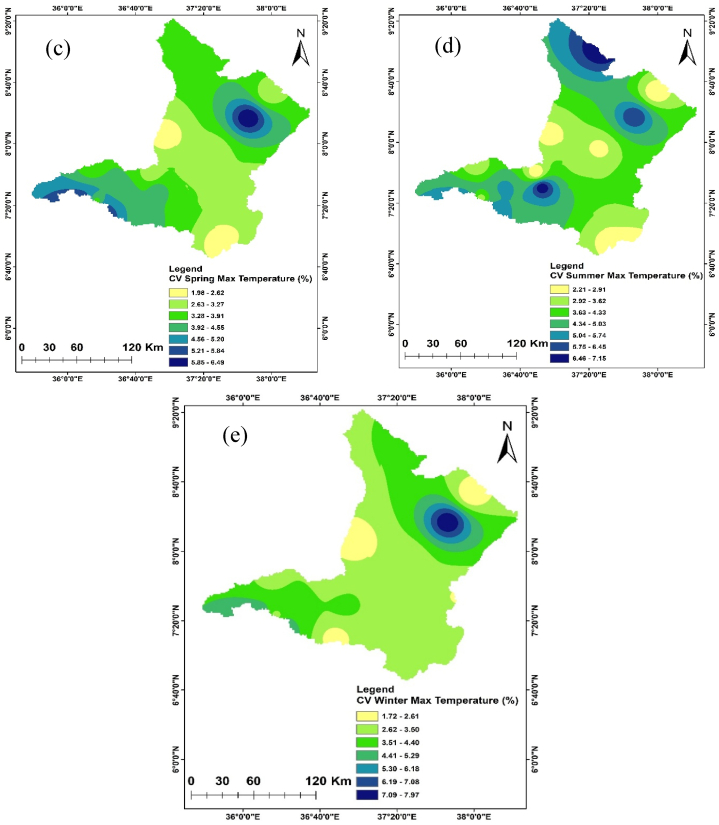
Coefficient of variation of annual and seasonal maximum temperature (a) Annual (b) Autumn (c) Spring (d) Summer (e) Winter.

**Fig. 10:**
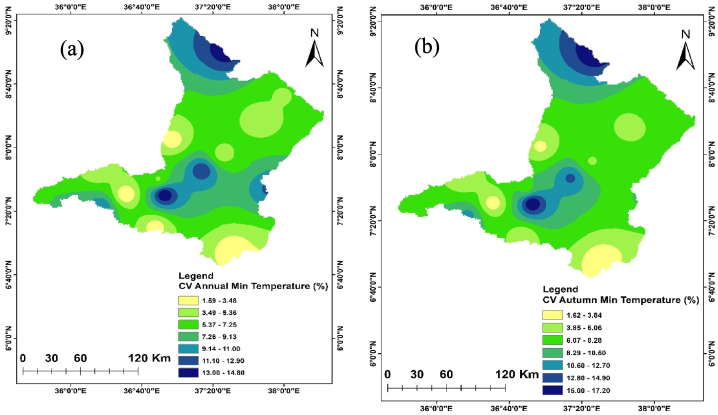

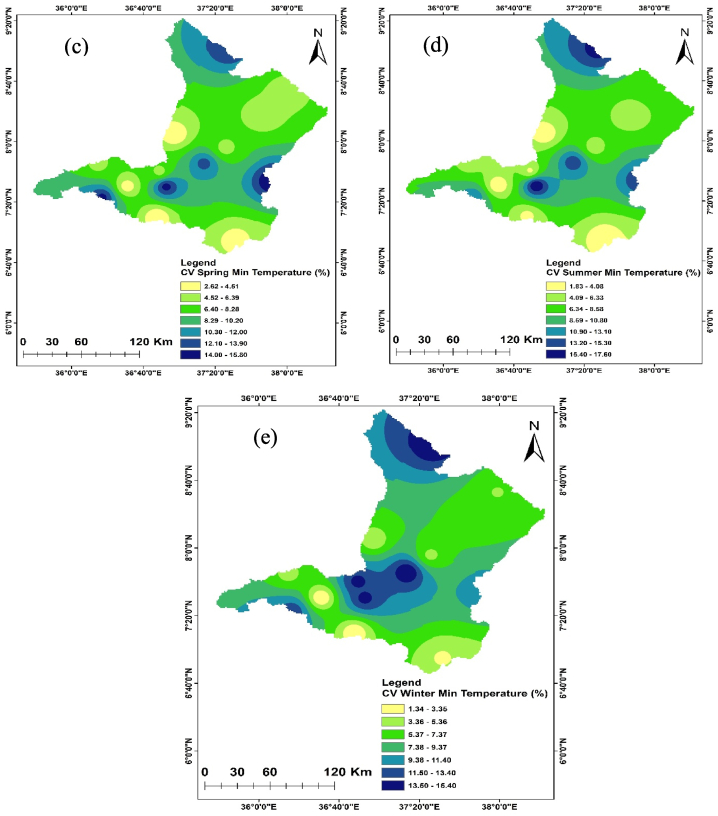
Coefficient of variation of annual and seasonal minimum temperature (a) Annual (b) Autumn (c) Spring (d) Summer (e) Winter.

Temperature is crucial in determining the amount of water lost across a basin. As the temperature increases, soil moisture decreases through evapotranspiration, which in turn affects the amount of runoff because of the resulting drier soil conditions [[106], [107], [108]]. The spatiotemporal variations of the maximum and minimum temperature of the Upper Omo Gibe Basin were presented in Fig. 9, Fig. 10 respectively. The CV of the minimum temperature was larger than the maximum temperature and the CV of the seasonal temperature data was greater than annual observations. Similar observations were obtained by Ref. [16] in the Zarima Sub-basin, north-western Ethiopia; and [1] in the Bilate watershed, Ethiopia. The CV of the annual maximum and minimum temperatures ranges from 1.35 to 5.92 and 1.58 to 14.79 respectively. During the autumn season, the maximum temperature CV ranges from 1.4 to 6.8 whereas the minimum temperature CV ranges from 1.61 to 17.15. The CV of the spring season maximum and minimum temperature ranges from 1.97 to 6.48 and 2.62 to 15.81 respectively. In the summer season, the maximum and minimum CV ranges from 2.20 to 7.15 and 1.83 to 17.57 respectively. The winter season maximum and minimum CV ranges from 1.71 to 7.97 and 1.34 to 15.39 respectively. Both maximum and minimum temperature CV are categorized under less variability (CV <20 %).

**Fig. 11:**
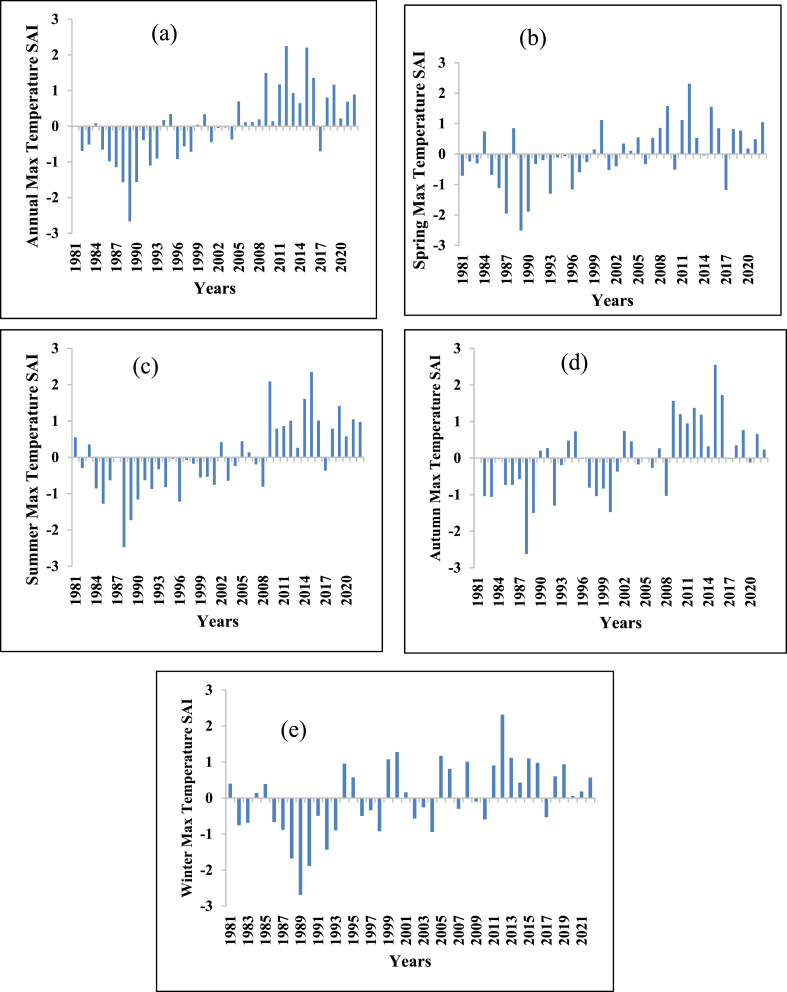
Standard anomaly index of annual and seasonal maximum temperature (a) Annual (b) Spring (c) Summer (d) Autumn (e) Winter.

**Fig. 12:**
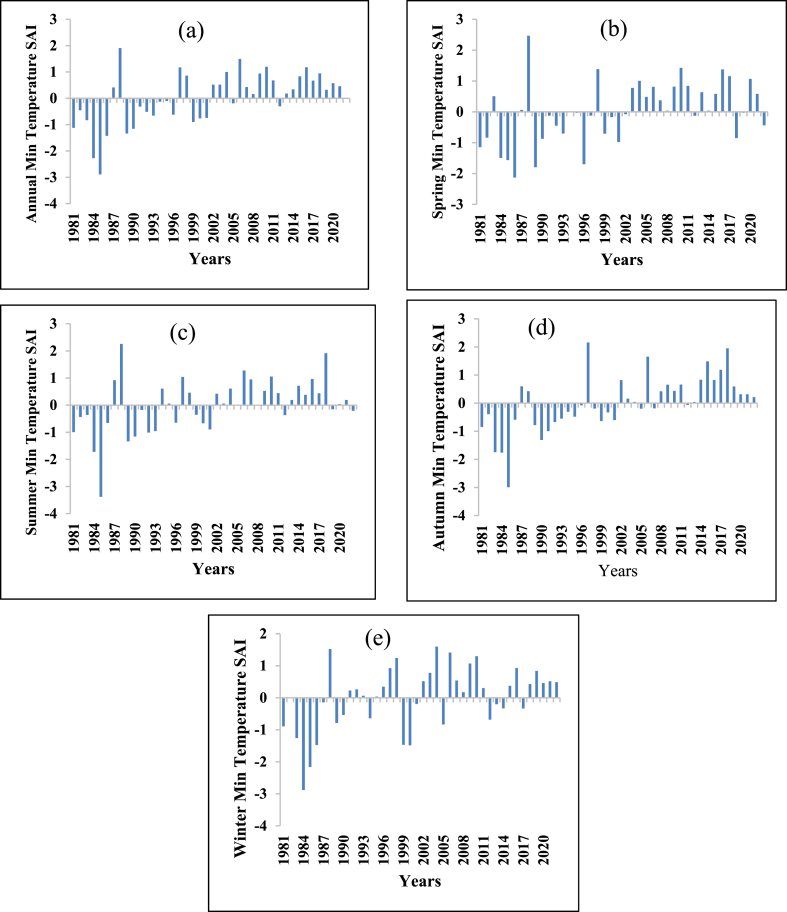
Standard anomaly index of annual and seasonal minimum temperature (a) Annual (b) Spring (c) Summer (d) Autumn (e) Winter.

The SAI of the maximum and minimum temperatures are shown in Fig. 11, Fig. 12 respectively. The annual maximum temperature standardized anomaly index (SAI) was 57.15 % positive (warming period) and 42.85 % negative (Cooling period). The maximum temperature of the winter season had 59.53 % positive SAI (warming period) and 40.47 % negative SAI (cooling period). The maximum temperatures of the spring season showed 54.76 % positive SAI (warming period) and 45.24 % negative SAI (cooling period). The maximum temperature of the summer season showed 45.24 % positive SAI (warming period) and 54.76 negative SAI (cooling period). The maximum temperature of the autumn season showed 52.38 % positive SAI (warming period) and 47.62 % negative SAI (cooling period). Except for the summer season, all seasons have higher positive SAI (warming period) than negative SAI (cooling period). Similar results were observed in the Zarima Sub-Basin north-western Ethiopia [16]; Addis Ababa city, Ethiopia [109]; and Southern Ethiopia [110].

#### 3.4.2 Trend analysis of the annual and seasonal temperature

##### 3.4.2.1 Modified Mann-Kendall method for maximum temperature and minimum temperature

**Fig. 13:**
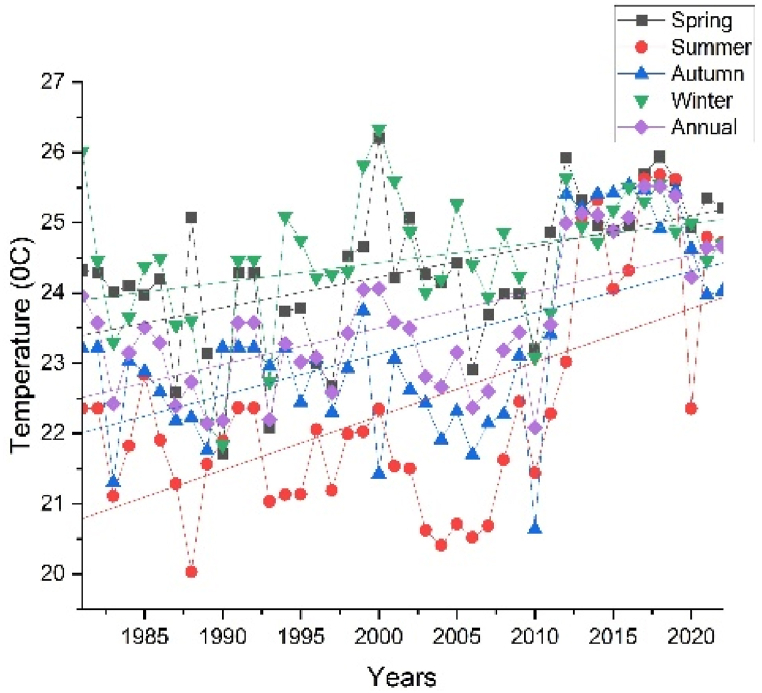
Annual and seasonal maximum temperature trend of Dedo station.

**Fig. 14:**
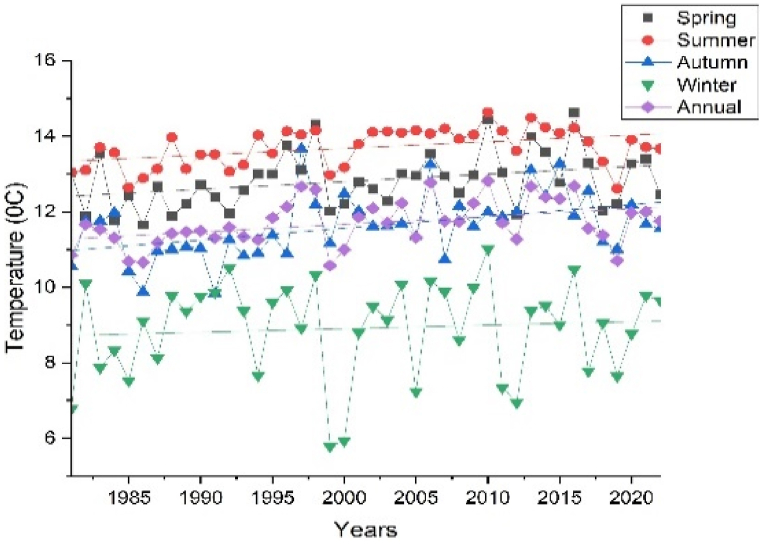
Annual and seasonal minimum temperature of Jimma station.

The annual and seasonal representations of the trend using MMK and Senʼs are given in Table 5, Table 6. In the spring, summer, autumn, and winter seasons the maximum significantly increasing trend (P < 0.05) of the maximum temperature was 0.25 °C/year, 0.15 °C/year, 0.043 °C/year, and 0.082 °C/year respectively, while the annual maximum significantly increasing trend was 0.083 °C/year. In the Annual 10 stations (Chida, Chira, Dedo, Deri Goma, Limugenet, Sekoru, Shebe, Wolayita, Woliso, and Wolkite) out of 16 showed a significantly increasing trend whereas the remaining stations (Assendabo, Bele, Bonga, Gedo, Hosaina and Jimma) showed an increasing trend. In the Spring season, only the Gedo station showed a decreasing trend, while other stations showed an increasing trend. From those stations, 11 stations (Chira, Dedo, Deri Goma, Hosaina, Jimma, Limugenet, Sekoru, Shebe, Wolayita, Woliso, and Wolkite) showed a significantly increasing trend and the remaining showed a decreasing trend. In the summer seasons, only Bonga station showed a decreasing trend. In this season maximum stations of the basin showed an increasing trend. In the Autumn season, 4 stations (Deri Goma, Limugenet, Shebe, and Woliso) showed a significantly increasing trend whereas others showed an increasing trend except Wolayita stations which showed a decreasing trend. Only Assendabo station shows a decreasing trend in the Winter season while the remaining 9 stations (Chira, Dedo, Deri Goma, Hosaina, Limugenet, Sekoru, Shebe, Woliso, and Wolkite) showed a significantly increasing trend and 6 stations (Bele, Bonga, Chida, Gedo, Jimma, and Wolayita) showed an increasing trend. The results of MK and Senʼs tests for temperature showed that significant increasing trends were observed in maximum temperature for the majority of the stations in the basin. In the Spring season, the minimum temperature significant increasing trend and significant decreasing trend showed values of 0.068 °C/year at Wolayita station and 0.047 °C/year at Chida station respectively. During the summer season, the minimum temperature significantly increasing and decreasing trend was 0.039 °C/year at Woliso station and 0.022 °C/year at Deri Goma station respectively. In the autumn season significantly increasing trend was 0.046 °C/year at Woliso station and no significantly decreasing trend in this season. During the winter season significantly increasing and significantly decreasing trends were 0.028 °C/year at Woliso station and 0.033 °C/year at Deri Goma station respectively. The annual minimum temperature significantly increasing trend was 0.036 °C/year at Wolayita station and no significantly decreasing trend. Similar observations of maximum and minimum temperature trends were observed in Southern Ethiopia by Ref. [110]; semi-arid Borana zone, Ethiopia by Ref. [111]; Jemma sub-basin of Upper Blue Nile, Ethiopia by Ref. [34]; Ajora-Woybo watershed, Omo-Gibe Basin, Ethiopia by Ref. [26]. The time series plot of annual and seasonal maximum and minimum temperature is presented in Fig. 13 (sample for Dedo station) and Fig. 14 (sample for Jimma station) respectively. All station's annual and seasonal maximum and minimum temperature trend graphs were included in the supplementary material. Generally, the maximum temperature increasing trend was greater than the minimum temperature trend in the Upper Omo Gibe Basin and both trend analyses showed that the Upper Omo Gibe Basin has been warming over the past 42 years. The rising temperature and decline in rainfall in Ethiopia are causing severe problems in the agricultural activity of the farmers, as 80 % of the population relies on rain-fed agriculture. Consequently, there is a pressing requirement to create strategies for adaptation and mitigation across different sectors of the economy, particularly in agriculture, water management, energy, and health. All station's annual and seasonal maximum and minimum temperature trend graphs were included in the supplementary material.

**Table 5:** Modified Mann-Kendall (Z) and Senʼs slope (S) values of annual and seasonal maximum temperature.

Stations	Spring		Summer		Autumn		Winter		Annual	
	Z(MMK)	Sen	Z(MMK)	Sen	Z(MMK)	Sen	Z(MMK)	Sen	Z(MMK)	Sen
Assendabo	0.271	0.004	1.544	0.032	0.796	0.006	−0.305	−0.045	1.210	0.015
Bele	0.000	0.000	0.836	0.002	0.888	0.001	1.263	0.015	0.795	0.005
Bonga	0.498	0.005	−0.145	−0.001	1.322	0.012	1.367	0.016	1.122	0.006
Chida	1.829	0.002	3.409	0.032	1.839	0.010	1.246	0.001	2.240	0.021
Chira	3.379	0.024	0.643	0.003	1.075	0.003	2.475	0.016	2.744	0.016
Dedo	3.306	0.041	1.423	0.063	1.496	0.048	2.650	0.027	2.586	0.054
Deri Goma	2.164	0.023	2.349	0.016	3.496	0.004	4.224	0.030	2.994	0.025
Gedo	−0.118	−0.003	3.695	0.064	0.802	0.012	0.015	0.001	1.586	0.0185
Hosaina	2.381	0.0186	0.421	0.006	1.110	0.003	2.612	0.012	1.093	0.006
Jimma	2.501	0.0345	1.745	0.014	1.662	0.015	1.600	0.024	1.609	0.023
Limugenet	3.117	0.013	2.845	0.008	2.517	0.005	3.128	0.015	3.468	0.009
Sekoru	3.133	0.024	3.133	0.024	1.144	0.010	2.023	0.023	1.969	0.018
Shebe	3.009	0.039	2.293	0.027	3.122	0.027	2.655	0.031	4.074	0.032
Wolayita	2.147	0.024	0.672	0.0054	−0.122	0.001	1.831	0.014	2.005	0.012
Woliso	2.558	0.022	3.046	0.019	2.047	0.017	3.990	0.014	4.348	0.018
Wolkite	3.925	0.094	0.821	0.021	1.841	0.038	3.755	0.074	2.629	0.061

**Table 6:** Modified Mann-Kendall (Z) and Senʼs slope (S) values of annual and seasonal minimum temperature.

Station	Spring		Summer		Autumn		Winter		Annual	
	Z(MMK)	Sen	Z(MMK)	Sen	Z(MMK)	Sen	Z(MMK)	Sen	Z(MMK)	Sen
Assendabo	−1.576	−0.061	−1.674	−0.089	−1.101	−0.045	−0.735	−0.027	−1.249	−0.059
Bele	−0.484	0.001	0.395	0.001	0.879	0.001	0.724	0.001	0.415	0.001
Bonga	1.260	0.015	−0.161	−0.003	0.932	0.009	1.741	0.021	2.282	0.020
Chida	−4.085	−0.005	−0.133	0.001	0.256	0.001	−0.123	−0.001	−0.745	−0.001
Chira	3.255	0.024	2.855	0.013	2.359	0.015	1.755	0.014	2.940	0.019
Dedo	2.305	0.015	2.682	0.033	1.878	0.028	1.637	0.028	2.357	0.030
Deri Goma	−0.741	−0.004	−2.158	−0.017	−1.412	−0.005	−2.083	−0.027	−1.549	−0.019
Gedo	2.296	0.057	3.399	0.076	2.992	0.071	2.573	0.069	2.637	0.069
Hosaina	1.619	0.019	2.818	0.023	1.702	0.014	−0.975	−0.012	1.325	0.014
Jimma	3.321	0.023	2.401	0.017	2.991	0.034	0.552	0.009	2.839	0.021
Limugenet	2.900	0.005	3.146	0.007	3.539	0.010	2.042	0.009	3.074	0.005
Sekoru	1.919	0.019	0.955	0.018	1.671	0.027	3.090	0.027	1.923	0.027
Shebe	−0.424	−0.001	0.330	0.001	−0.188	−0.001	1.104	0.001	1.626	0.001
Wolayita	4.121	0.029	3.333	0.025	3.180	0.026	3.068	0.019	3.422	0.028
Woliso	4.022	0.035	3.899	0.030	4.769	0.037	3.816	0.023	1.567	0.013
Wolkite	0.839	0.011	1.975	0.016	1.127	0.004	1.163	0.010	1.122	0.011

##### 3.4.2.2 Using ITA for maximum temperature

**Fig. 15:**
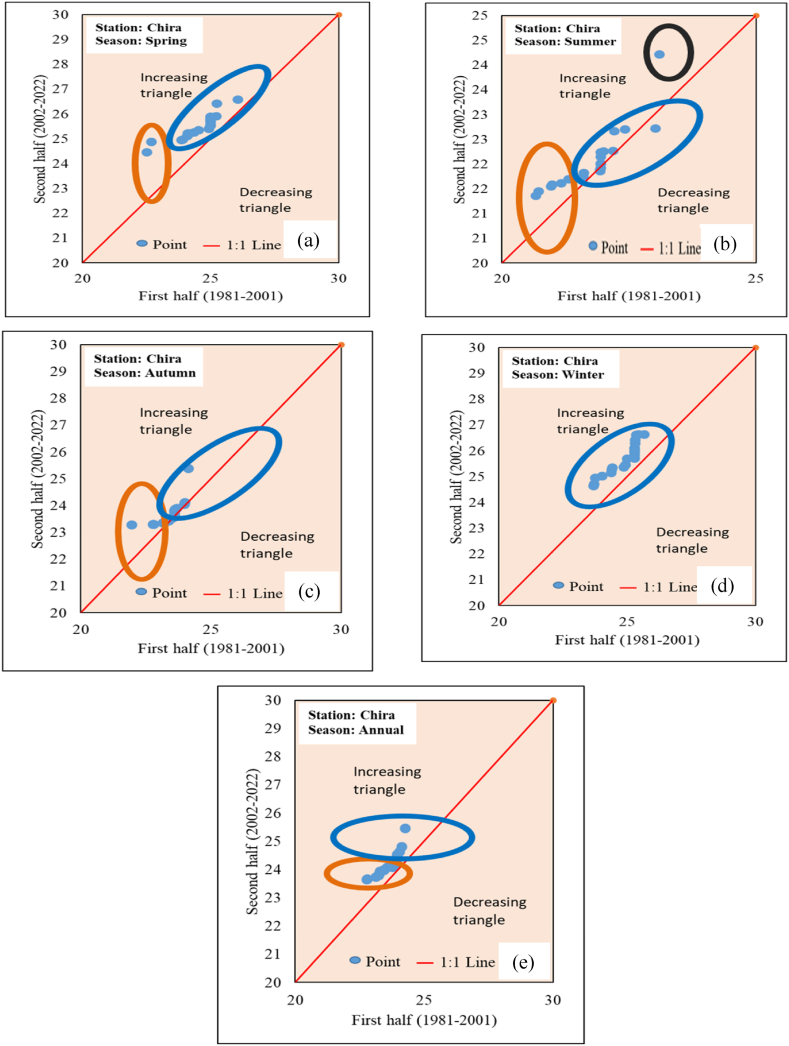
Annual and seasonal maximum temperature ITA graphical result of Chira station (a) Spring (b) Summer (c) Autumn (d) Winter (e) Annual.

The ITA method for trend analysis of maximum temperature was observed in 16 stations for annual and seasonal measurements. The trend indicator of seasonal and annual maximum temperature is presented in Table 7 and Fig. 15 (sample for Chira station). In the spring and summer seasons, all stations except Bonga station showed an increasing trend. In the Autumn season, all stations showed an increasing trend. In the winter season, only the Bonga and Gedo stations showed a decreasing trend, while the remaining 14 stations showed an increasing trend. In the annual season, only the Bonga station showed a decreasing trend, and the rest 15 stations showed an increasing trend. Generally increasing maximum temperatures were observed in the Upper Omo Gibe Basin using the ITA method. In addition to trend indicator the numerical observation, the “low”, “medium” and “high” categories trend was observed using a graphical method. In the Spring season, “low” categories of the dataset showed an increasing trend in all stations. “Medium” categories dataset except Bonga station showed an increasing trend. All “high” categories datasets showed an increasing trend. In the Summer season, only the Woliso and Shebe stations in the “low” categories showed a decreasing trend while the remaining stations showed an increasing trend. In “medium” categories except for Bonga stations all stations exhibited an increasing trend. In “higher” categories all stations showed an increasing trend. In the autumn season, all “low” categories showed an increasing trend. The “medium” categories all stations showed an increasing trend except Bonga and Hosaina stations. The higher categories of all stations exhibited an increasing trend. In the winter season, the record of “low” categories is very low. Only two stations were categorized as low. From those Dedo stations exhibited an increasing trend whereas Gedo stations exhibited a decreasing trend. In the “medium” categories except for Shebe and Bele stations, all stations exhibited an increasing trend. From “higher” categories all stations showed an increasing trend. In annual observation rainfall increasing trends were observed in all categories.

**Table 7:** Innovative trend analysis trend indicator of seasonal and annual maximum temperature.

Station	Spring	Summer	Autumn	Winter	Annual
Assendabo	0.134	0.341	0.174	0.055	0.170
Bele	0.074	0.190	0.099	0.037	0.097
Bonga	−0.233	−0.343	0.024	−0.163	−0.178
Chida	0.205	0.468	0.064	0.044	0.187
Chira	0.389	0.140	0.119	0.352	0.253
Dedo	0.369	0.580	0.434	0.177	0.385
Deri Goma	0.376	0.182	0.187	0.352	0.276
Gedo	0.078	0.918	0.318	−0.027	0.294
Hosaina	0.244	0.084	0.119	0.176	0.159
Jimma	0.270	0.172	0.120	0.185	0.187
Limu Genet	0.141	0.065	0.134	0.143	0.122
Sekoru	0.207	0.184	0.095	0.180	0.166
Shebe	0.124	0.190	0.149	0.051	0.126
Wolayita	0.194	0.159	0.096	0.123	0.142
Woliso	0.214	0.244	0.219	0.168	0.209
Wolkite	0.811	0.285	0.657	0.817	0.650

##### 3.4.2.3 ITA method for minimum temperature

**Fig. 16:**
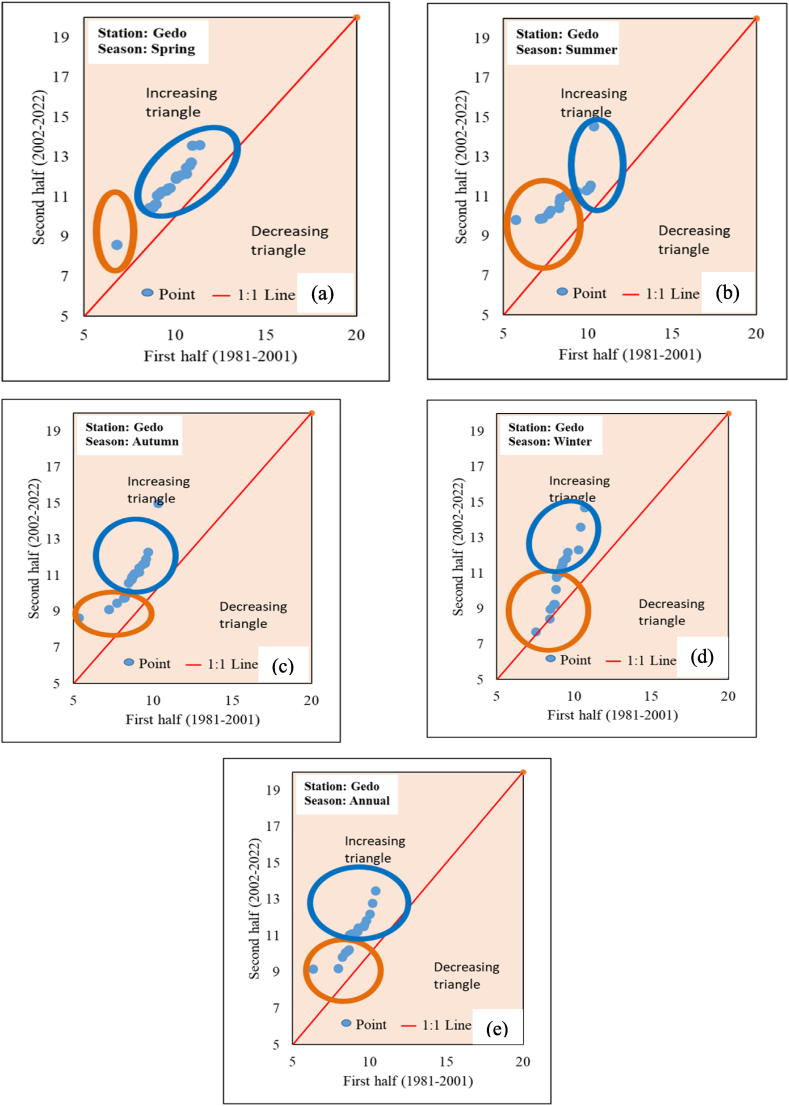
Annual and seasonal maximum temperature ITA graphical result of Gedo station (a) Spring (b) Summer (c) Autumn (d) Winter (e) Annual.

The minimum temperature of 16 stations in the Upper Omo Gibe Basin using the ITA method was computed. The trend indicator of seasonal and annual minimum temperature is shown in Table 8 and Fig. 16 (sample for Gedo station). In the spring season Assendabo, Bele, Chida and Deri Goma stations showed a decreasing trend, while the remaining 14 stations showed an increasing trend. In the summer and autumn seasons, Assendabo, Chida, and Deri Goma stations showed a decreasing trend, while the remaining 13 stations showed an increasing trend. In the winter season Assendabo, Bele, Chida, Deri Goma, and Hosaina stations showed a decreasing trend, while the remaining 11 stations showed an increasing trend. In the annual observation of the minimum temperature Assendabo, Chida, and Deri Goma stations showed a decreasing trend and the remaining 13 stations showed an increasing trend. Generally, increasing minimum temperatures were observed in the Upper Omo Gibe Basin using the ITA method. In addition to trend indicator numerical observation, the “low”, “medium” and “high” categories trend was observed using a graphical method. In the spring season, the “low” categories increasing trend was observed in Chira, Dedo, and Gedo stations. In the “medium” categories all stations showed increasing trends except Bonga and Chida stations. In “high” categories all stations showed an increasing trend. In the summer season under “lower” categories except for Bonga, Limugenet, and Shebe all other stations showed an increasing trend. In the “medium” categories except for Bonga station, all stations showed an increasing trend. In “high” categories all stations showed increasing trends. In the Autumn season, all “low” categories showed an increasing trend. In the “medium” categories all stations except Bonga showed an increasing trend. In “high” categories all increasing trends were observed. In the winter season “low” categories were observed only in Dedo and Gedo stations. Dedo stations showed an increasing trend while Gedo stations showed a decreasing trend. In the “medium” categories all stations except Bele showed an increasing trend. In the “high” categories all stations exhibited an increasing trend. In the Annual season under “low” categories, all stations showed an increasing trend. In the “medium” categories all stations except Shebe showed an increasing trend. “High” categories observation in all stations showed an increasing trend. All stations annual and seasonal maximum and minimum temperature ITA trend graphs were included in the supplementary material.

**Table 8:** Innovative trend analysis trend indicator of seasonal and annual minimum temperature.

Station	Spring	Summer	Autumn	Winter	Annual
Assendabo	−1.195	−1.519	−1.159	−0.667	−1.167
Bele	−0.010	0.124	0.055	−0.008	0.040
Bonga	0.628	0.483	0.292	0.931	0.542
Chida	−0.249	−0.113	−0.061	−0.011	−0.110
Chira	0.702	0.344	0.314	0.468	0.459
Dedo	0.728	0.879	1.104	0.728	0.855
Deri Goma	−0.111	−0.775	−0.623	−0.736	−0.563
Gedo	1.959	2.712	2.630	1.997	2.312
Hosaina	0.305	0.314	0.251	−0.225	0.175
Jimma	0.337	0.383	0.580	0.408	0.443
Limugenet	0.254	0.211	0.319	0.348	0.278
Sekoru	0.368	0.219	0.417	0.511	0.376
Shebe	0.001	0.000	0.000	0.000	0.001
Wolayita	0.493	0.501	0.461	0.250	0.426
Woliso	0.564	0.772	0.965	0.639	0.728
Wolkite	0.553	0.518	0.467	0.476	0.504

### 3.5 Annual and seasonal stream flow variability and trend

#### 3.5.1 Annual and seasonal stream flow variability

The mean annual streamflow computed in the period 1985 to 2018 was 149.48 m^3^/s (Gilgel Gibe at Abelti), 61.72 m^3^/s (Gojeb), 41.95 m^3^/s (Gilgel Gibe at Assendabo), 33.55 m^3^/s (Wabi), 9.85 m^3^/s (Guma), 8.37 m^3^/s (Bulbul), 4.97 m^3^/s (Gibe), 3.88 m^3^/s (Gecha), 2.91 m^3^/s (Megecha) and 0.39 m^3^/s (Bidru Awana). The CV was used to examine the variability of different streamflow in the Upper Omo Gibe Basin. The CV of the streamflow of different gauging stations is shown in Table 9. Based on the CV analysis, all gauging stations showed extremely high variability (CV > 70) during the summer season. Similar results were obtained in the Ajora Woybo watershed in the Omo Gibe Basin in Ethiopia [26]. During the annual observation Bulbul, Gibe, Gilgel Gibe at Abelti, Gilgel Gibe at Assendabo, Gojeb, and Megecha gauging stations showed moderate variability (20 < CV < 30). Bidru Awana and Guma gauging stations showed very high variability (CV > 40) whereas Gecha and Wabi gauging stations showed high variability (CV > 30). In the spring season, Gecha, Gibe Gilgel Gibe at Abelti, Gilgel Gibe at Assendabo, Gojeb, and Guma gauging stations showed very high variability (CV > 40) whereas Bidru Awana, Bulbul, Megecha and Wabi gauging stations showed extremely high variability (CV > 70). During the autumn season, Gibe and Megech gauging stations showed moderate variability (20 <CV < 30) whereas Gecha, Gilgel Gibe at Abelti, Gilgel Gibe at Assendabo, Gojeb, and Wabi gauging stations showed high variability (CV > 30). In this season Bidru Awana and Bulbul gauging stations showed very high variability (CV > 40) whereas the Guma station showed extremely high variability (CV > 70). In the winter season, the Megecha gauging station showed high variability (CV > 30) whereas the Gecha gauging station showed extremely high variability (CV > 70). The rest stations Bidru Awana, Bulbul, Gibe, Gilgel Gibe at Abelti, Gilgel Gibe at Assendabo, Gojeb, Guma, and Wabi gauging stations showed very high variability (CV > 40). Similar observations were obtained in the Awash River Basin [18].

**Table 9:** Coefficient of variation of streamflow.

Gauging Stations	Annual	Spring	Summer	Autumn	Winter
Bidru Awana	45.30	76.09	114.39	49.69	60.03
Bulbul	27.46	82.51	104.33	47.07	45.43
Gecha	32.91	53.76	106.10	32.09	106.61
Gibe	22.97	48.05	103.64	25.02	42.06
Gilgel Gibe at Abelti	28.17	51.32	106.79	32.45	50.23
Gilgel Gibe at Assendabo	24.28	58.46	105.60	34.32	60.54
Gojeb	23.30	54.49	105.46	35.68	53.05
Guma	48.56	59.40	112.07	72.80	49.66
Megecha	29.39	71.93	108.19	25.38	34.31
Wabi	34.07	85.51	107.86	37.35	65.61

**Fig. 17:**
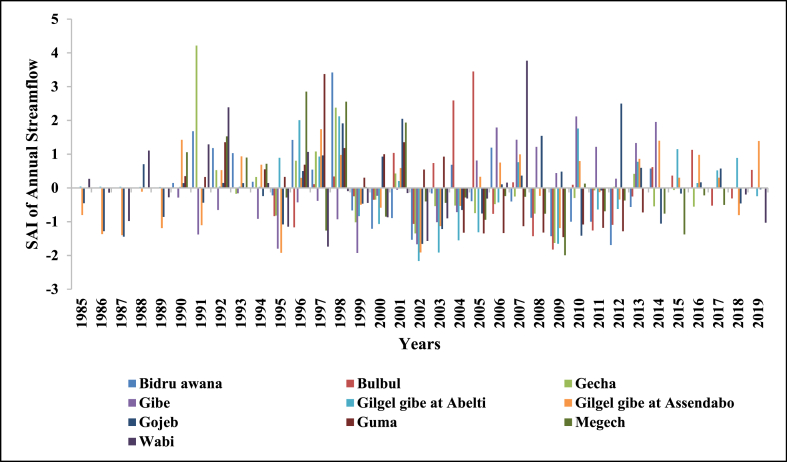
Standard anomaly index of annual streams of Upper Omo Gibe Basin.

**Fig. 18:**
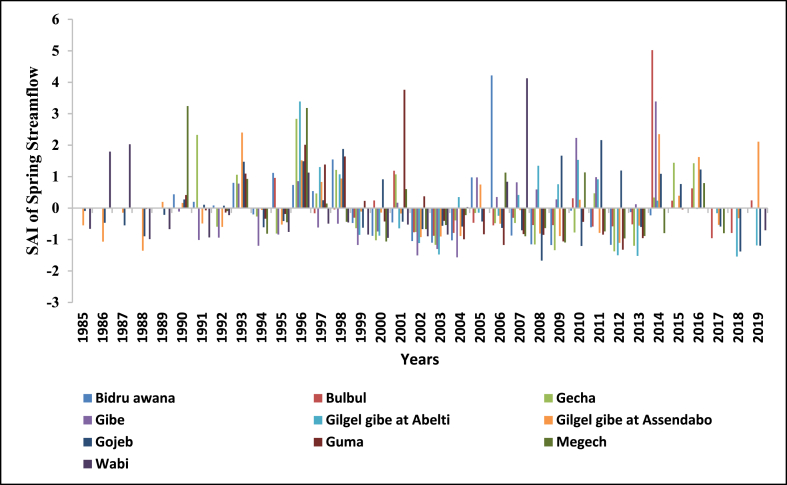
Standard anomaly index of spring season streams of Upper Omo Gibe Basin.

**Fig. 19:**
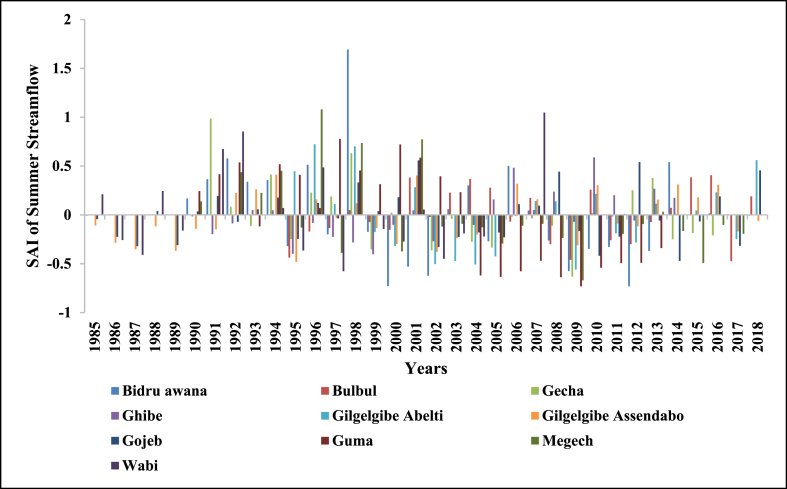
Standard anomaly index of summer season streams of Upper Omo Gibe Basin.

**Fig. 20:**
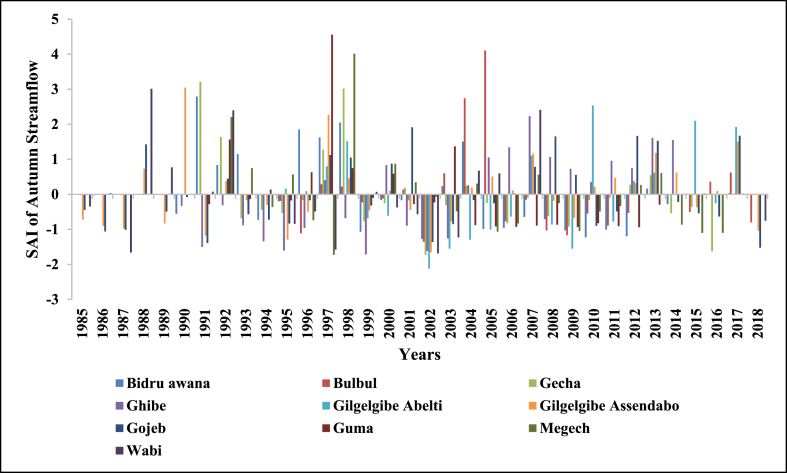
Standard anomaly index of autumn season streams of Upper Omo Gibe basin.

**Fig. 21:**
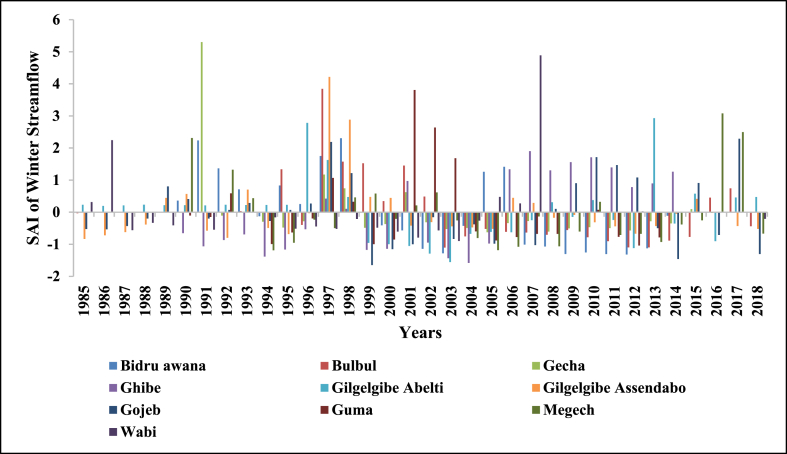
Standard anomaly index of winter season streams of Upper Omo Gibe basin.

SAI was used to determine the driest and wettest years in the observed years (1985–2018) of streamflow. The number of years having negative SAI was more than years with positive SAI in annual and all seasons which indicates Upper Omo Gibe Basin experiences more dry years than wet years. Similar observations were observed in the Bilate watershed, Ethiopia [1]; in the Geba River Basin, sub-basin, Ethiopia [6]; Zarima sub-basin, north-western Ethiopia [16]. The SAI values of streamflow of the Upper Omo Gibe Basin different rivers are given below in Fig. 17, Fig. 18, Fig. 19, Fig. 20, Fig. 21.

#### 3.5.2 Trend analysis of the annual and seasonal stream flow

##### 3.5.2.1 Modified Mann Kendall method for streamflow

**Fig. 22:**
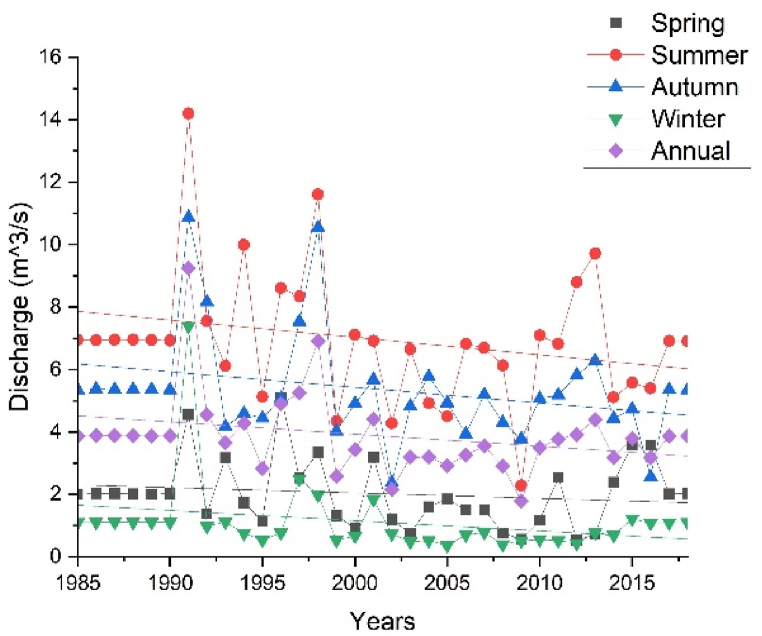
Annual and seasonal trend of streamflow of Gecha gauging station.

According to the result of MMK significant increase in streamflow (P < 0.05) was observed at the Gibe gauging station (Z = 2.02) and Gilgel Gibe at Assendabo station (Z = 2.02) in the summer season. A significant decrease trend of streamflow was observed in the Bulbul gauging station in the spring season (Z = −2.20), Megech gauging station in the summer season (Z = −2.19), and the annual (Z = −2.34). In the summer season, the Gibe gauging station significantly increasing trend at the magnitude of 0.2957m^3^/s whereas the Gilgel Gibe at Assendabo station magnitude of 7.6566m^3^/s and the Megech gauging station showed a significantly decreasing trend at the magnitude of 0.3226m^3^/s. A time series plot of annual and seasonal streamflow is presented in Fig. 22 (sample for Gecha gauging station). The Bulbul gauging station showed a significantly decreasing trend of magnitude of 0.0148 m^3^/s and the Megech gauging station showed a significantly decreasing trend magnitude of 0.0595 m^3^/s. In all seasons and annual streamflow, the decreasing trend of the streamflow was greater than an increasing trend in the Upper Omo Gibe Basin. Similar decreasing trends of streamflow were observed in the Birr watershed, Abay Basin, Ethiopia [112]; Bilate watershed, Ethiopia [1]; Geba River Basin, Ethiopia [6]; Awash River Basin, Ethiopia [41]. MMK and Sens results of the Upper Omo Gibe Basin are illustrated in Table 10. All gauging station streamflow trend graphs were included in the supplementary material.

**Table 10:** Modified Mann-Kendall (Z) and Senʼs slope (S) values of annual and seasonal streamflow.

Station	Spring		Summer		Autumn		Winter		Annual	
	Z(MMK)	Sen	Z(MMK)	Sen	Z(MMK)	Sen	Z(MMK)	Sen	Z(MMK)	Sen
Bidru awana	−0.947	−0.001	−1.079	−0.005	−1.093	−0.003	−1.462	−0.002	−0.906	−0.003
Bulbul	−2.198	−0.013	0.451	0.008	−0.993	−0.011	−1.612	−0.013	0.068	0.001
Gecha	−0.866	−0.018	−1.391	−0.014	−1.117	−0.013	−1.389	−0.012	−1.377	−0.010
Gibe	1.306	0.012	2.017	0.033	1.533	0.061	1.100	0.021	1.807	0.028
Gilgel gibe at Abelti	−1.309	−0.089	−0.127	−0.092	0.198	0.041	−0.445	−0.155	−0.057	−0.014
Gilgel gibe at Assendabo	0.596	0.047	2.016	0.903	1.278	0.476	0.260	0.008	1.817	0.351
Gojeb	−0.947	−0.124	0.710	0.466	0.795	0.376	0.271	0.025	1.065	0.260
Guma	−1.548	−0.071	−1.134	−0.309	−1.367	−0.096	−0.423	−0.001	−1.028	−0.068
Megech	−1.519	−0.013	−2.187	−0.046	−1.349	−0.011	−1.065	−0.001	−2.343	−0.021
Wabi	0.550	0.008	0.0316	0.005	−0.639	−0.009	0.432	0.003	−0.415	−0.009

##### 3.5.2.2 ITA method for streamflow

**Fig. 23:**
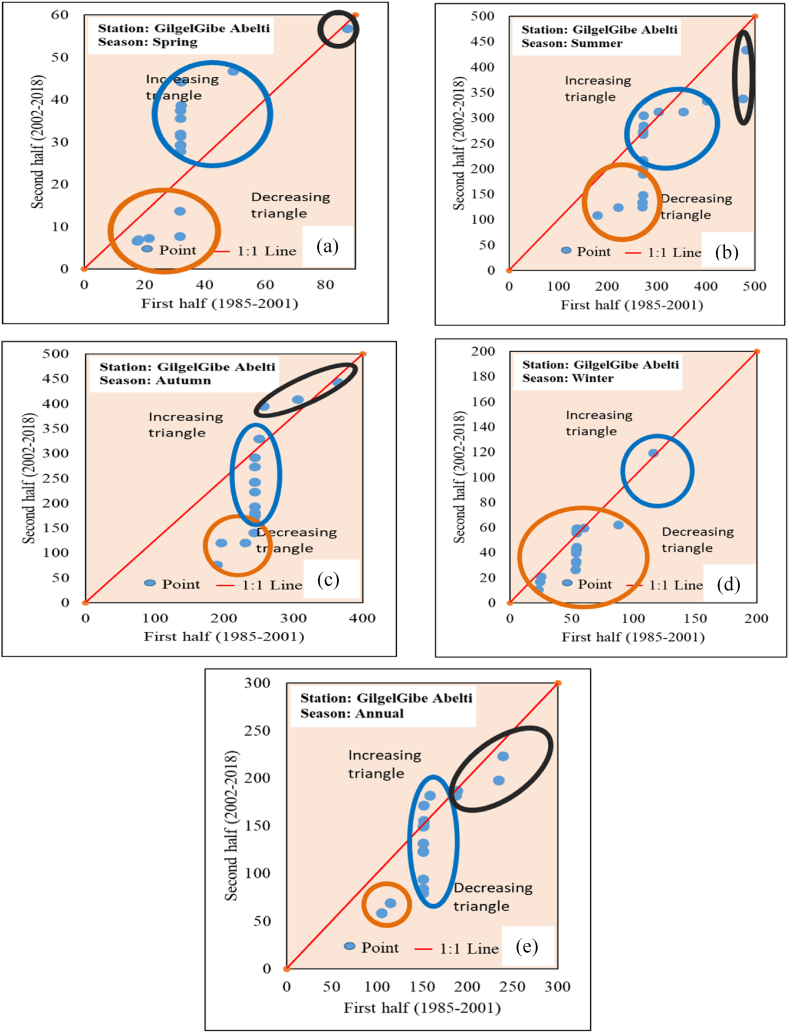
Annual and seasonal ITA graphical result of streamflow of Gilgel gibe at Abelti station (a) Spring (b) Summer (c) Autumn (d) Winter (e) Annual.

The ITA method was used for trend analysis of 10 streamflow gauging stations in the Upper Omo Gibe Basin as shown in Fig. 23 (sample for Gilgel Gibe at Abelti station) and Table 11. In the spring season, only Gibe and Wabi streamflow gauging stations showed an increasing trend while the remaining 8 streamflow gauging stations showed a decreasing trend. In the summer season, 7 streamflow gauging stations showed a decreasing trend whereas Bulbul, Gibe, and Gilgel Gibe at Assendabo streamflow gauging stations showed an increasing trend. During the Autumn season Bidru Awana, Gecha, Gilgel Gibe at Abelti, Guma, Megech, and Wabi streamflow gauging stations showed a decreasing trend whereas Bulbul, Gibe, Gilgel Gibe at Assendabo, and Gojeb gauging stations showed increasing trend. In the winter season, Gibe and Gojeb streamflow gauging stations showed a decreasing trend whereas the rest 8 streamflow gauging stations showed an increasing trend. In the Annual observation of streamflow Bidru Awana, Gecha, Gilgel Gibe at Abelti, Gojeb, Guma, and Megech streamflow gauging stations showed a decreasing trend whereas Bulbul, Gibe, Gilgel Gibe at Assendabo, and Wabi streamflow gauging stations were showed increasing trend as shown in Table 11. In addition to trend indicator numerical observation “low”, “medium” and “high” categories trend was observed using a graphical method. In the spring season under “low” categories decreasing trends were observed in the Guma, Megecha, and Wabi gauging stations whereas the other gauging stations exhibited increasing trends. In “medium” categories increasing trends were observed in Gilgel Gibe at Abelti, Megecha, Guma, and Gecha gauging stations, and other stations exhibited decreasing trends. Under “high” categories decreasing trend was observed in Gojeb, Guma, and Gilgel Gibe at Abelti stations, and other stations exhibited an increasing trend. In the summer season under “low” categories Gilgel gibe at Abelti and Guma gauging stations exhibited a decreasing trend and other stations exhibited an increasing trend. In the “medium” categories Bulbul, Gibe, Gojeb, Guma, and Wabi gauging stations observed a decreasing trend whereas the other gauging stations exhibited an increasing trend. In “high” categories Bidru awana, Gibe, Gilgel Gibe at Abelti, and Megecha gauging stations observed a decreasing trend whereas the other gauging stations exhibited a decreasing trend. In the autumn season under “low” categories Bulbul, Gilgel Gibe at Abelti, and Guma gauging stations observed a decreasing trend the other gauging stations exhibited an increasing trend. In the “medium” categories Bulbul, Gibe and Gilgel Gibe at Abelti gauging stations showed a decreasing trend and the other stations showed an increasing trend. In “high” categories Guma and Wabi gauging stations showed a decreasing trend and the other stations showed an increasing trend. In the winter season in “low” categories Bidru awana, Gilgel Gibe at Abelti, Gojeb, Guma, Megecha and Wabi gauging stations showed a decreasing trend and the other stations showed an increasing trend. In the “medium” categories Bidru awana, Bulbul, and Wabi gauging stations showed a decreasing trend and the other stations showed an increasing trend. In the “high” categories Bulbul, Gecha, and Wabe gauging stations showed a decreasing trend, and other stations showed an increasing trend. In the Annual streamflow observation under “low” categories, all stations exhibited a decreasing trend. In the “medium” categories Bidru Awana, Bulbul, Gibe, Gilgel Gibe at Abelti, Gojeb, and Wabi stations showed a decreasing trend whereas the other stations exhibited an increasing trend. In “high” categories Bidru Awana, Gibe, Gilgel Gibe at Abelti, Gojeb, Guma and Megecha gauging stations showed decreasing trend and other stations showed an increasing trend. All gauging station's annual and seasonal streamflow ITA trend graphs were included in the supplementary material.

**Table 11:** Innovative trend analysis trend indicator of seasonal and annual streamflow.

Station	Spring	Summer	Autumn	Winter	Annual
Bidru Awana	−2.676	−2.431	−3.544	−4.693	−2.992
Bulbul	−1.767	0.592	0.479	−4.002	0.283
Gecha	−3.017	−2.051	−2.179	−5.342	−2.505
Gibe	3.553	2.246	3.051	4.323	2.824
Gilgel Gibe at Abelti	−1.531	−1.988	−0.475	−1.825	−1.370
Gilgel Gibe at Assendabo	−1.252	0.703	0.507	−2.444	0.329
Gojeb	−0.926	−0.773	0.254	0.564	−0.316
Guma	−4.927	−4.899	−4.743	−1.003	−4.701
Megech	−3.599	−3.165	−1.612	−0.870	−2.734
Wabi	3.333	−0.273	−0.103	2.948	0.042

### 3.6 Comparison of trend analysis method

The comparison between the trend detection results of the MMK and ITA methods for rainfall, maximum, minimum temperature, and streamflow were presented in Table 12, Table 13, Table 14, Table 15 respectively. Statistically significant increasing or decreasing trends observed were 22 (27.5 %) for rainfall, 43 (53.75 %) for maximum temperature, 39 (48.75 %) for minimum temperature, and 5 (10 %) for streamflow with the MMK test. In the ITA method 30 (37.5 %) for rainfall, 47 (58.75 %) for maximum temperature, 44 (55 %) for minimum temperature, and 34 (68 %) for streamflow were obtained. The ITA method showed a more significant increase or decrease than the MMK method, due to its sensitivity data to data distribution and localized variations. Unlike MMK, which relies on rank-based statistics and adjustments for autocorrelations, ITA directly evaluates data values, effectively detecting minor trends and outliers that MMK might miss [33,113]. This indicated that the ITA method displays stronger significant trends than the MMK method [100,114,115]. ITA is considered better than the MMK test at detecting trends because it can detect non-monotonic trends, which the MMK test cannot. Unlike the MMK test, which only identifies monotonic trends, ITA can identify trends at different levels of a time series including low and high. ITA has been effectively used to analyze various climatic parameters, demonstrating its capability to identify trends in more parameters than the MMK test method. Moreover, ITA is not constrained by factors such as data length, distribution type, or serial correlation, this makes more adaptable and reliable method. In conclusion, the ITA method provides a more innovative and comprehensive approach to trend analysis than the traditional MMK method.

**Table 12:** Comparison of MMK and ITA of rainfall.

Station	Spring		Summer		Autumn		Winter		Annual	
	MMK	ITA	MMK	ITA	MMK	ITA	MMK	ITA	MMK	ITA
Assendabo	↑∗	↑∗	↑	↑	↑∗	↑	↑	↑∗	↑∗	↑∗
Bele	↓	↓	↓	↑	↑	↑	↓	↓	↑	↑
Bonga	↑	↑	↑	↑	↑∗	↑∗	↓	↓	↑	↑
Chida	↑	↑	↑	↑	↑	↑∗	↓	↓∗	↑	↑
Chira	↑	↑	↓	↓	↑	↑	↓	↓∗	↓	↓
Dedo	↓∗	↓∗	↓	↓∗	↓∗	↓∗	↓	↓∗	↓∗	↓∗
Deri Goma	↓	↓∗	↓∗	↓∗	↓	↑	↓∗	↓∗	↓∗	↓∗
Gedo	↑	↓	↑	↑∗	↑∗	↑∗	↓	↓	↑	↑∗
Hosaina	↓	↓	↑	↑	↑	↓	↓	↓∗	↓	↓
Jimma	↑	↑	↑∗	↑	↑	↑	↑	↑	↑∗	↑
Limugenet	↑∗	↑∗	↑∗	↑∗	↑∗	↑∗	↓	↓	↑∗	↑∗
Sekoru	↑	↓	↓	↓	↑	↓	↓	↓∗	↓	↓
Shebe	↑	↓	↑	↑	↑	↓	↓∗	↓∗	↓	↓
Wolayita	↑	↑	↓	↑	↑	↑	↓	↓∗	↑	↑
Woliso	↓	↓	↓	↓	↑	↑	↓∗	↓	↓	↓
Wolkite	↓	↓∗	↓∗	↓	↓∗	↓	↓	↓∗	↓∗	↓∗

↑∗ and ↓∗ indicates statistically significant increasing and decreasing trends respectively at 5 % level, ↑ and ↓ indicates insignificant increasing and decreasing trends respectively.

**Table 13:** Comparison of MMK and ITA of maximum temperature.

Station	Spring		Summer		Autumn		Winter		Annual	
	MMK	ITA	MMK	ITA	MMK	ITA	MMK	ITA	MMK	ITA
Assendabo	↑	↑∗	↑	↑	↑	↑	↓	↑	↑	↑∗
Bele	↑	↑	↑	↑∗	↑	↑∗	↑	↑	↑	↑
Bonga	↑	↓	↓	↓	↑	↑	↑	↓	↑	↓
Chida	↑	↑	↑∗	↑∗	↑	↑	↑	↑	↑∗	↑∗
Chira	↑∗	↑∗	↑	↑	↑	↑	↑∗	↑∗	↑∗	↑∗
Dedo	↑∗	↑∗	↑	↑	↑	↑	↑∗	↑∗	↑∗	↑∗
Deri Goma	↑∗	↑∗	↑∗	↑∗	↑∗	↑∗	↑∗	↑∗	↑∗	↑∗
Gedo	↓	↑	↑∗	↑∗	↑	↑	↑	↓	↑	↑
Hosaina	↑∗	↑∗	↑	↑	↑	↑	↑∗	↑∗	↑	↑
Jimma	↑∗	↑∗	↑	↑	↑	↑	↑	↑	↑	↑
Limugenet	↑∗	↑∗	↑∗	↑∗	↑∗	↑∗	↑∗	↑∗	↑∗	↑∗
Sekoru	↑∗	↑∗	↑∗	↑∗	↑	↑	↑∗	↑∗	↑∗	↑∗
Shebe	↑∗	↑∗	↑∗	↑∗	↑∗	↑∗	↑∗	↑∗	↑∗	↑∗
Wolayita	↑∗	↑∗	↑∗	↑∗	↓	↑	↑	↑	↑∗	↑∗
Woliso	↑∗	↑∗	↑∗	↑∗	↑∗	↑∗	↑∗	↑∗	↑∗	↑∗
Wolkite	↑∗	↑∗	↑∗	↑∗	↑	↑	↑∗	↑∗	↑∗	↑∗

↑∗ and ↓∗ indicates statistically significant increasing and decreasing trends respectively at 5 % level, ↑ and ↓ indicates insignificant increasing and decreasing trends respectively.

**Table 14:** Comparison of MMK and ITA of minimum temperature.

Station	Spring		Summer		Autumn		Winter		Annual	
	MMK	ITA	MMK	ITA	MMK	ITA	MMK	ITA	MMK	ITA
Assendabo	↓	↓	↓	↓	↓	↓	↓	↓	↓	↓
Bele	↓	↓	↑	↑	↑	↑	↑	↓	↑	↑
Bonga	↑	↑∗	↓	↑∗	↑	↑∗	↑	↑	↑∗	↑∗
Chida	↓∗	↓∗	↓	↓	↑	↓	↓	↓	↓	↓
Chira	↑∗	↑∗	↑∗	↑∗	↑∗	↑∗	↑	↑∗	↑∗	↑∗
Dedo	↑∗	↑∗	↑∗	↑∗	↑	↑	↑	↑	↑∗	↑∗
Deri Goma	↓	↓	↓∗	↓∗	↓	↓	↓∗	↓∗	↓	↓
Gedo	↑∗	↑∗	↑∗	↑∗	↑∗	↑∗	↑∗	↑∗	↑∗	↑∗
Hosaina	↑	↑	↑∗	↑∗	↑	↑	↓	↓	↑	↑
Jimma	↑∗	↑∗	↑∗	↑∗	↑∗	↑∗	↑	↑	↑∗	↑∗
Limugenet	↑∗	↑∗	↑∗	↑∗	↑∗	↑∗	↑∗	↑∗	↑∗	↑∗
Sekoru	↑	↑	↑	↑	↑	↑	↑∗	↑∗	↑	↑
Shebe	↓	↑	↑	↑	↑	↑	↑	↑	↑	↑∗
Wolayita	↑∗	↑∗	↑∗	↑∗	↑∗	↑∗	↑∗	↑∗	↑∗	↑∗
Woliso	↑∗	↑∗	↑∗	↑∗	↑∗	↑∗	↑∗	↑∗	↑∗	↑∗
Wolkite	↑∗	↑∗	↑∗	↑∗	↑	↑	↑	↑	↑	↑

↑∗ and ↓∗ indicates statistically significant increasing and decreasing trends respectively at 5 % level, ↑ and ↓ indicates insignificant increasing and decreasing trends respectively.

**Table 15:** Comparison of MMK and ITA of streamflow.

Station	Spring		Summer		Autumn		Winter		Annual	
	MMK	ITA	MMK	ITA	MMK	ITA	MMK	ITA	MMK	ITA
Bidru Awana	↓	↓∗	↓	↓∗	↓	↓∗	↓	↓∗	↓	↓∗
Bulbul	↓∗	↓∗	↑	↑	↓	↑	↓	↓∗	↑	↑
Gecha	↓	↓∗	↓	↓∗	↓	↓∗	↓	↓∗	↓	↓∗
Gibe	↑	↑∗	↑∗	↓∗	↑	↑∗	↑	↑∗	↑	↑∗
Gilgel Gibe at Abelti	↓	↓∗	↓	↓∗	↑	↓	↓	↓∗	↓	↓∗
Gilgel Gibe at Assendabo	↑	↓∗	↑∗	↑	↑	↑	↑	↓∗	↑	↑
Gojeb	↓	↓	↑	↓	↑	↑	↑	↑	↑	↓
Guma	↓	↓∗	↓	↓∗	↓	↓∗	↓	↓∗	↓	↓∗
Megecha	↓	↓∗	↓∗	↓∗	↓	↓∗	↓	↓	↓∗	↓∗
Wabi	↑	↑∗	↑	↓	↓	↓	↑	↑∗	↓	↑

↑∗ and ↓∗ indicates statistically significant increasing and decreasing trends respectively at 5 % level, ↑ and ↓ indicates insignificant increasing and decreasing trends respectively.

## 4 Conclusions

This study investigated the long-term spatiotemporal variability of rainfall, temperature and stream flow in the Upper Omo Gibe Basin. The data were analyzed using the CV, SAI, MMK, ITA, and the Senʼs slope estimator methods. The annual rainfall of the Upper Omo Gibe Basin from 1981 to 2022 was spatially distributed ranging between 1037.04 mm and 2056.23 mm. The western, central, and southern parts of the basin recorded the highest amount of rainfall, while the northern and eastern parts of the basin recorded the lowest amount of rainfall. The coefficient of variation of mean annual rainfall was 10.66–38.92 % ranging from less to moderate. The CV of the Autumn season was 19.15–52.72 % ranging from low to very high. The CV of the spring season was 17.49–49.49 % ranging from low to very high. The CV of the summer season was 12.92–41.12 % ranging from low to very high. The CV of the winter season was 38.89–71.83 % ranging from high to extremely high variability. Less spatial and temporal rainfall variability was observed in the summer season and the annual time scale. Conversely, significant variability in rainfall was observed during the winter and autumn seasons. Considering the annual rainfall pattern during the study period, the percentages of negative and positive anomalies were 47.62 % and 52.38 % respectively. The direction and magnitude of the trend in both annual and seasonal varied across the Upper Omo Gibe basin. The annual and seasonal rainfall of the basin showed both statistically significant and statistically insignificant trends. The annual rainfall showed a statistically significant increasing trend at Assendabo (Z = 3.641, S = 11.046), Limugenet (Z = 3.641, S = 16.484) and Jimma (Z = 2.428, S = 7.539) stations whereas statistically significant decreasing trend was observed in Dedo (Z = −2.732, S = −31.099) and Deri Goma (Z = −3.121, S = −6.040) stations. In the spring season, a statistically significant increasing trend was observed in Assendabo (Z = 2.492, S = 3.471) and Limugenet (Z = 2.062, S = 4.358) stations whereas a statistically significant decreasing trend was observed in Dedo (Z = −5.464, S = −11.033) station. In the summer season statistically significant increasing trend was observed in Jimma (Z = 2.298, S = 3.412) and Limugenet (Z = 2.260, S = 6.171) stations whereas a statistically significant decreasing trend was observed in Deri Goma (Z = −2.924, S = −4.168) and Wolkite (Z = −2.189, S = −2.867) stations. In the Autumn season, Bonga (Z = 2.146, S = 2.859), Gedo (Z = 2.016, S = 2.070), and Limugenet (Z = 3.316, S = 6.421) stations showed a statistically significant increasing trend, and Dedo (Z = −3.099, S = −7.500) station were show statistically significant decreasing trend. In the winter season statistically significant decreasing trend was observed in Deri Goma (Z = −2.774, S = −1.347) and Shebe (Z = −4.492, S = −1.676) stations and there are no observations of a statistically significant increasing trend in this season. Based on the Senʼs slope estimator the annual rainfall significantly increasing trend magnitudes up to 338.94mm/decade and decreasing trend ranges up to 525.54mm/decade. A maximum decreasing trend was observed in the winter season and a maximum increasing trend was observed in the autumn season. Generally annual (S = −0.21031), winter (S = −0.80569), and summer (S = −0.113) showed decreasing trend whereas spring (S = 0.08825) and autumn (S = 1.02075) showed an increasing trend. Insignificant trends were dominant both in annual and seasonal rainfall data.

The average maximum temperature of the Upper Omo Gibe Basin ranges from 20.1 °C to 31.83 °C whereas the average minimum temperature ranges from 8.92 °C to 18.19 °C. Annual maximum temperature standardized anomaly index (SAI) showed 57.15 % positive (warming period) and 42.85 % negative (Cooling period). Both maximum and minimum temperature CV are categorized under less variability (CV < 20 %). In spring, summer, autumn, and winter seasons the maximum significantly increasing trend (P < 0.05) of maximum temperature was 0.25 °C/year, 0.15 °C/year, 0.043 °C/year, and 0.082 °C/year respectively whereas the annual maximum significantly increasing trend was 0.083 °C/year. The results of MMK and Senʼs tests for temperature showed that significant increasing trends were observed in maximum temperature for the majority of the stations in the basin.

The mean annual streamflow computed throughout 1985 to 2018 was 149.48 m^3^/s (Gilgel Gibe at Abelti), 61.72 m^3^/s (Gojeb), 41.95 m^3^/s (Gilgel Gibe at Assendabo), 33.55 m^3^/s (Wabi), 9.85 m^3^/s (Guma), 8.37 m^3^/s (Bulbul), 4.97 m^3^/s (Gibe), 3.88 m^3^/s (Gecha), 2.91 m^3^/s (Megecha) and 0.39 m^3^/s (Bidru Awana). Coefficient of variation (CV) analysis of all gauging streamflow stations during the summer season streamflow showed extremely high variability (CV > 70). The number of years having negative SAI was more than years with positive SAI in annual and all seasons which indicates Upper Omo Gibe Basin experiences more dry years than wet years. According to the result of MMK significant increase in streamflow (P < 0.05) was observed at the Gibe gauging station (Z = 2.02) and Gilgel Gibe at Assendabo station (Z = 2.02) in the summer season. A significant decrease trend of streamflow was observed in the Bulbul gauging station in the spring season (Z = −2.20), Megecha gauging station in the summer season (Z = −2.19), and annual (Z = −2.34). In the summer season, the Gibe gauging station significantly increased trend at the magnitude of 0.2957 m^3^/s whereas the Gilgel Gibe at Assendabo station magnitude of 7.6566 m^3^/s and Megecha gauging station showed a significantly decreasing trend at the magnitude of 0.3226 m^3^/s. The Bulbul gauging station in the spring season showed a significantly decreasing trend in magnitude of 0.0148 m^3^/s and the Megecha gauging station annually showed a significantly decreasing trend magnitude of 0.0595 m^3^/s. In all seasons and annually the decreasing trend of the streamflow was greater than an increasing trend in the Upper Omo Gibe Basin. Statistically significant increasing and decreasing trends observed were 22 (27.5 %) for rainfall, 43 (53.75 %) for maximum temperature, 39 (48.75 %) for minimum temperature, and 5 (10 %) for streamflow with the MMK test. In the ITA method 30 (37.5 %) for rainfall, 47 (58.75 %) for maximum temperature, 44 (55 %) for minimum temperature, and 34 (68 %) for streamflow were obtained. The ITA method was more showed a significant increase and decrease than the MMK method. This indicated that the ITA method displays stronger significant trends than the MMK method. Overall, this finding offers valuable insights for environmentalists and the concerned bodies to make more informed decisions about future agricultural and water resources management in the face of hydroclimatic variability of the basin.

## CRediT authorship contribution statement

**Eyasu Tafese Mekuria:** Methodology, Investigation, Formal analysis, Data curation, Conceptualization. **Tamene Adugna Demissie:** Validation, Supervision, Software. **Fekadu Fufa Feyessa:** Writing – review & editing, Writing – original draft, Visualization.

## Ethical statement

This study did not involve any human or animal subjects. All data sources used in the research have been properly acknowledged. The authors confirm that this research is original, has not published elsewhere, and it is not under consideration by any other journal.

## Data availability statement

The data used in this study can be available from the corresponding authors Eyasu Tafese Mekuria (eyasu.tafese@wku.edu.et) on reasonable request.

## Funding statement

No funding was received for conducting this study.

## Declaration of competing interest

The authors declare that they have no known competing financial interests or personal relationships that could have appeared to influence the work reported in this paper.
