# Supplementary material for: Investigation of the spatial and temporal long-term hydro-climatic trends in Upper Omo Gibe Basin, Ethiopia
Source: Heliyon. 2025 Jan 27;11(3):e42265. doi: 10.1016/j.heliyon.2025.e42265 (PMC11830377; doi:10.1016/j.heliyon.2025.e42265)
Supplement: Multimedia component 1 [file mmc1.docx]

Table 1 List meteorological stations in the Upper Omo Gibe basin

| No | Stations | Elevation (m) | Longitude (E) | Latitude (N) | Period |
| --- | --- | --- | --- | --- | --- |
| 1 | Assendabo | 1764 | 37.23 | 7.76 | 1981-2022 |
| 2 | Bele | 1240 | 37.53 | 6.92 | 1981-2022 |
| 3 | Bonga | 1599 | 36.24 | 7.28 | 1981-2022 |
| 4 | Chida | 1649 | 36.79 | 7.17 | 1981-2022 |
| 5 | Chira | 2105 | 36.25 | 7.75 | 1981-2022 |
| 6 | Dedo | 2281 | 36.88 | 7.50 | 1981-2022 |
| 7 | Deri Goma | 1746 | 36.29 | 7.38 | 1981-2022 |
| 8 | Gedo | 2520 | 37.46 | 9.02 | 1981-2022 |
| 9 | Hosaina | 2306 | 37.86 | 7.57 | 1981-2022 |
| 10 | Jimma | 1710 | 36.82 | 7.67 | 1981-2022 |
| 11 | Limu Genet | 1767 | 36.96 | 8.08 | 1981-2022 |
| 12 | Sekoru | 1937 | 37.43 | 7.93 | 1981-2022 |
| 13 | Shebe | 1921 | 36.52 | 7.51 | 1981-2022 |
| 14 | Wolayita | 1854 | 37.75 | 6.82 | 1981-2022 |
| 15 | Woliso | 2058 | 37.98 | 8.55 | 1981-2022 |
| 16 | Wolkite | 1884 | 37.77 | 8.28 | 1981-2022 |

Table 2 List of streamflow gauging stations in the Upper Omo Gibe basin

| No | Stations | Longitude | Latitude | Period |
| --- | --- | --- | --- | --- |
| 1 | Bidru Awana at Sekoru | 37.24 | 7.0.55 | 1985-2018 |
| 2 | Bulbul at Serbo | 37.02 | 7.34 | 1985-2018 |
| 3 | Gecha at Bonga | 36.13 | 7.17 | 1985-2018 |
| 4 | Ghibe at Seka | 37.35 | 8.14 | 1985-2018 |
| 5 | Gilgel Gibe at Abelti | 37.61 | 8.16 | 1985-2018 |
| 6 | Gilgel Gibe at Assendabo | 37.18 | 7.75 | 1985-2018 |
| 7 | Gojeb at Shebe | 36.23 | 7.25 | 1985-2018 |
| 8 | Guma at Anidiracha | 36.25 | 7.15 | 1985-2018 |
| 9 | Megecha at Gubire | 37.80 | 8.19 | 1985-2018 |
| 10 | Wabi at Wolkite | 37.48 | 8.11 | 1985-2018 |

Table 3 Annual and seasonal rainfall of Upper Omo Gibe Basin

| No | Stations | Annual | Autumn | Spring | Summer | Winter |
| --- | --- | --- | --- | --- | --- | --- |
| 1 | Assendabo | 1309.25 | 283.59 | 349.64 | 593.63 | 82.39 |
| 2 | Bele | 1211.20 | 297.20 | 333.81 | 485.45 | 94.74 |
| 3 | Bonga | 1814.81 | 467.15 | 526.35 | 658.20 | 163.10 |
| 4 | Chida | 1529.09 | 358.25 | 519.34 | 505.57 | 145.93 |
| 5 | Chira | 1863.52 | 465.15 | 503.28 | 737.82 | 157.26 |
| 6 | Dedo | 1689.92 | 372.03 | 452.03 | 757.72 | 108.14 |
| 7 | Deri Goma | 1601.93 | 368.66 | 475.33 | 620.01 | 137.93 |
| 8 | Gedo | 1037.04 | 189.85 | 222.66 | 571.19 | 53.34 |
| 9 | Hosaina | 1212.83 | 261.44 | 393.57 | 458.11 | 99.71 |
| 10 | Jimma | 1607.27 | 395.47 | 432.53 | 664.18 | 115.09 |
| 11 | Limu Genet | 2056.23 | 535.55 | 474.80 | 957.22 | 88.66 |
| 12 | Sekoru | 1408.79 | 292.62 | 355.26 | 677.12 | 83.79 |
| 13 | Shebe | 1649.98 | 409.45 | 456.57 | 654.93 | 129.02 |
| 14 | Wolayita | 1347.77 | 295.87 | 422.47 | 516.37 | 113.07 |
| 15 | Woliso | 1276.40 | 229.53 | 261.57 | 735.01 | 50.29 |
| 16 | Wolkite | 1259.87 | 217.68 | 290.09 | 685.68 | 66.42 |

Table 4 Annual and seasonal average maximum temperature of Upper Omo Gibe Basin

| No | Station | Annual | Autumn | Spring | Summer | Winter |
| --- | --- | --- | --- | --- | --- | --- |
| 1 | Assendabo | 27.27 | 26.66 | 28.48 | 25.15 | 28.79 |
| 2 | Bele | 29.93 | 29.37 | 30.60 | 27.92 | 31.83 |
| 3 | Bonga | 26.92 | 27.01 | 27.36 | 25.41 | 27.93 |
| 4 | Chida | 27.02 | 26.45 | 27.73 | 25.01 | 28.95 |
| 5 | Chira | 23.94 | 23.58 | 24.99 | 21.93 | 25.28 |
| 6 | Dedo | 23.58 | 23.22 | 24.29 | 22.36 | 24.47 |
| 7 | Deri Goma | 26.29 | 25.62 | 27.48 | 24.85 | 27.23 |
| 8 | Gedo | 22.30 | 21.82 | 23.67 | 20.10 | 23.63 |
| 9 | Hosaina | 22.78 | 22.45 | 23.91 | 20.63 | 24.20 |
| 10 | Jimma | 27.59 | 27.00 | 29.01 | 25.46 | 28.93 |
| 11 | Limu Genet | 27.18 | 26.30 | 28.99 | 24.58 | 28.92 |
| 12 | Sekoru | 26.30 | 25.51 | 28.00 | 23.86 | 27.89 |
| 13 | Shebe | 25.92 | 25.27 | 27.10 | 23.88 | 27.48 |
| 14 | Wolayita | 25.40 | 25.04 | 26.56 | 22.62 | 27.43 |
| 15 | Woliso | 25.19 | 24.70 | 27.26 | 22.22 | 26.65 |
| 16 | Wolkite | 27.60 | 27.19 | 29.14 | 25.36 | 28.75 |

Table 5 Annual and seasonal average minimum temperature of Upper Omo Gibe basin

| No | Station | Annual | Autumn | Spring | Summer | Winter |
| --- | --- | --- | --- | --- | --- | --- |
| 1 | Assendabo | 11.41 | 10.55 | 12.80 | 12.44 | 9.86 |
| 2 | Bele | 17.56 | 17.09 | 18.19 | 17.63 | 17.37 |
| 3 | Bonga | 11.88 | 11.91 | 12.54 | 12.63 | 10.33 |
| 4 | Chida | 14.91 | 14.42 | 15.47 | 14.69 | 15.08 |
| 5 | Chira | 12.06 | 11.80 | 12.61 | 12.10 | 11.75 |
| 6 | Dedo | 11.46 | 11.28 | 11.78 | 11.31 | 11.46 |
| 7 | Deri Goma | 13.82 | 13.75 | 13.96 | 14.01 | 13.56 |
| 8 | Gedo | 10.04 | 9.73 | 10.61 | 9.79 | 10.04 |
| 9 | Hosaina | 11.03 | 10.75 | 11.94 | 11.48 | 9.93 |
| 10 | Jimma | 11.70 | 11.61 | 12.83 | 13.70 | 8.92 |
| 11 | Limu Genet | 13.61 | 12.99 | 14.68 | 14.18 | 12.58 |
| 12 | Sekoru | 13.42 | 12.76 | 14.47 | 13.38 | 13.07 |
| 13 | Shebe | 13.50 | 13.07 | 13.98 | 13.33 | 13.64 |
| 14 | Wolayita | 14.64 | 14.41 | 15.35 | 14.24 | 14.59 |
| 15 | Woliso | 13.05 | 12.37 | 13.92 | 12.71 | 13.22 |
| 16 | Wolkite | 13.51 | 13.33 | 13.82 | 13.81 | 13.09 |

Table 6 Annual and seasonal average streamflow of Upper Omo Gibe Basin

| No | Gauging station | Spring | Summer | Autumn | Winter | Annual |
| --- | --- | --- | --- | --- | --- | --- |
| 1 | Bidru Awana | 0.16 | 0.79 | 0.47 | 0.12 | 0.39 |
| 2 | Bulbul | 1.09 | 17.10 | 14.13 | 0.98 | 8.37 |
| 3 | Gecha | 2.03 | 6.95 | 5.35 | 1.11 | 3.88 |
| 4 | Ghibe | 2.23 | 8.94 | 6.99 | 1.66 | 4.97 |
| 5 | Gilgel Gibe Abelti | 31.83 | 271.45 | 243.31 | 48.28 | 149.48 |
| 6 | Gilgel Gibe Assendabo | 10.63 | 84.79 | 64.20 | 7.30 | 41.95 |
| 7 | Gojeb | 23.06 | 110.99 | 96.05 | 15.58 | 61.72 |
| 8 | Guma | 5.32 | 19.29 | 12.56 | 1.99 | 9.85 |
| 9 | Megecha | 1.01 | 6.93 | 3.26 | 0.34 | 2.91 |
| 10 | Wabi | 9.18 | 89.40 | 31.29 | 3.24 | 33.55 |

Table 7 Homogeneity test statistics results of rainfall

| No | Stations | P value of the test statistics | | | |
| --- | --- | --- | --- | --- | --- |
|  |  | Pittett test | SHNT | BR-test | VNT |
| 1 | Assendabo | 0.067 | 0.27 | 0.089 | 0.23 |
| 2 | Bele | 0.549 | 0.909 | 0.720 | 0.130 |
| 3 | Bonga | 0.34 | 0.067 | 0.42 | 0.12 |
| 4 | Chida | 0.621 | 0.077 | 0.284 | 0.154 |
| 5 | Chira | 0.976 | 0.321 | 0.538 | 0.397 |
| 6 | Dedo | 0.11 | **0.0099** | **0.0076** | **0.032** |
| 7 | Deri Goma | **0.033** | **0.032** | 0.066 | **0.023** |
| 8 | Gedo | 0.076 | **0.01** | 0.43 | **0.0056** |
| 9 | Hosaina | 0.854 | 0.247 | 0.527 | 0.373 |
| 10 | Jimma | 0.101 | 0.056 | 0.15 | 0.35 |
| 11 | Limu Genet | 0.22 | **0.0087** | **0.01** | **0.04** |
| 12 | Sekoru | 0.758 | 0.665 | 0.419 | 0.167 |
| 13 | Shebe | 0.431 | 0.222 | 0.463 | 0.131 |
| 14 | Wolayita | 0.474 | 0.100 | 0.246 | 0.332 |
| 15 | Woliso | 0.604 | 0.051 | 0.359 | 0.160 |
| 16 | Wolkite | 0.098 | 0.063 | 0.077 | 0.088 |

Table 8 Homogeneity test statistics results of maximum temperature

| No | Stations | P value of the test statistics | | | |
| --- | --- | --- | --- | --- | --- |
|  |  | Pittett test | SHNT | BR-test | VNT |
| 1 | Assendabo | 0.065 | 0.087 | 0.098 | 0.656 |
| 2 | Bele | 0.056 | 0.069 | 0.085 | 0.061 |
| 3 | Bonga | 0.644 | 0.315 | 0.439 | 0.397 |
| 4 | Chida | 0.24 | 0.180 | 0.337 | 0.118 |
| 5 | Chira | **0.001** | **0.032** | **0.0001** | **0.002** |
| 6 | Dedo | **0.01** | **0.031** | **0.0056** | **0.0006** |
| 7 | Deri Goma | **0.0001** | **0.000** | **0.0001** | **0.0001** |
| 8 | Gedo | 0.134 | 0.089 | 0.059 | 0.087 |
| 9 | Hosaina | 0.078 | 0.191 | 0.075 | 0.092 |
| 10 | Jimma | 0.321 | 0.123 | 0.54 | 0.34 |
| 11 | Limu Genet | 0.063 | 0.074 | 0.089 | 0.097 |
| 12 | Sekoru | 0.076 | 0.054 | 0.065 | 0.089 |
| 13 | Shebe | 0.099 | 0.22 | 0.098 | 0.45 |
| 14 | Wolayita | 0.118 | 0.109 | 0.017 | 0.389 |
| 15 | Woliso | 0.233 | 0.134 | 0.65 | 0.732 |
| 16 | Wolkite | 0.32 | 0.12 | 0.254 | 0.321 |

Table 9 Homogeneity test statistics results of minimum temperature

| No | Stations | P value of the test statistics | | | |
| --- | --- | --- | --- | --- | --- |
|  |  | Pittett test | SHNT | BR-test | VNT |
| 1 | Assendabo | 0.230 | 0.131 | 0.324 | 0.087 |
| 2 | Bele | 0.594 | 0.377 | 0.703 | 0.342 |
| 3 | Bonga | 0.474 | 0.232 | 0.144 | 0.055 |
| 4 | Chida | 0.920 | 0.057 | **0.021** | **0.002** |
| 5 | Chira | **0.0001** | 0.052 | **0.000** | **0.000** |
| 6 | Dedo | 0.067 | **0.007** | **0.005** | **0.0001** |
| 7 | Deri Goma | **0.024** | 0.387 | 0.149 | 0.084 |
| 8 | Gedo | 0.093 | 0.343 | 0.123 | 0.432 |
| 9 | Hosaina | 0.052 | 0.635 | 0.782 | 0.057 |
| 10 | Jimma | 0.056 | 0.071 | 0.068 | 0.077 |
| 11 | Limu Genet | 0.179 | 0.058 | 0.166 | 0.098 |
| 12 | Sekoru | 0.056 | 0.231 | 0.111 | 0.303 |
| 13 | Shebe | 0.787 | 0.467 | 0.292 | 0.407 |
| 14 | Wolayita | 0.055 | 0.066 | 0.061 | 0.079 |
| 15 | Woliso | 0.189 | 0.099 | 0.065 | 0.067 |
| 16 | Wolkite | 0.161 | 0.261 | 0.234 | 0.923 |

Table 10 Homogeneity test statistics results of streamflow

| No | Gauging Stations | P value of the test statistics | | | |
| --- | --- | --- | --- | --- | --- |
|  |  | Pittett test | SHNT | BR-test | VNT |
| 1 | Bidru Awana | 0.083 | 0.073 | 0.072 | 0.065 |
| 2 | Bulbul | 0.307 | 0.722 | 0.437 | 0.038 |
| 3 | Gecha | 0.088 | 0.128 | 0.094 | 0.147 |
| 4 | Ghibe | **0.01** | **0.01** | **0.01** | **0.01** |
| 5 | Gilgelgibe Abelti | 0.289 | 0.328 | 0.140 | 0.051 |
| 6 | Gilgelgibe Assendabo | 0.559 | 0.172 | 0.281 | 0.272 |
| 7 | Gojeb | 0.987 | 0.504 | 0.431 | 0.219 |
| 8 | Guma | **0.0008** | **0.0005** | **0.008** | **0.03** |
| 9 | Megech | 0.054 | 0.076 | 0.084 | 0.770 |
| 10 | Wabi | 0.641 | 0.796 | 0.656 | 0.309 |

(A)

| 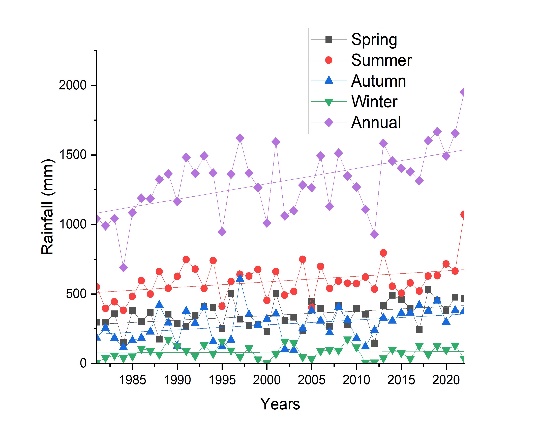  (C) | 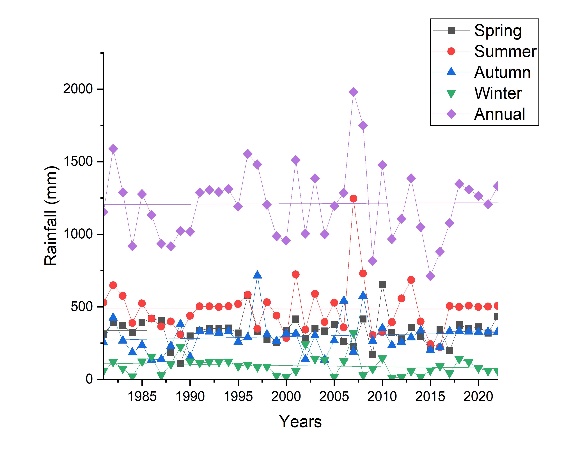  (B)  (D) |
| --- | --- |
| 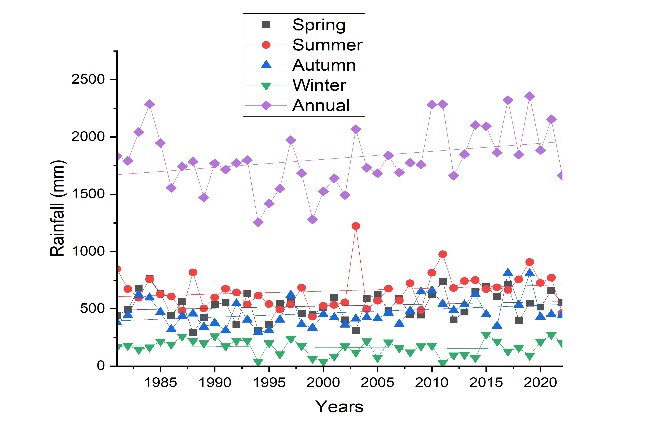 | 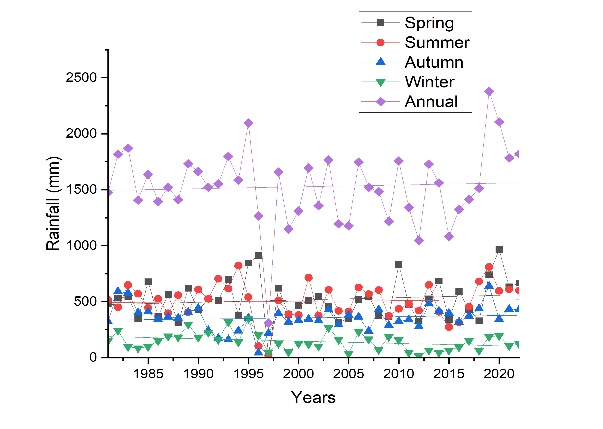 |
| 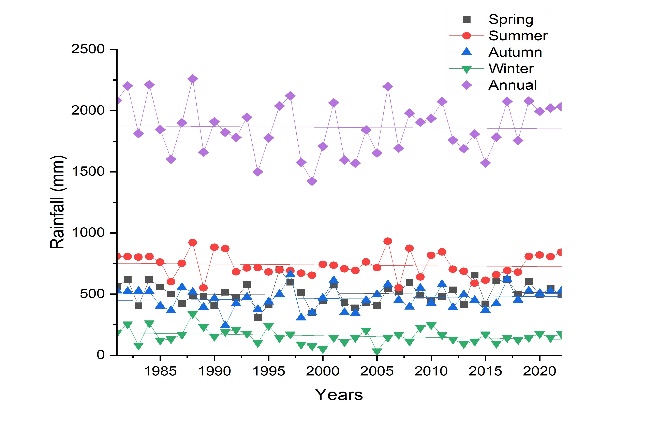  (G)  (E) | 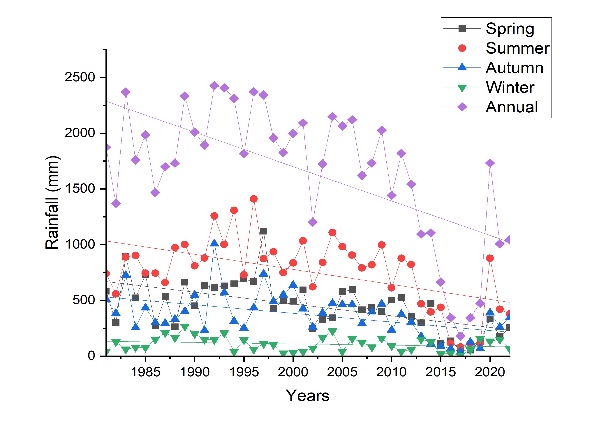  (H) |
| 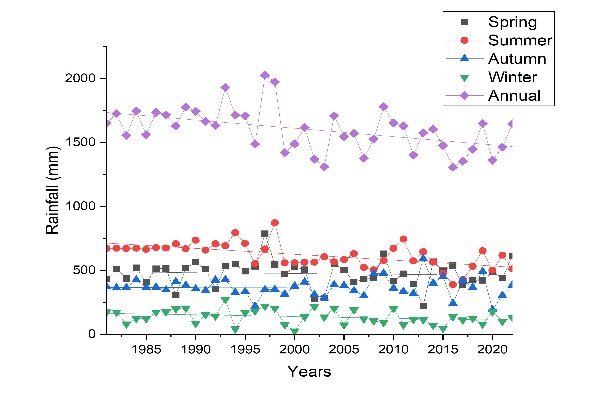 | 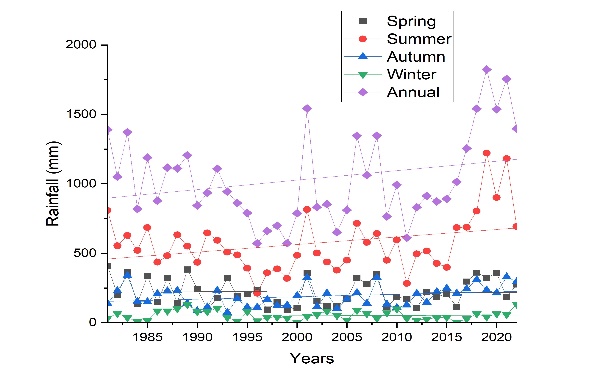 |

(A) Assendabo (B) Bele (C) Bonga (D) Chida (E) Chira (F) Dedo (G) Deri goma (H) Gedo stations

(F)

| 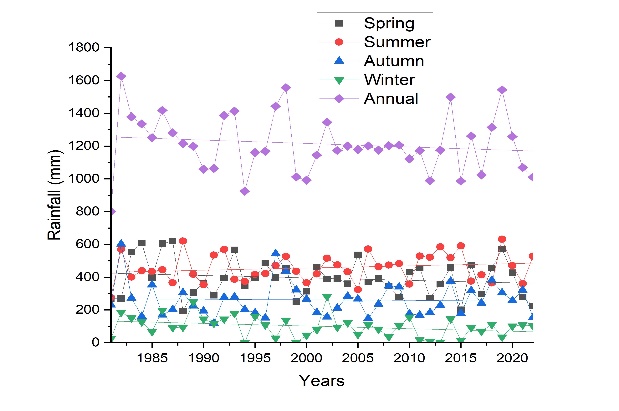 | 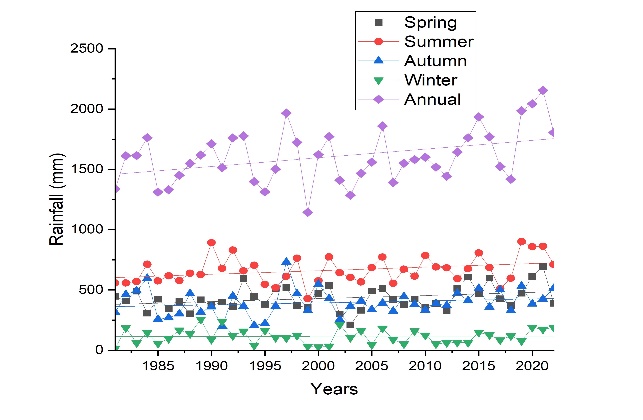 |
| --- | --- |
| 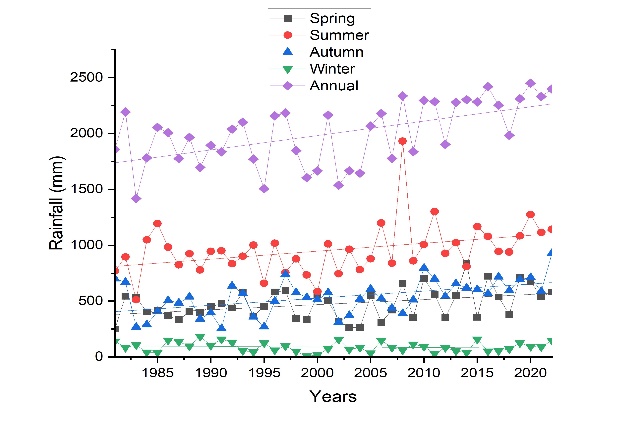  (K) | 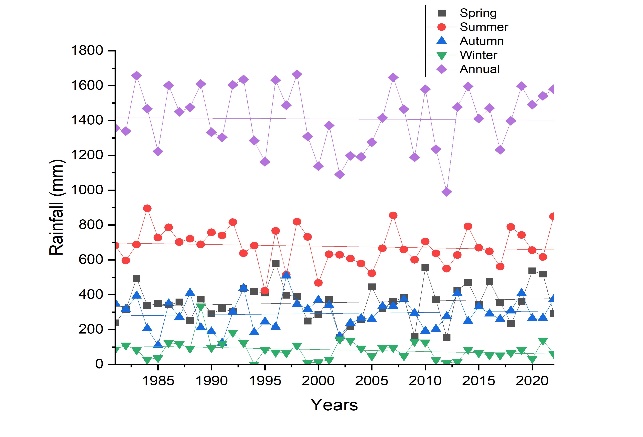  (L) |
| 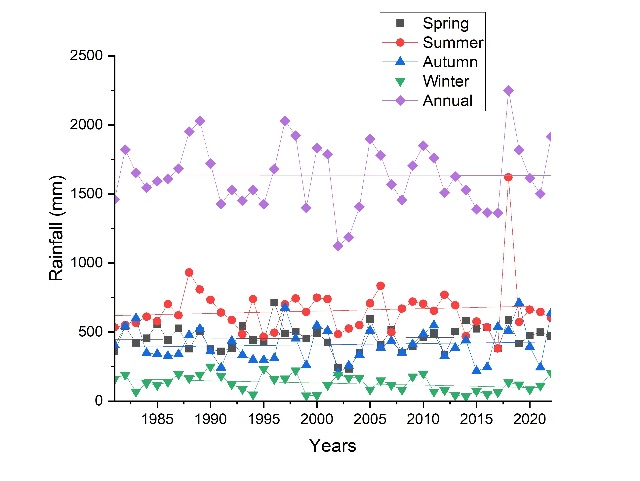  (O)  (M) | 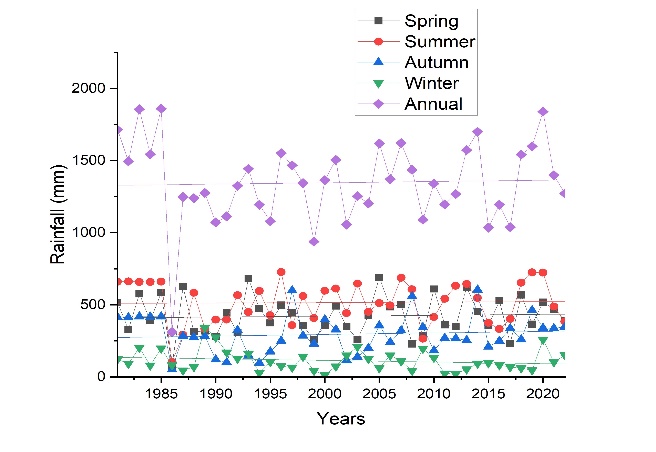  (P)  (N) |
| 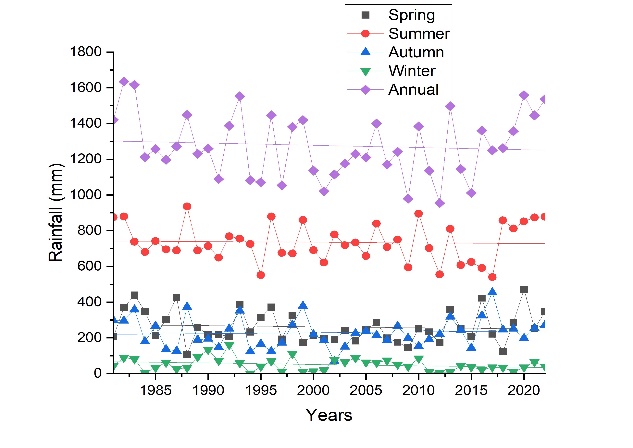 | 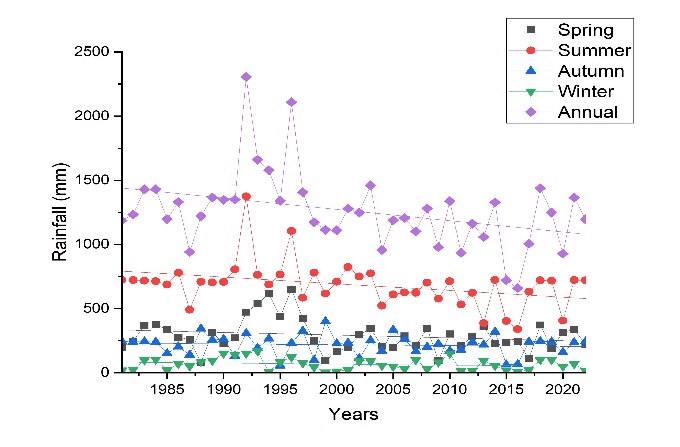 |

(I) Hosaina (J) Jimma (K) Limugenet (L) Sekoru (M) Shebe ( N) Wolayita (O) Woliso (P) Wolkite Rainfall

(I)

(J)

Figure 1 Time series plot for annual and seasonal rainfall stations

Figure 2 Result of ITA for spring rainfall

Figure 3 Result of ITA for summer rainfall

Figure 4 Result of ITA for autumn rainfall

Figure 5 Result of ITA for winter rainfall

Figure 6 Result of ITA for annual rainfall

| 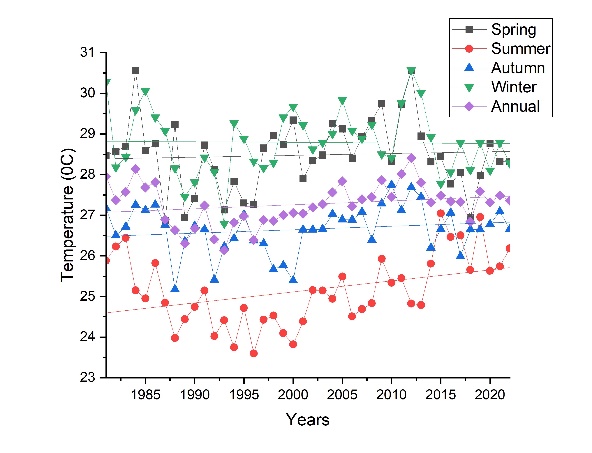 | 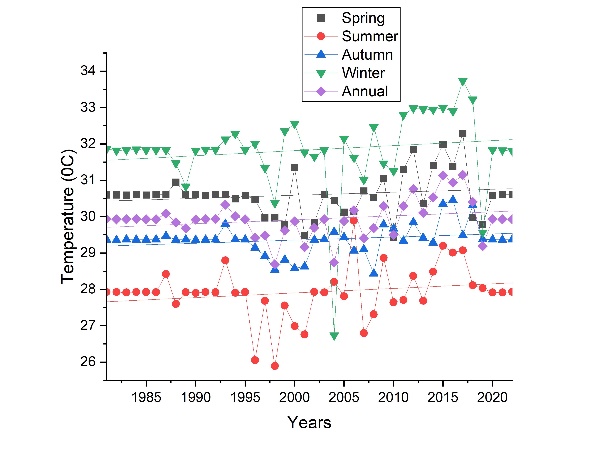  (B) |
| --- | --- |
| 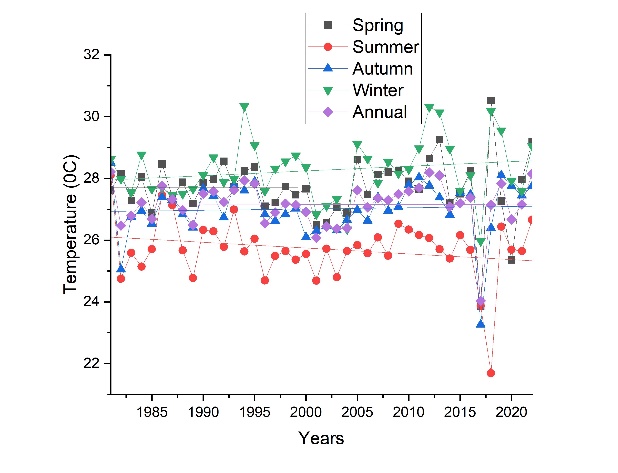  (C) | 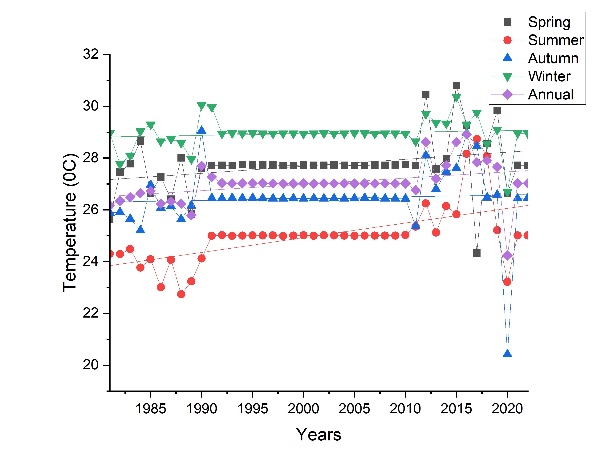  (D) |
| 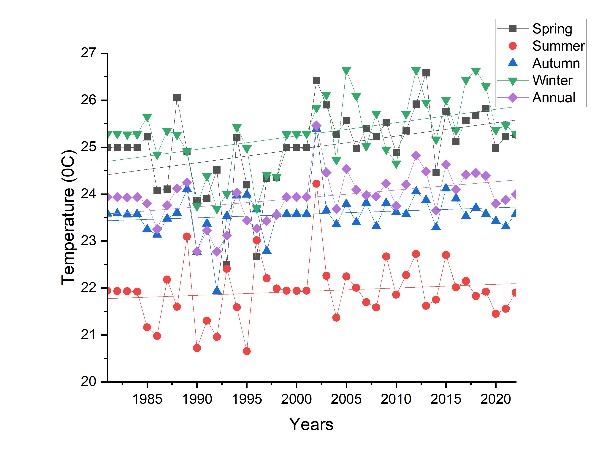  (E) | 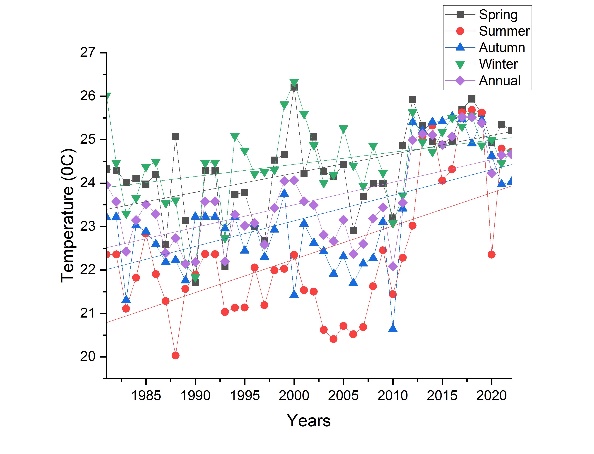  (H)  (F) |
| 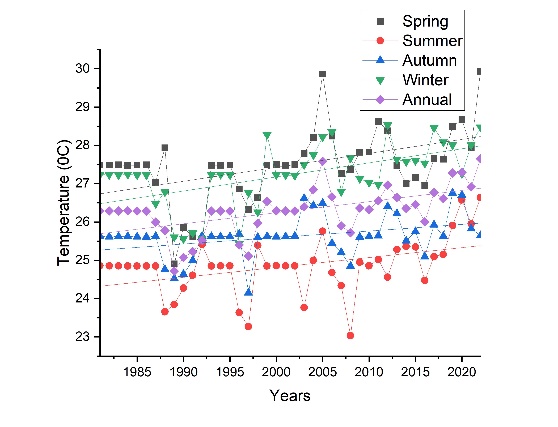  (G) | 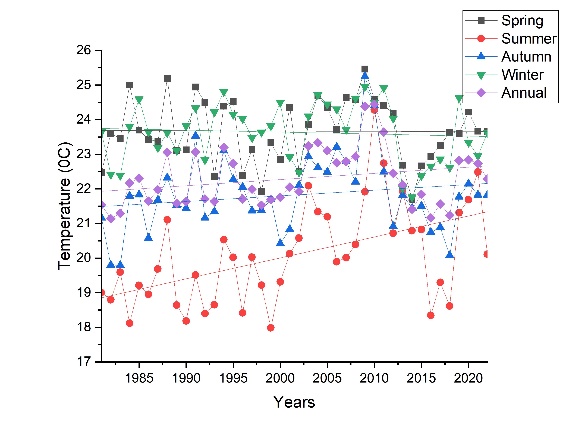 |

(A) Assendabo (B) Bele (C) Bonga (D) Chida (E) Chira (F) Dedo (G) Deri Goma (H) Gedo stations

(A)

| 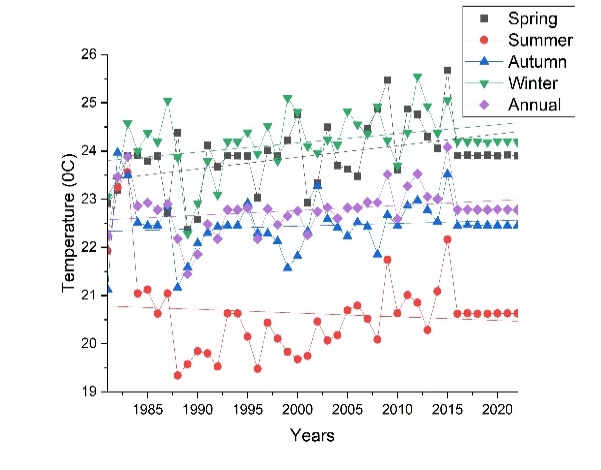 | 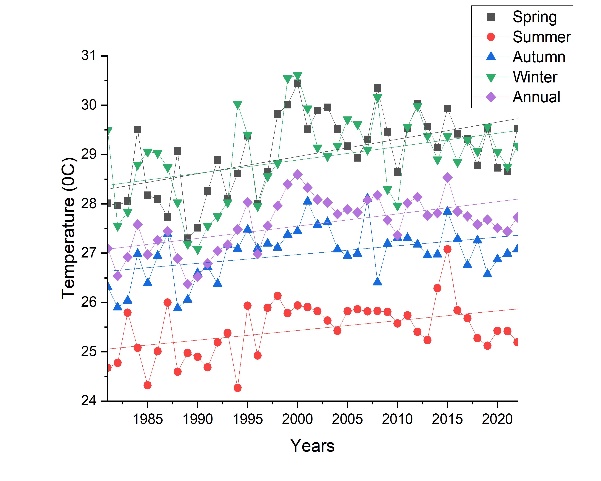 |
| --- | --- |
| 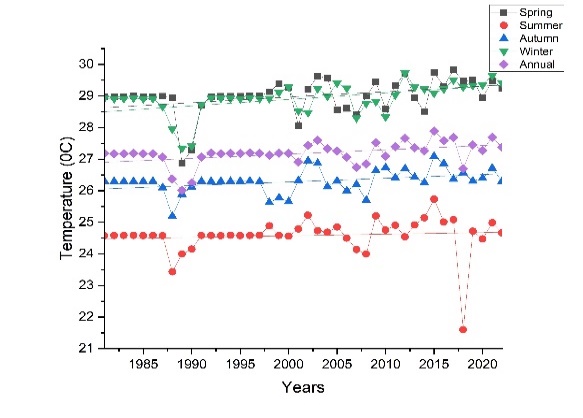  (K) | 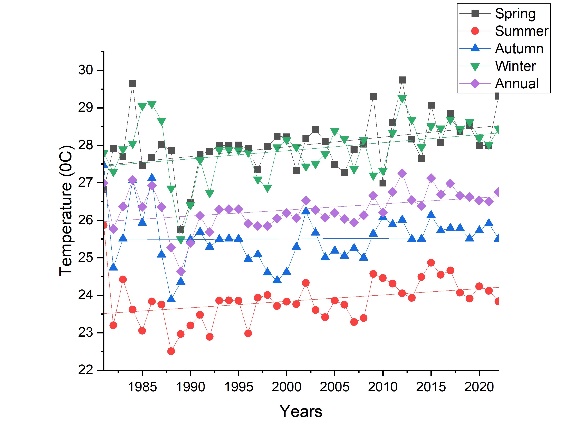  (N)  (L) |
| 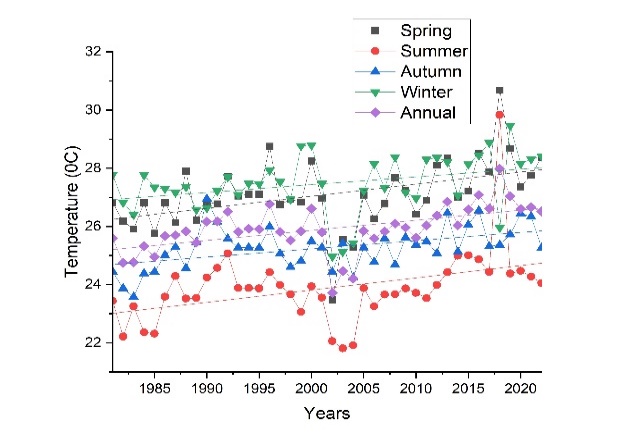  (M) | 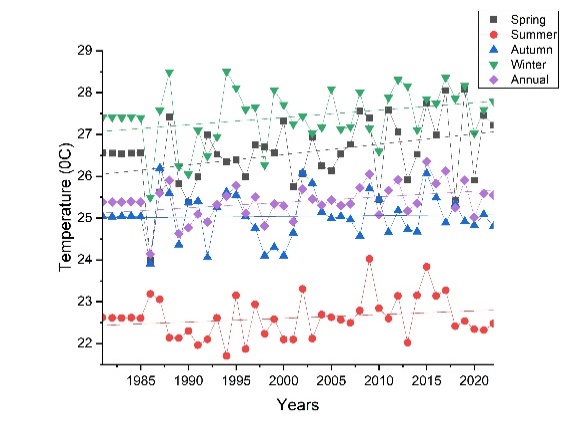 |
| 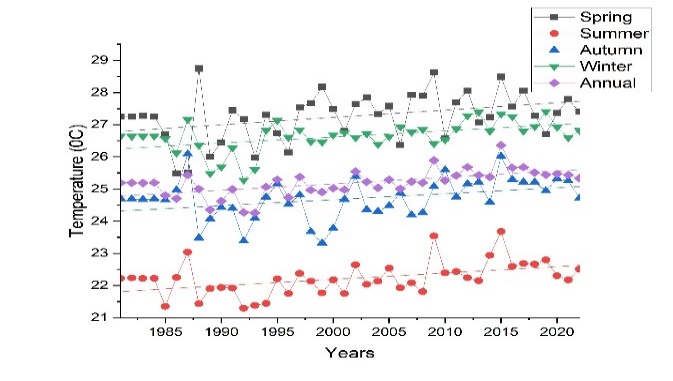  (O) | 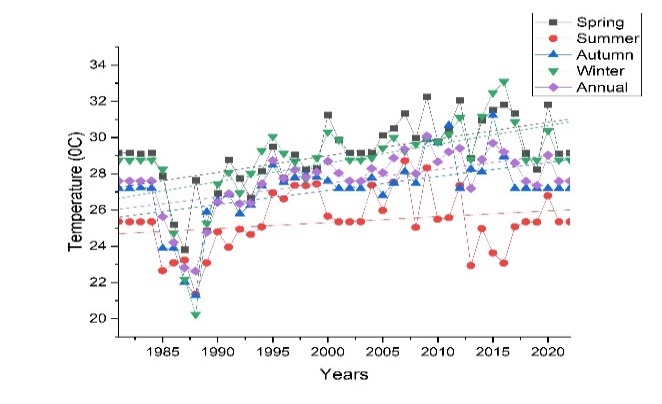  (P) |

(I) Hosaina (J) Jimma (K) Limugenet (L) Sekoru (M) Shebe ( N) Wolayita (O) Woliso (P) Wolkite

(J)

(I)

Figure 7 Time series plot of annual and seasonal maximum temperature

| 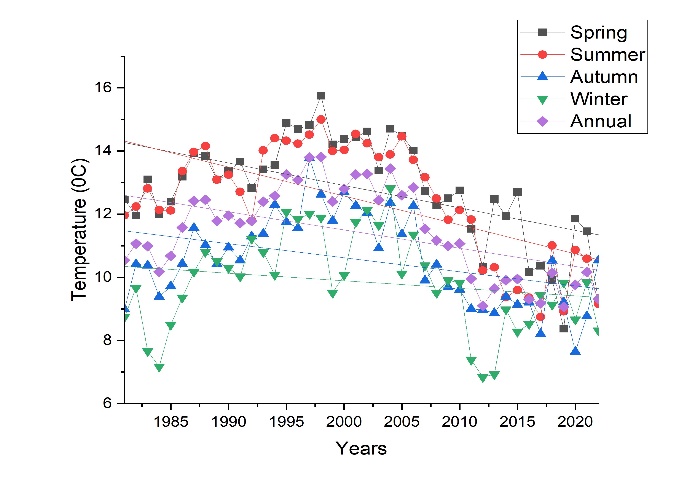  (A) | 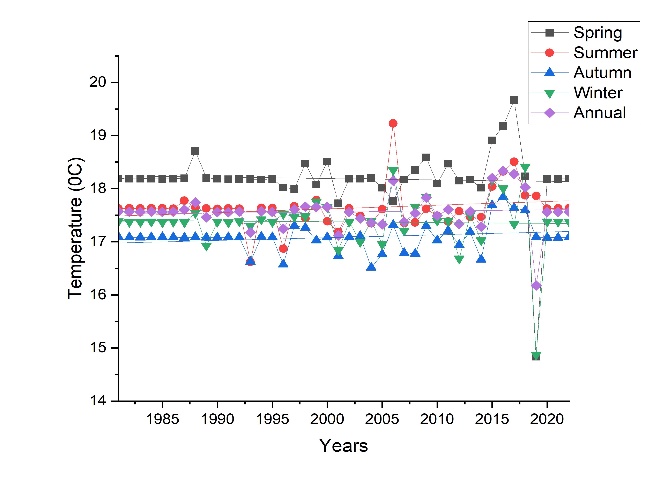  (B) |
| --- | --- |
| 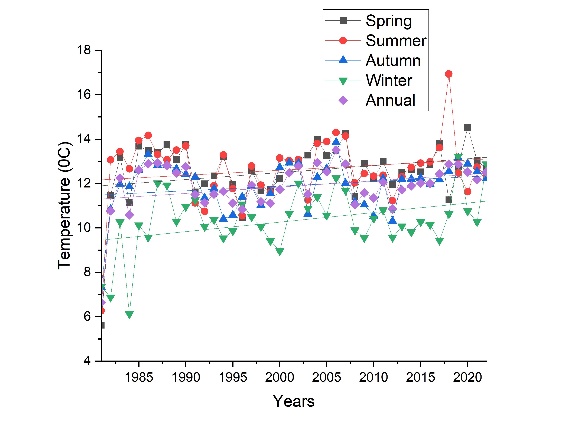  (C) | 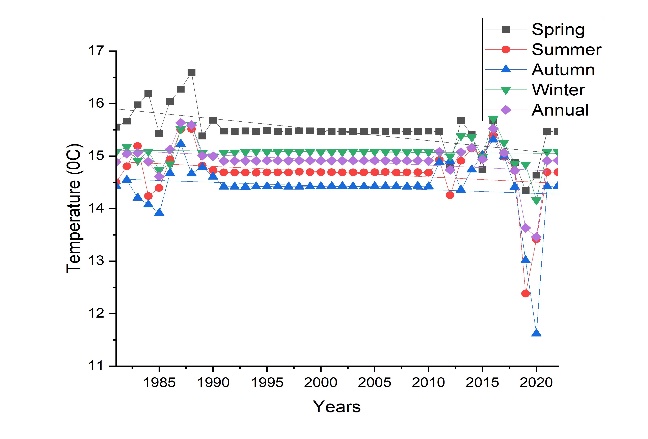  (D) |
| 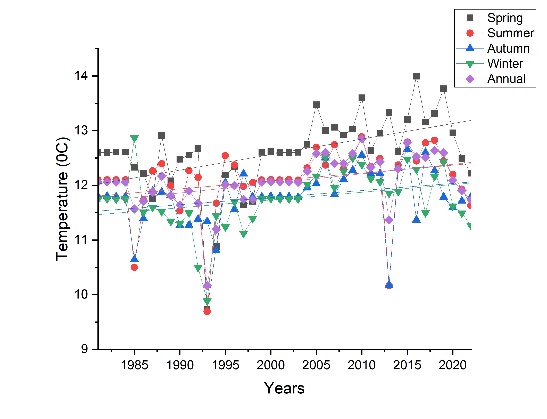  (E) | 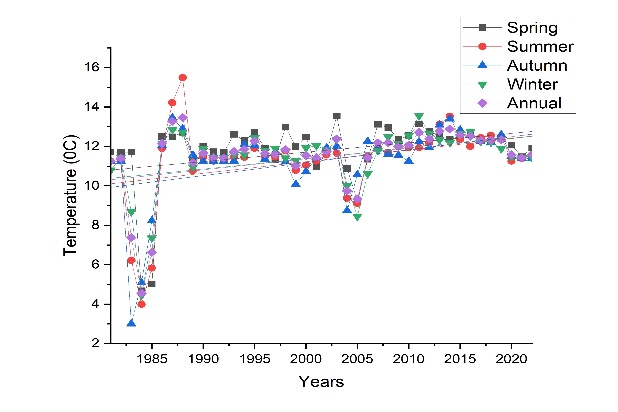  (F) |
| 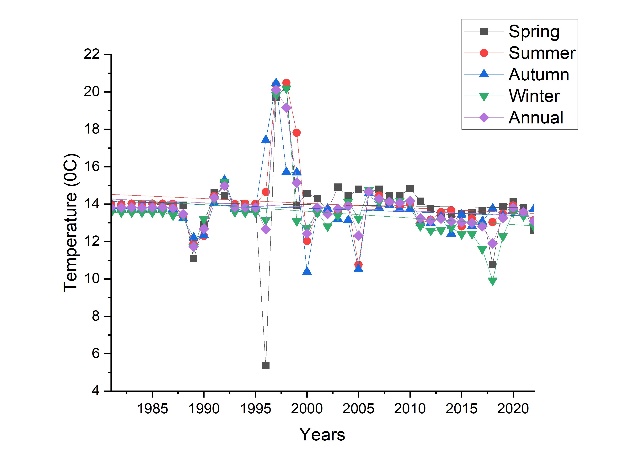  (G) | 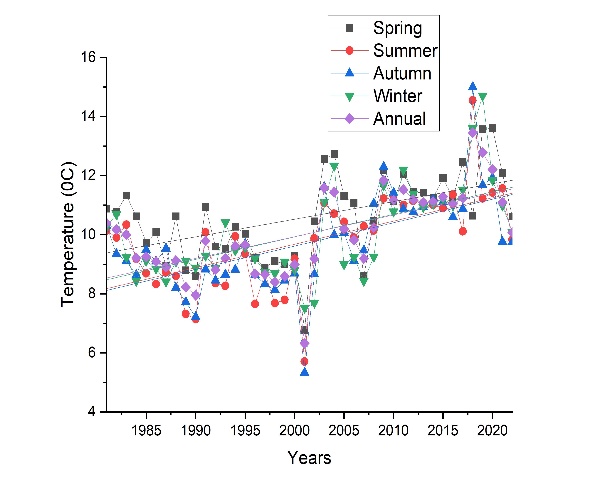  (H) |

(A) Assendabo (B) Bele (C) Bonga (D) Chida (E) Chira (F) Dedo (G) Deri Goma (H) Gedo stations

(I)

| 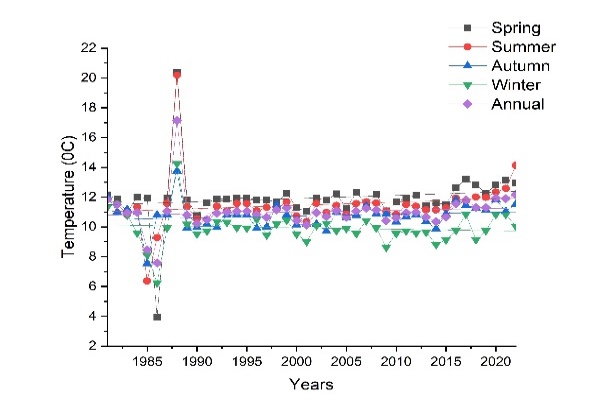 | 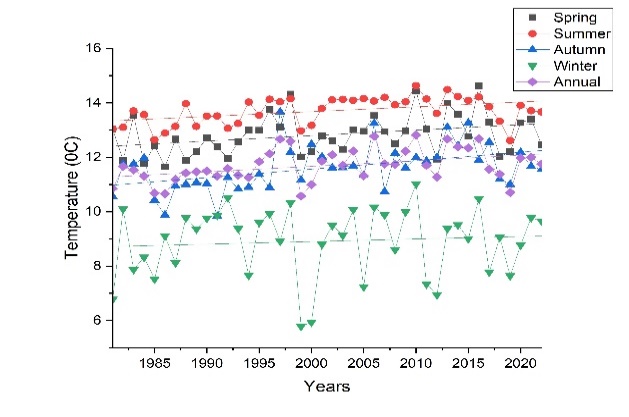  (J) |
| --- | --- |
| 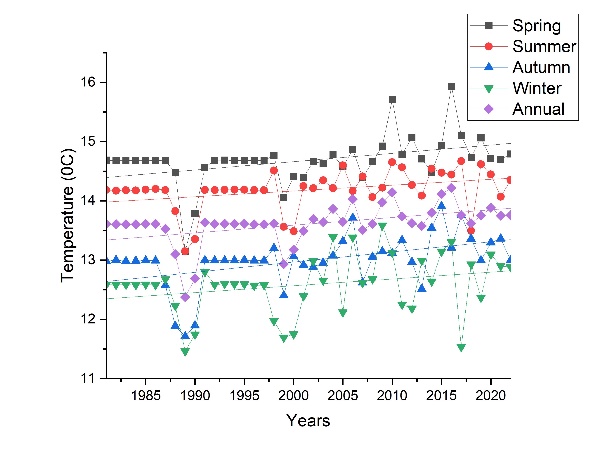  (K) | 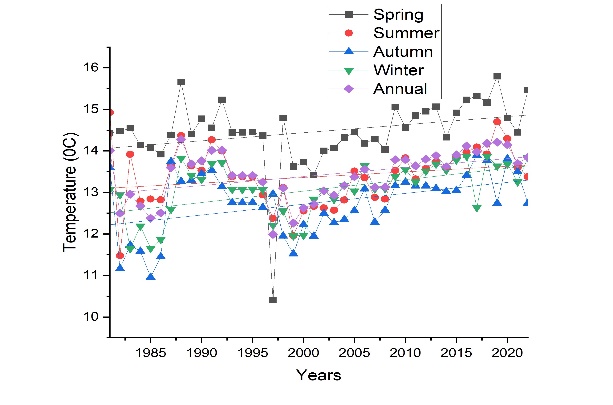  (L) |
| 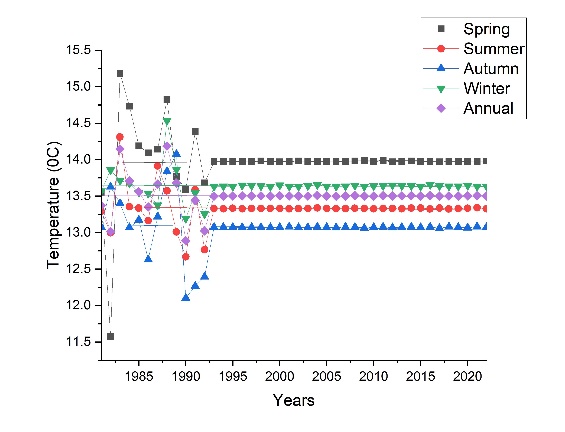  (M) | 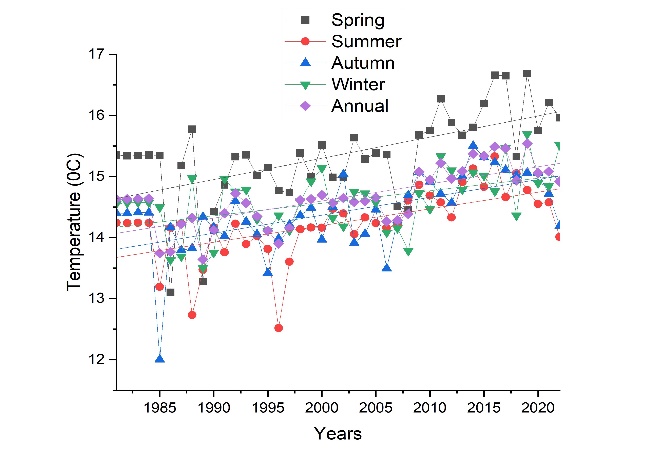  (N) |
| 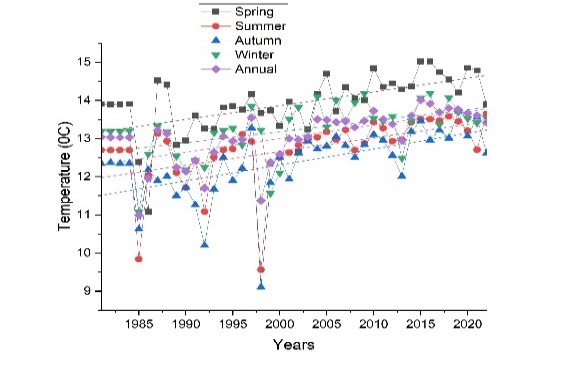  (O) | 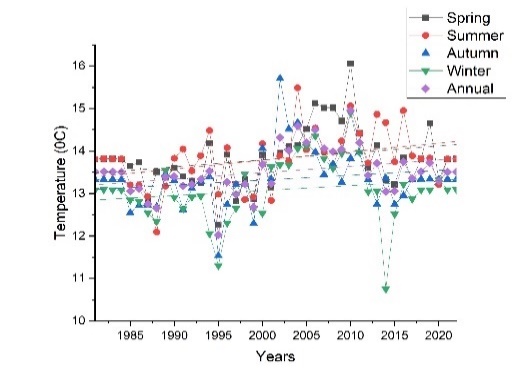  (P) |

(I) Hosaina (J) Jimma (K) Limugenet (L) Sekoru (M) Shebe ( N) Wolayita (O) Woliso (P) Wolkite

Figure 8 Time series plot of annual and seasonal minimum temperature

Figure 9 Result of ITA for spring maximum temperature

Figure 10 Result of ITA for summer maximum temperature

Figure 11 Result of ITA for autumn maximum temperature

Figure 12 Result of ITA for winter maximum temperature

Figure 13 Result of ITA for Annual maximum temperature

Figure 14 Result of ITA for spring minimum temperature

Figure 15 Result of ITA for summer minimum temperature

Figure 16 Result of ITA for autumn minimum temperature

Figure 17 Result of ITA for winter minimum temperature

Figure 18 Result of ITA for annual minimum temperature

| 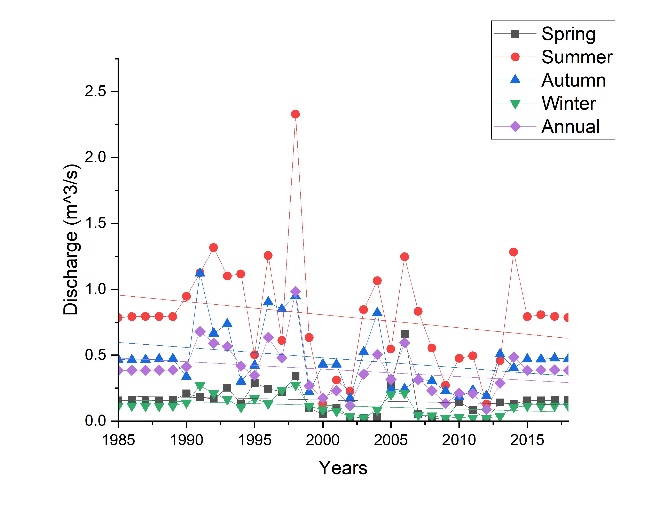  (A) | 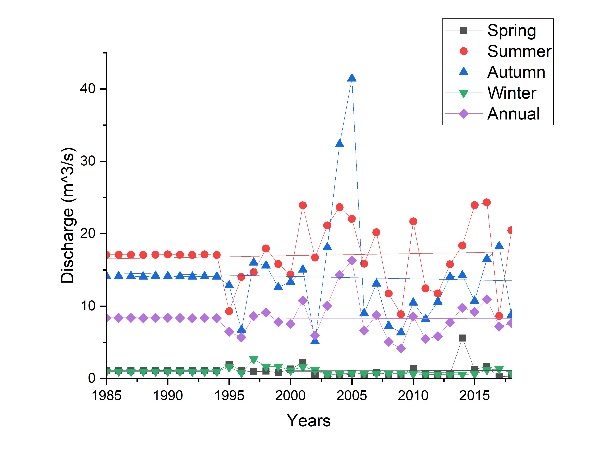  (B) |
| --- | --- |
| 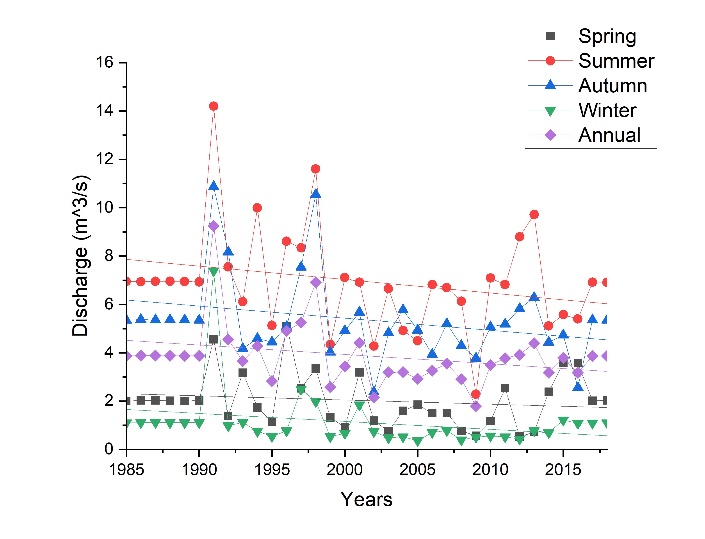  (C) | 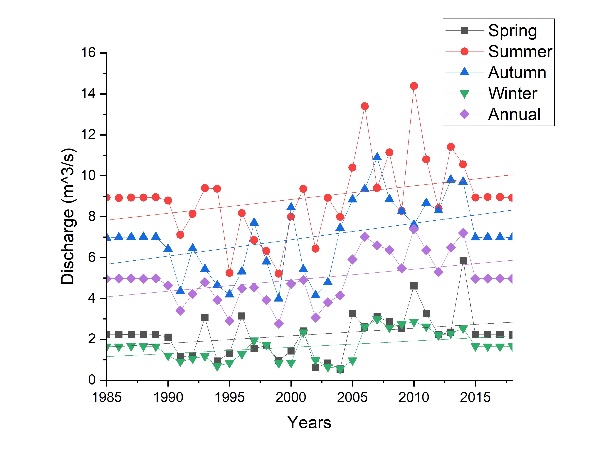  (D) |
| 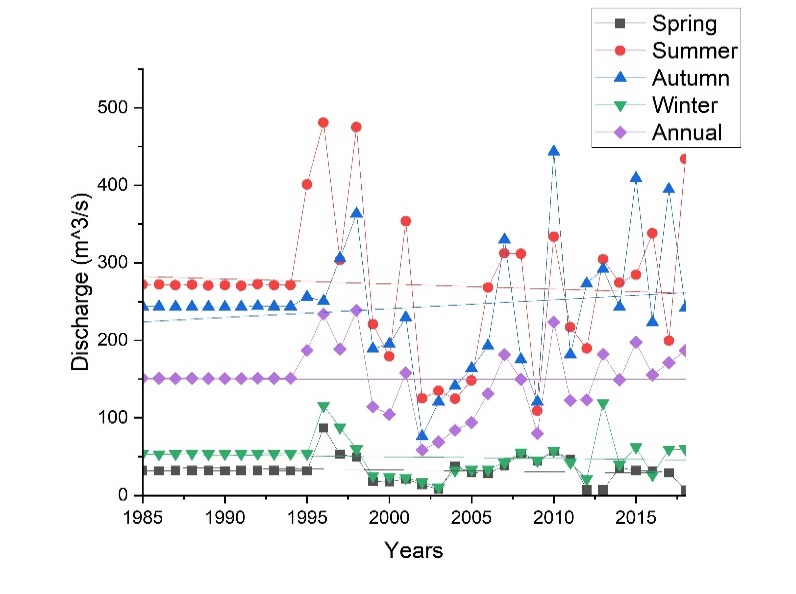  (E) | 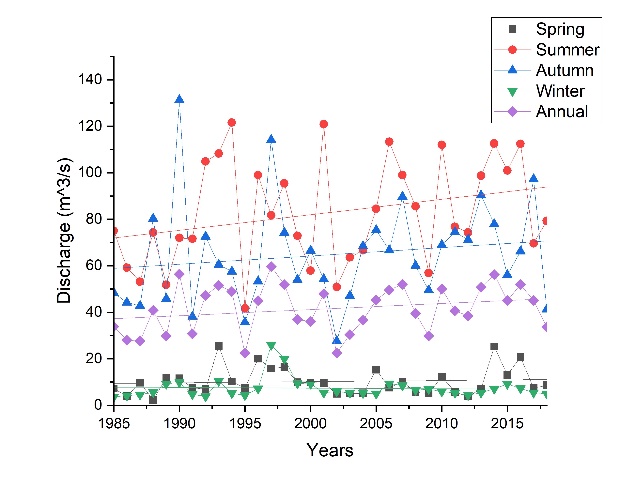  (F) |

(A) Bidru Awana (B) Bulbul (C) Gecha (D) Gibe (E) Gilgegibe Abelti (F) Gilgegibe Assendabo

| 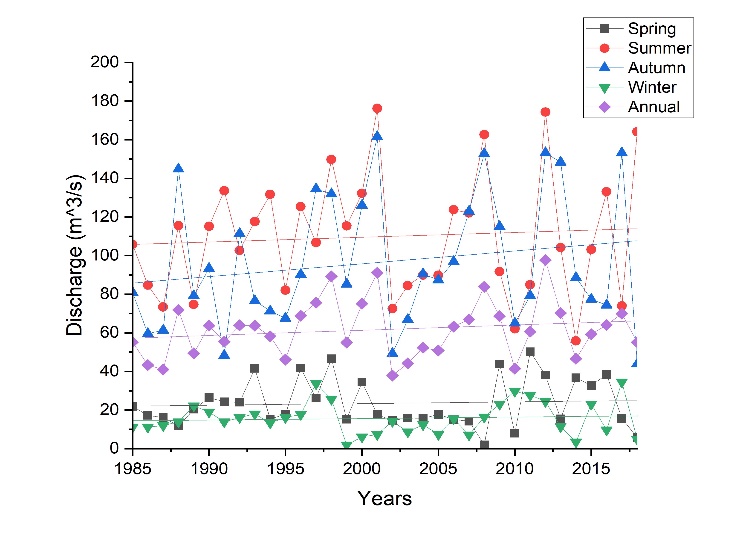  (G) | 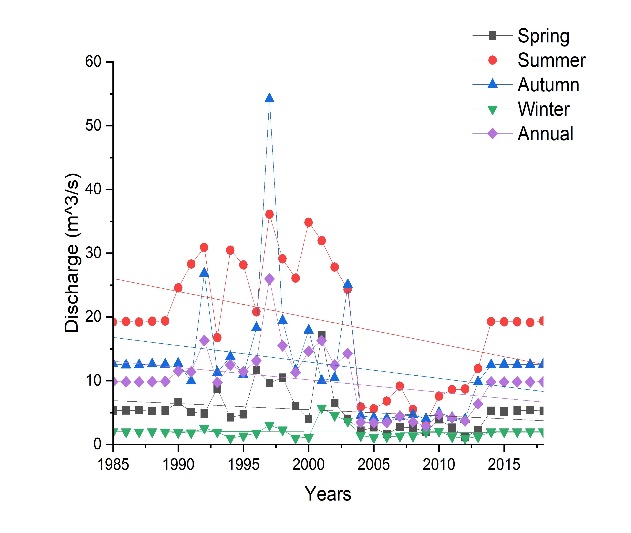  (H) |
| --- | --- |
| 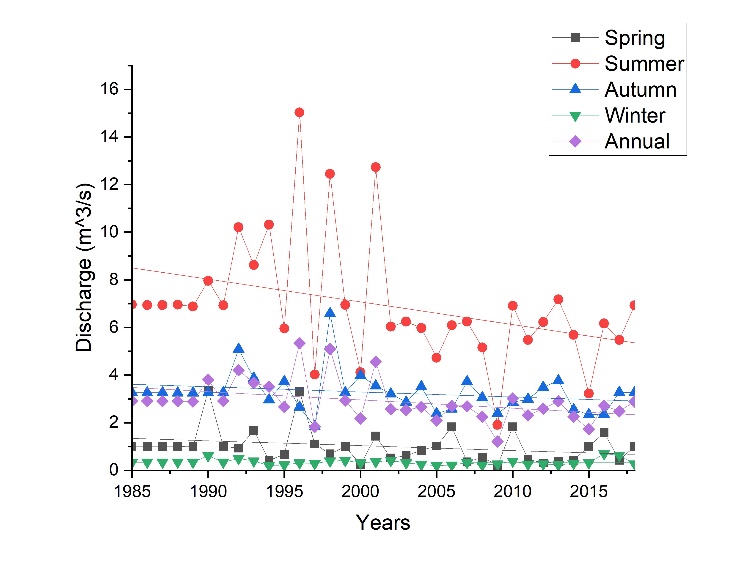  (I) | 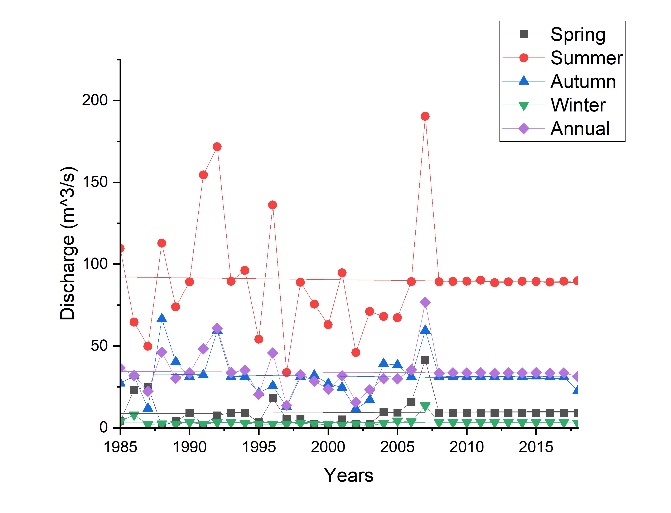  (J) |

(G) Gojeb (H) Guma (I) Megech (J) Wabi

Figure 19 Time series plot of annual and seasonal streamflow

Figure 20 Result of ITA for spring streamflow

Figure 21 Result of ITA for summer streamflow

Figure 22 Result of ITA for autumn streamflow

Figure 23 Result of ITA for winter streamflow

Figure 24 Result of ITA for annual streamflow
